# Effects of phylogenetic uncertainty on fossil identification illustrated by a new and enigmatic Eocene iguanian

**DOI:** 10.1038/s41598-020-72509-2

**Published:** 2020-09-25

**Authors:** Simon G. Scarpetta

**Affiliations:** Department of Geological Sciences, Jackson School of Geosciences, The University of Texas at Austin, Austin, TX USA

**Keywords:** Palaeontology, Phylogenetics, Taxonomy

## Abstract

Fossil identifications made in a phylogenetic framework are beholden to specific tree hypotheses. Without phylogenetic consensus, the systematic provenance of any given fossil can be volatile. Paleobiogeographic and divergence time hypotheses are contingent on the accurate systematic placement of fossils. Thus, fossil diagnoses should consider multiple topologies when phylogenetic resolution or clear apomorphies are lacking. However, such analyses are infrequently performed. Pleurodonta (Squamata: Iguania) is an ancient and frequently-studied lizard clade for which phylogenetic resolution is notoriously elusive. I describe a skull fossil of a new pleurodontan lizard taxon from the Eocene deposits of the Willwood Formation, Wyoming, and use the new taxon as a case-study to explore the effects of phylogenetic uncertainty on fossil identification. The relationships of the new taxon differ considerably among analyses, and resulting interpretations are correspondingly disparate. These results illustrate generalizable and severe issues with fossil interpretations made without consideration of alternative phylogenetic hypotheses.

## Introduction

Fossils provide inimitable data about past life, but those data can only be unlocked if fossils are accurately identified. The use of phylogenetically explicit identification methods (e.g., phylogenetic analyses or apomorphy-based approaches) allows fossils to inform divergence times and biogeographic hypotheses^1, 2^. Logically, identifications of fossils in a phylogenetic framework are entirely tree dependent; because there is no single consensus of the Tree of Life, systematic interpretations of most fossils are generally tied to individual tree topologies estimated by analyses of specific datasets^1^. Thus, a single systematic interpretation for any given fossil is unlikely. Optimization of morphological character evolution to topological hypotheses more consistent with molecular data is known to alter the phylogenetic placement of fossils^3^, and paleontological studies of diverse animal groups, including mammals^4^, turtles^5^, and birds^6^, indicated that molecular scaffold analyses change and improve fossil placement. Molecular scaffolds improve fossil placement the most when morphological data exhibit high homoplasy^3, 6^, and should also prevent overinterpretation of fossils when phylogenetic resolution is lacking.

The iguanian lizard clade Pleurodonta (anoles, horned lizards, Madagascan iguanas, and relatives) is well-studied by both paleontologists and neontologists and is a frequent subject of phylogenetic research. Crown Pleurodonta is hypothesized to have rapidly radiated during the Cretaceous^7^, and the group has repeatedly repelled efforts to resolve the relationships among its major clades^8^. There are numerous hypotheses of those relationships among analyses of molecular, morphological, and combined-evidence datasets^7–17^. Unsurprisingly, systematic assessments of fossils identified as pleurodontans have varied substantially, particularly for fossils collected from Cretaceous and early Cenozoic sediments^9–12, 14, 15^. Because there are so many different hypotheses of pleurodontan relationships, and hypotheses are broadly inconsistent among morphological datasets and between morphological and molecular analyses^8, 9, 11^, topological hypothesis choice is liable to change the phylogenetic placement of fossil pleurodontans. However, few attempts have been made to synthesize evidence from multiple topological hypotheses to identify fossil pleurodontans. Moreover, most recent phylogenetic treatments that included fossil pleurodontans were based on analyses of morphological datasets created to resolve the higher-order phylogeny of Squamata^9–12, 14^ as opposed to datasets designed to infer the phylogeny of Iguania or Pleurodonta^15^.

Here, I illustrate the effects of considering multiple topological hypotheses on fossil diagnosis and identification. I describe a new fossil pleurodontan from the early Eocene Willwood Formation in the Bighorn Basin, Wyoming, and I address the effects of three molecular scaffolds^7, 8, 17^ on the systematic diagnosis of that fossil. I use two phylogenetic matrices^14, 15^ and both parsimony and Bayesian methods to validate my results, and perform Bayesian hypothesis testing to evaluate support for two alternative hypotheses of the phylogenetic relationships of the new taxon.

## Results

### Locality and geologic setting

The fossil was collected during the 1971 Yale University Bighorn Basin Expedition at Yale Peabody Museum (YPM) locality 24 and is reposited at YPM. YPM 24 is in Park County, Wyoming, near the top of the Willwood Formation, 611 m above the base of the formation^18^ (690 m above the base of the Elk Creek section^19^). YPM 24 is in the Upper *Heptodon* Range Zone described by Schankler^19^ in Wa7 of the Wasatchian North American land mammal age, given the presence of *Lambdotherium* in strata below YPM 24^19–21^. I did not find locality data specific to YPM 24 besides basic stratigraphic and mapping information^18, 19^, and no additional information was found at the YPM (D. L. Brinkman pers. comm.). The Willwood Formation contains sandstones and mudstones, which are variably overprinted by paleosols^18^. The base of the Willwood Formation is at the carbon isotope excursion at the Paleocene–Eocene boundary at 56 Ma^22^. A tuff near the top of the formation at 634 m and corresponding with chron C24n.1n was dated to 52.8 ± 0.3 Ma by ^40^Ar/^39^Ar dating^23^, but was re-dated via the ^40^Ar/^39^Ar dating method to 52.59 ± 0.12 Ma^24^. The minimum age of the fossil is 52.47 Ma based on those dates.

### Systematic paleontology

- Squamata Oppel, 1811^25^
- Iguania Cuvier, 1817^26^
- Pleurodonta Cope, 1864^27^ (= Iguanidae sensu Schulte et al.^13^ and = Iguanoidea sensu Daza et al.^10^)
- *Kopidosaurus perplexus* gen. et. sp. nov.

### Etymology

*‘Kopis*’ (genitive ‘*kopidos*’; Greek) a knife with a curved blade used in ancient Greece; ‘*sauros*’ (Greek) lizard; per + ‘*pleko*’ (Greek) to twist. The genus name describes the sharp and recurved mesial maxillary teeth, and the species name refers to the unclear phylogenetic position of the new taxon.

### Holotype

**Figure 1:**
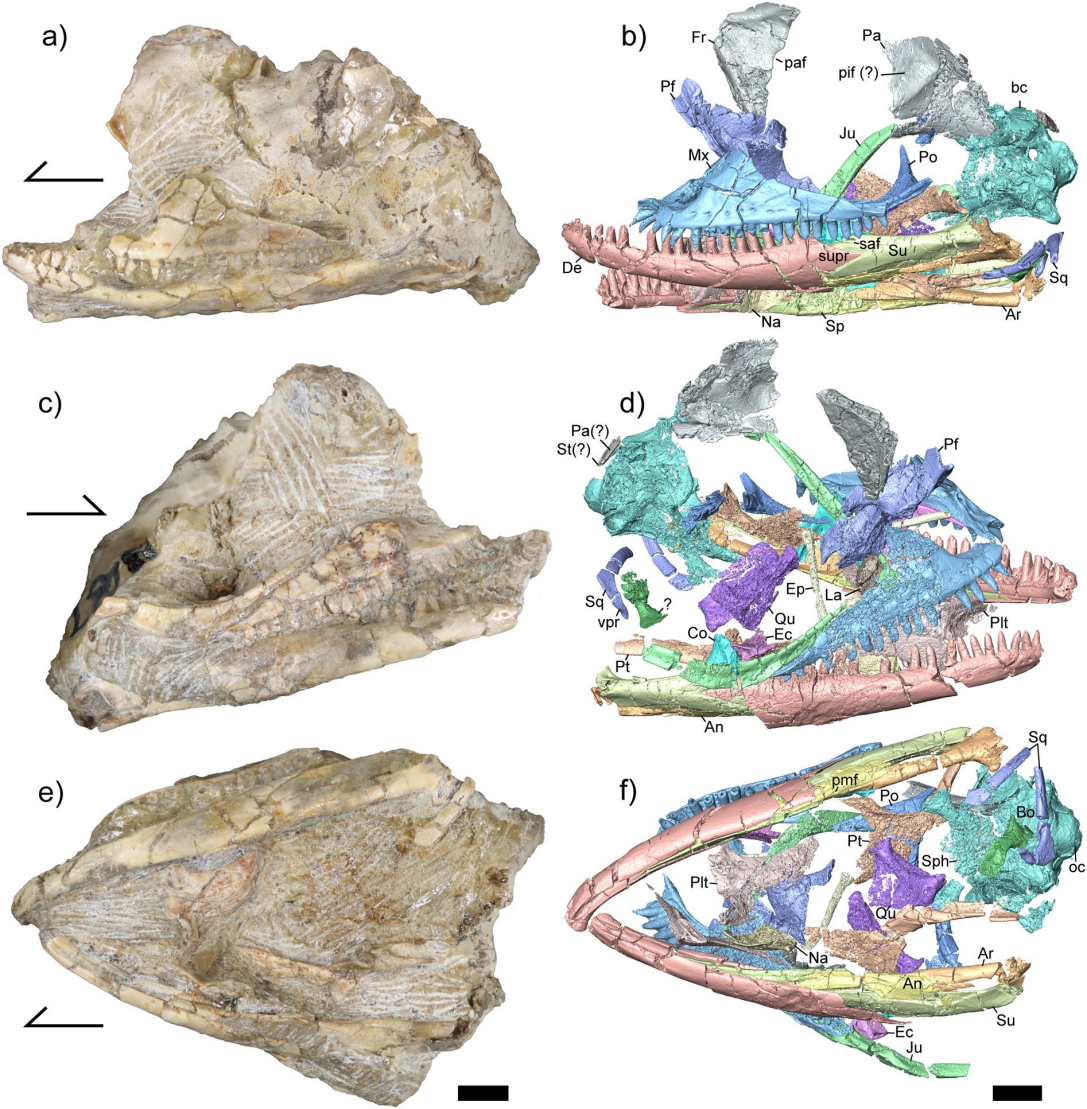
Holotype (YPM VP 8287) of *Kopidosaurus*. Images on the left are of the physical specimen and those on the right are digital reconstructions. Arrows face anteriorly. (**a**,**b**) Skull in left lateral view. (**c**,**d**) Skull in right lateral view. (**e**,**f**) Skull in ventral view. Anatomical abbreviations: An, angular; Ar, articular; bc, braincase; Bo, basioccipital; Co, coronoid; De, dentary; Ec, ectopterygoid; Ep, epipterygoid; Fr, frontal; Ju, jugal; La, lacrimal; Mx, maxilla; Na, nasal; oc, occipital condyle; Pa, parietal; Pf, prefrontal; paf, parietal foramen; pif, pineal foramen; Plt, palatine; pmf, posterior mylohyoid foramen; Po, postorbital; Pt, pterygoid; Qu, quadrate; Sph, sphenoid; Sp, splenial; Sq, squamosal; St, supratemporal; Su, surangular; supr, surangular process of the dentary; vpr, ventral process of the squamosal. Scale bars, 2 mm.

**Figure 2:**
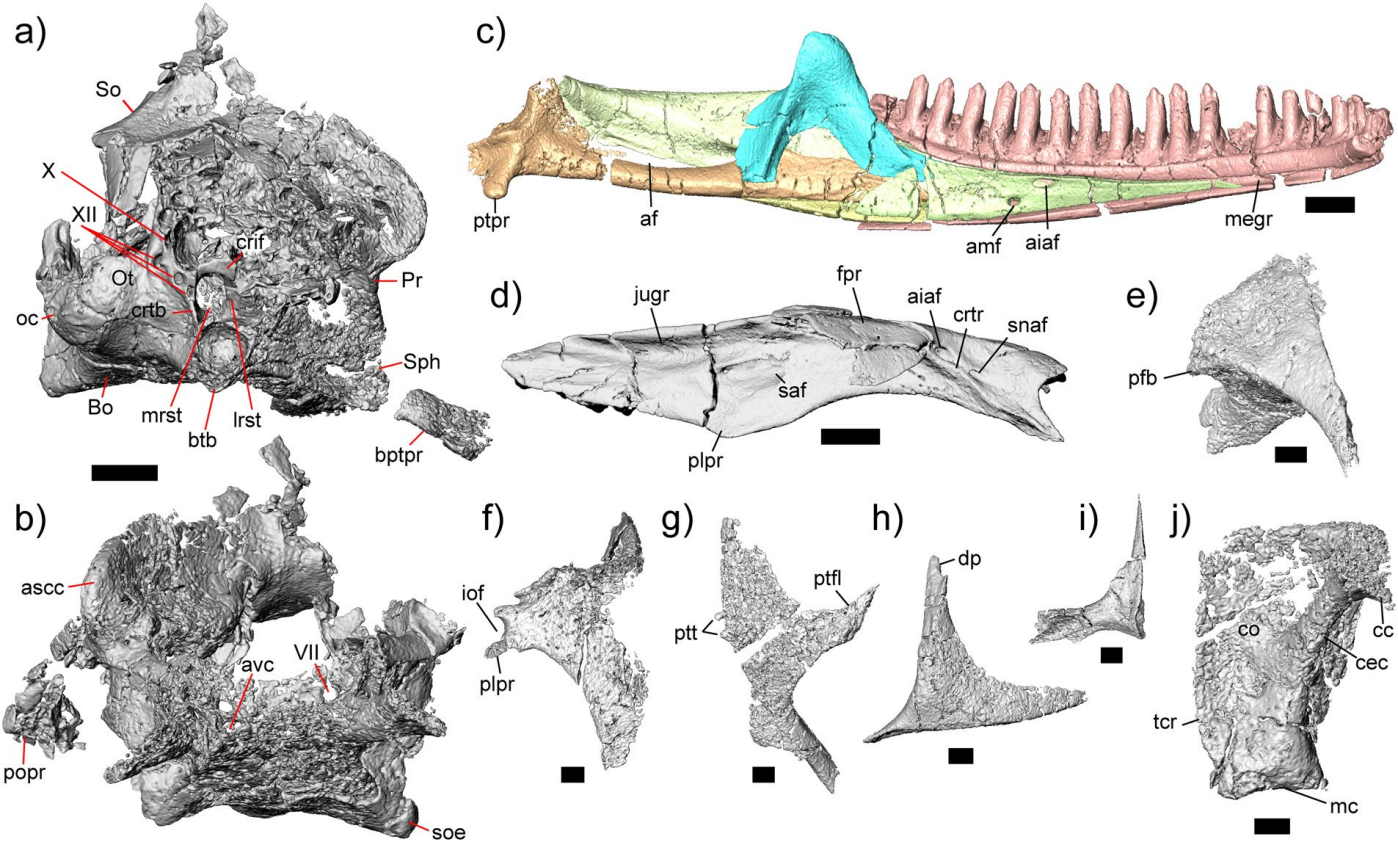
Select cranial elements of *Kopidosaurus*. All images are digital reconstructions. Braincase in (**a**) right posterolateral view and (**b**) anterior view. (**c**) Left mandible in lingual view. (**d**) Left maxilla in dorsal view. (**e**) Left prefrontal in dorsal view. (**f**) Left palatine in dorsal view. (**g**) Left pterygoid in ventral view. (**h**) Left postorbital in dorsal view. (**i**) Right ectopterygoid in dorsal view. (**j**) Left quadrate in posterior view. Anatomical abbreviations: af, adductor fossa; aiaf, anterior inferior alveolar foramen; amf, anterior mylohyoid foramen; ascc, anterior semicircular canal; avc, anterior vidian canal; Bo, basioccipital; bptpr; basipterygoid process; btb, basal tubercle; cc, cephalic condyle; cec, central column; co, conch; crif, crista interfenestralis; crtb, crista tuberalis; crtr, crista transversalis; dp, dorsal process; fpr, facial process; iof, infraorbital foramen; jugr, jugal groove; lrst, lateral aperture for the recessus scalae tympani; mc, mandibular condyle; megr, Meckelian groove; mrst, medial aperture for the recessus scalae tympani; Ot, otooccipital; pfb, prefrontal boss; plpr, posterolateral palatine process; popr, paroccipital process; ptfl, pterygoid flange; ptpr, pterygoideus process; ptt, pterygoid teeth; saf, superior alveolar foramen; Sph, sphenoid; snaf, subnarial arterial foramen; So, supraoccipital; soe, sphenoccipital epiphysis; tcr, tympanic crest; VII, abduscent foramen; X, vagus foramen; XII, hypoglossal foramina. Scale bars, 1 mm (**a–d**), 0.5 mm (**e–j**).

YPM VP (vertebrate paleontology) 8287, a mostly complete skull (Figs. 1, 2).

### Diagnosis

*Kopidosaurus* can be assigned to Lepidosauria based on pleurodont tooth implantation^28^, and to Squamata because it retains a splenial^29^. *Kopidosaurus* is referred to Iguania because the parietal foramen is partly bounded by the frontal (Fig. 1b) and the specimen has a prefrontal boss; the former occurs only in Iguania and the latter also occurs in Teiinae (Fig. 2e)^11, 30^. *Kopidosaurus* and crown Pleurodonta are distinguished from all other squamates by possessing separate subnarial arterial and anterior inferior alveolar foramina on the premaxillary process of the maxilla (Fig. 2d)^15^. *Kopidosaurus* differs from known crown pleurodontans in lacking a posterodorsal process of the squamosal (Fig. 1d,f), a reported apomorphy of Temujiniidae^11, 31^. *Kopidosaurus* differs from Temujiniidae in that the Meckelian groove is barely restricted, the splenial is not reduced anteriorly (Fig. 2c), and the postorbital has a long dorsal process for articulation with the parietal and possibly the frontal (Fig. 2h)^11, 15, 32^. *Kopidosaurus* lacks a frontoparietal fontanelle (Fig. 1b) and the infraorbital foramen is not contained within the palatine (Fig. 2f), which are apomorphies of the putative stem pleurodontan clade Isodontosauridae^10, 11^. YPM VP 8287 preserves no morphological feature or combination of features that would allow clear referral to any member of Pleurodonta.

### Description

Most cranial elements are at least partially preserved (Fig. 1), but the premaxilla and vomer are missing and the presence of the postfrontal and septomaxilla could not be ascertained. The right jugal, maxilla, and lacrimal are in articulation, as are the mandibles and braincase, but the rest of the elements are disarticulated and most are positionally displaced. Some bones are close to other elements with which they would articulate (e.g., the pterygoids, ectopterygoids, and left jugal). The dorsal surfaces of skull roof elements (e.g., the frontal and parietal) and the lateral surfaces of other elements (e.g., the dentary, maxilla, and jugal) lack sculpturing.

The left maxilla is almost completely preserved but lacks the posterior tip of the orbital process; the right maxilla is not as well preserved. The foramen for the superior alveolar nerve is large but does not occur within a groove on the dorsal surface of the palatal shelf (Fig. 2d). The palatal process lacks a pronounced medial expansion. There is a deep jugal groove on the dorsolateral surface of the orbital process, but no structure buttresses the groove. There are 17 tooth positions on both maxillae, but only 14 of those contain teeth on the right maxilla. There are eight and six labial nutrient foramina on the left and right maxilla, respectively. The facial process is dorsomedially inflected, and the anteroposterior dimension of the facial process is not markedly narrow. There is no labial sculpturing. The premaxillary process of the maxilla is short and is forked anteriorly. There are separate openings on its dorsal surface for the subnarial artery and the anterior inferior alveolar nerve (Fig. 2d). Small and slightly curved sheets of bone that may be pieces of the scleral ring are distributed near the maxillae, but several pieces are also located more posteriorly.

The jugal is gracile, particularly anteriorly (Fig. 1b–d). There is no quadratojugal process at the ventral inflection point of the orbit. The posterior portion of the left jugal is somewhat displaced, but it is evident that the temporal ramus is only slightly curved posteriorly. There is a minor exposure of the orbital process of the right jugal dorsal to the lateral wall of the maxilla. The right jugal appears to be slightly displaced posteriorly. The lacrimal is roughly rectangular in lateral view and has a ventrolateral articulation facet for the lateral wall of the maxilla. The prefrontal is triradiate, with a long and tapering dorsal process, an anterior process, and a posteroventral process. The articulation facet for the facial process of the maxilla is narrow. The prefrontal boss is well-developed and has a slightly rugose texture on its lateral surface. The left nasal is preserved, and is an ovoid element lacking a long anterior articulation facet for the premaxilla.

The posterior portion of the azygous frontal is present, including the articulation surface for the parietal and the posteriormost part of the interorbital region (Fig. 1b,d). The parietal foramen is preserved as a semicircular incision on the posterior margin of the bone. The preserved portion of the frontal lacks dorsal sculpturing, although the fragmentation of its right posterolateral margin makes it difficult to assess that area.

A substantial portion of the parietal table and the proximal part of the left postparietal process are preserved, but the anteriormost portion of the bone is missing (Fig. 1b). The right lateral portion of the bone is also missing. The posteromedial portion of the parietal table is fragmented such that it is not possible to conclusively determine the shape of the parietal table, but a posterior ‘v’ shape is evident. The preserved portion of the parietal lacks dorsal sculpturing. There is a piece of bone near the supraoccipital that appears to be a sliver of a postparietal process and an attached supratemporal. A recent report documented the first known occurrence of a fourth or pineal eye in squamates^33^. The parietal has a small but distinct foramen that fully perforates the bone and is located around the midline of the parietal table, which might represent a pineal foramen.

The palatines preserve vomerine, pterygoid, and maxillary processes. The facet for the maxilla is preserved only on the right palatine. The maxillary process of the left palatine has a distinct posterolateral projection that partially encloses the infraorbital foramen, but the dorsal portion of the maxillary process is broken on the right palatine. The choanal fossae are distinct and relatively deep, but are not long compared to the size of the palatine. There are no teeth on the ventral surface of the element. The right ectopterygoid is complete and has a long and straight anterior process to articulate with the orbital processes of the maxilla and the jugal (Fig. 2i); that process is missing on the left element. There are two projections at the posterolateral margin of the element that face posteroventrally and posterodorsally. The medial projection of the ectopterygoid is bifurcated to clasp the pterygoid flange. The pterygoids preserve the palatine flange, ectopterygoid process, and quadrate process, although the latter is broken posteriorly on both sides (Fig. 2h). The quadrate process is medially concave. There are two pterygoid teeth on the ventral surface of the right pterygoid (Fig. 2g), but teeth are not present on the left element. The pterygoid notch is a deeply concave shelf incised into the medial surface of the pterygoid ventral and anterior to the fossa columella. Two long and columnar epipterygoids are present. The quadrates are anteromedially displaced and are nearly complete (Fig. 2j).

The postorbital is triradiate, possessing an anterior process for articulation with the jugal, a long dorsal process to meet the parietal and potentially the frontal, and a posterior process to contact the squamosal (Fig. 2h). The dorsal process has a small posteriorly-facing spur, and the anterolateral face has a small but distinct tubercle. The posterior process is gradually tapered and is not expanded. The posterior projections of both squamosals are present, including a distinct posteroventral process. However, there is no posterodorsal process. The anterior portion is slender and is somewhat compressed mediolaterally.

The openings for the abducent foramen (cranial nerve VI) and the anterior vidian canal are evident in anterior view of the sphenoid (Fig. 2b), but the anterior openings of the carotid foramina were not preserved within the pituitary fossa. The basipterygoid processes are well-preserved, but a parasphenoid process was not identifiable. The basioccipital is complete. The basal tubercles are posteriorly located, almost to the level of the occipital condyle, and are well-developed and formed entirely by the basioccipital. A sphenoccipital epiphysis is evident on the left tubercle. Posteriorly, the basioccipital forms the ventral margin of the occipital condyle.

The supraoccipital is present, but the preservation of the bone is poor and it is broken in many places. The right prootic is present but the left element is essentially absent. The anterior semicircular canal is present and lacks an alar process. The prootic crest is moderately well-developed, extending anteroventrally almost to the sphenoid. The prootic has a supratrigeminal process in the incisura prootica. The lateral wall of the prootic is poorly preserved so it was not possible to determine the presence or position of a facial foramen.

The right otooccipital is essentially complete but the left is almost entirely missing. There are three hypoglossal (cranial nerve XII) foramina posterior to the lateral aperture for the recessus scalae tympani, and the vagus foramen (cranial nerve X) is a slender opening dorsal to the hypoglossal (Fig. 2a). The medial aperture for the recessus scalae tympani is subcircular and is present on both sides. Although the crista interfenestralis is present on the right otooccipital, the lateral wall is fragmented such that the position and size of the fenestra ovalis could not be determined. The crista tuberalis also is present ventral to the lateral aperture. The otooccipitals form the dorsolateral portions of the occipital condyle, which is semicircular in posterior view. The right paroccipital process is partially preserved but is detached from the braincase.

The dentaries are nearly complete. The meckelian groove is open for its entire length (Fig. 2c) and is marginally restricted by the dorsal curling of the inframeckelian lip (sensu^34^). There are 19 tooth positions and 17 teeth on the left dentary and 20 tooth positions and 18 teeth on the right dentary. The surangular process of the dentary is prominent but not elongate, and has a pointed posterior projection. The angular process is not as well-developed. The dentary contributes to over half of the total length of the mandible because the other mandibular elements are relatively short, but the dentary does not continue posteriorly far past the tooth row. There is no facet for a lateral process of the coronoid. Four and five nutrient foramina are present on the labial surface of the left and right dentaries, respectively. The intramandibular lamella is present but poorly-developed in both its dorsoventral and anteroposterior extent.

The splenial is anteriorly long and tapering, and encloses both the anterior inferior alveolar foramen of the mandible and the anterior mylohyoid foramen. The coronoid has four projections. The anterior process articulates medially with the splenial and laterally with the dentary, surangular, and articular, but does not extend far anteriorly into the Meckelian groove. The coronoid process is somewhat rounded dorsally and rests on the dorsal and medial surface of the surangular. The posteroventral process articulates with the surangular and articular medially and faces mostly ventrally. A medial crest descends from the coronoid process to the end of the posteroventral process and is oriented posteroventrally. The dorsolateral process is weak and barely articulates with the surangular.

Both surangulars are complete. There is a well-defined facet for the surangular process of the dentary on the anterolateral surface of the bone. The surangular foramen is located ventral and posterior to the articulations with the coronoid and the dentary, respectively. The articulars are largely preserved. The posterior projection of the retroarticular process is absent, but the pterygoideus process is present medial to the mandibular condyle. The adductor fossa is large. The anterior process is long and tapering, extending anteriorly past the third-to-last tooth position on both sides. The angulars are broken anteriorly and ventrally. The angular encloses the posterior mylohyoid foramen, and is slightly concave dorsally.

Teeth are pleurodont and heterodont with respect to crown morphology, and are mostly columnar. The distalmost tooth bases of both dentaries and maxillae and the mesialmost teeth of the maxillae are mesiodistally expanded. There is a slight mid-shaft swelling of some median teeth. Distal crowns are unflared and tricuspid, and mesial crowns are unicuspid and taper to a point. The mesial teeth of the maxilla are particularly recurved and sharp. On both the maxilla and dentary there are slightly to moderately recurved teeth throughout the tooth row, besides the distalmost teeth. Teeth are high-crowned, having a substantial exposure above the dorsal margin of the dentary relative to both the height of the tooth and to the height of the dentary. There are clear spaces in between adjacent teeth except for the mesialmost dentary teeth.

### Phylogenetic analyses

**Figure 3:**
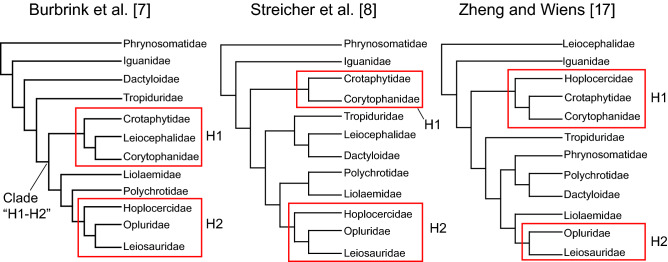
Molecular scaffolds used in this study with labelled main sister relationship hypotheses for *Kopidosaurus*.

Parsimony and Bayesian scaffold analyses of the dataset from^15^ with all fossils included provide two main hypotheses (Fig. 3, Table 1): *Kopidosaurus* is in a total clade containing some combination of **H1)** Corytophanidae, Crotaphytidae, and Hoplocercidae or **H2)** Opluridae, Leiosauridae, and Hoplocercidae. Analyses of the matrix from^14^ largely corroborated those results, although a sister relationship with only Opluridae, with (((Opluridae, Leiosauridae), Hoplocercidae), Polychrotidae) or with (((Opluridae, Leiosauridae), Hoplocercidae), (Polychrotidae, Liolaemidae)) occurred only with that matrix.

**Table 1:** Results of the phylogenetic analyses.

Fossil inclusion	Dataset	Method	Constraint			
			Ingroup	Burbrink et al.^7^	Streicher et al.^8^	Zheng and Wiens^17^
All fossils included	Smith^15^	Parsimony	((Polychrotidae, Dactyloidae), (Leiosauridae, Corytophanidae, (Iguanidae, Hoplocercidae)))	Hoplocercidae	((Opluridae, Leiosauridae), Hoplocercidae) polytomy	((Corytophanidae, Crotaphytidae), Hoplocercidae)
	Smith^15^	Bayesian	Hoplocercidae	Hoplocercidae	Corytophanidae	(Corytophanidae, Crotaphytidae)
	Simoes^14^	Parsimony	Opluridae	Clade "H1-H2" polytomy (see Fig. 3)	Opluridae	(Corytophanidae, Crotaphytidae)
	Simoes^14^	Bayesian	(Corytophanidae, Crotaphytidae)	(((Opluridae, Leiosauridae), Hoplocercidae), Polychrotidae)	(((Opluridae, Leiosauridae), Hoplocercidae), (Polychrotidae, Liolaemidae))	(Corytophanidae, Crotaphytidae)
YPM VP 8287 is only fossil	Smith^15^	Parsimony	(((Polychrotidae, Dactyloidae), Leiosauridae), (Corytophanidae, Hoplocercidae))	Clade "H1-H2" polytomy (see Fig. 3)	All pleurodontans besides Phrynosomatidae and Iguanidae polytomy	((Corytophanidae, Crotaphytidae), Hoplocercidae)
	Smith^15^	Bayesian	Iguania	Hoplocercidae	Hoplocercidae	Hoplocercidae
	Simoes^14^	Parsimony	Pleurodontans besides Phrynosomatidae polytomy	Clade "H1-H2" polytomy (see Fig. 3)	Opluridae	(Corytophanidae, Crotaphytidae)
	Simoes^14^	Bayesian	(Corytophanidae, Crotaphytidae)	(((Opluridae, Leiosauridae), Hoplocercidae), Polychrotidae)	((Opluridae, Leiosauridae), Hoplocercidae)	(Corytophanidae, Crotaphytidae)

The content of each cell is the sister clade of *Kopidosaurus* from that analysis permutation, written in Newick format when appropriate.

Sister relationships or polytomies with a larger group of pleurodontan clades occurred in parsimony analyses of both matrices with the ingroup constraint or with the scaffolds based on^7^ or^8^. The analyses in which *Kopidosaurus* was sister to many pleurodontans might indicate that *Kopidosaurus* represents an extinct and previously undescribed clade of pleurodontan, but could also imply that *Kopidosaurus* does not preserve specific features that would allow placement in a less inclusive and/or known clade. Because that type of hypothesis appeared mostly in sensitivity analyses and because reasonable alternative hypotheses could not be constructed between scaffold and ingroup constraint analyses, I did not pursue them with stepping-stone analyses. Most other sensitivity analyses estimated similar relationships to those with all fossils.

Stepping-stone analyses offered little insight into the viability of the two main hypotheses (Table 2). Neither hypothesis was favored in analyses of the scaffold from^8^, but H2 was slightly favored over H1 for those based on^7^. H1 was strongly favored over H2 in analyses with the scaffold from^17^. Neither hypothesis can be rejected based on those analyses.

**Table 2:** Result of stepping stone analyses of the dataset from^15^.

Scaffold	Hypothesis	Constraint	Marg. lik	2ln_e_BF
**Burbrink et al.**^7^	**H2**	**Hoplocercidae + Opluridae + Leiosauridae**	**− 2,410.30**	**–**
Burbrink et al.^7^	H1	Corytophanidae + Crotaphytidae + Leiocephalidae	− 2,411.66	2.72
Streicher et al.^8^	H1	Corytophanidae + Crotpahytidae	− 2,396.87	–
Streicher et al.^8^	H2	Hoplocercidae + Opluridae + Leiosauridae	− 2,397.61	1.48
**Zheng and Wiens**^17^	**H1**	**Hoplocercidae + Corytophanidae + Crotaphytidae**	**− 2,379.34**	**–**
Zheng and Wiens^17^	H2	Opluridae + Leiosauridae	− 2,386.10	13.52

The favored hypothesis for each scaffold is in bold. The 2log_e_BF values are for the favored hypothesis relative to the given hypothesis. Marg. lik. = marginal likelihood.

## Discussion

Despite the apparent reversal of an apomorphy of crown Pleurodonta (presence of a posterodorsal process of the squamosal), support for *Kopidosaurus* as a member of crown Pleurodonta was unanimous in analyses in which all fossils were included, and nearly so in sensitivity analyses. Within crown Pleurodonta, however, the phylogenetic placement of *Kopidosaurus* was predominately tied to tree topology, but was also affected by matrix choice and analytical method (Table 1). Stepping-stone analyses did not support either H1 or H2. Additionally, there is the possibility that *Kopidosaurus* represents a previously undescribed clade.

The uncertainty of the relationships of *Kopidosaurus* is due in part to the mosaic morphology of the fossil and the problematic nature of pleurodontan phylogeny. Nevertheless, these analyses illustrate a problem that is not confined to the present study: Alternative topological hypotheses, particularly those derived from molecular datasets, can substantially alter phylogenetic interpretations of fossils. Moreover, sister taxon hypotheses for *Kopidosaurus* varied among the relatively similar molecular scaffolds derived from targeted sequence capture data^7, 8^, and between those two hypotheses and the scaffold based on^17^, which is derived from Sanger-sequenced loci (see Fig. 3). Researchers who have discussed the effects of tree topology on fossil identification usually contrast morphological against molecular hypotheses^1^. The analyses here emphasize that differences among molecular hypotheses can also produce discrepant fossil placements [but see^6^], a point that is increasingly important. Next-generation sequence data (i.e., targeted sequence capture datasets) are now ubiquitous, and it is readily apparent that phylogenetic hypotheses based on those new data can differ substantially from hypotheses based on traditional Sanger-sequenced loci^7, 8, 17^. With that in mind, the results of the present study underscore the importance of testing alternative hypotheses when using phylogenetic analyses to identify fossils (e.g.^35^). I reiterate the recommendation from^36^ that molecular scaffolds of well-supported nodes should be employed to improve fossil identification, or, when resolution is lacking, multiple scaffolds should be tested, as was done here.

Researchers who mine the paleontological literature for fossils to use in node calibrations should be mindful of these problems, especially when studying clades that lack phylogenetic resolution. Specifically, consideration should be given to whether a fossil is appropriate for the intended calibration regardless of the author’s preferred tree topology^1^, and whether it was identified via analysis of the most suitable phylogenetic matrix^37, 38^. For example, although the matrix from^14^ has over twice as many characters as the one from^15^, the latter matrix had as many as 20 more parsimony-informative characters for addressing pleurodontan phylogeny and fossil placement (Table S1). Thus, the dataset from^14^ is probably not the most appropriate dataset for the present study. Several fossil iguanians from the Late Cretaceous of North America were diagnosed by phylogenetic analysis of matrices created primarily to assess relationships among squamates (e.g.^30, 39^). Systematic reassessments of those fossils with more targeted matrices could prove fruitful.

Scaffold parsimony analyses with all fossils produced most-parsimonious trees (MPTs) with > 45 more steps than the MPTs with no scaffold (Table S2), indicating potential homoplasy across Pleurodonta. Apomorphy lists for analyses of the dataset from^15^ revealed that, depending on the scaffold, several of the same morphological features placed *Kopidosaurus* within a total clade containing some combination of ((Opluridae, Leiosauridae), Hoplocercidae) or (Hoplocercidae, (Corytophanidae, Crotaphytidae)). Those features include partial enclosure of the infraorbital foramen by the posterolateral process of the palatine (character 31, 2–> 1), opening of the Meckelian groove (character 94, 2– > 0), development of the intramandibular lamella (character 96, 0– >  1), and anterior extent of the angular (character 103, 0–> 1). Closure of the Meckelian groove without fusion is derived in Pleurodonta^11^, and because the dentary is a frequently collected and recognized element among lizard fossils, that feature is often used to identify fossil pleurodontans. However, an open and almost completely unrestricted groove like that of YPM VP 8287 occurs in *Phymaturus*, Hoplocercidae, *Crotaphytus*, and Phrynosomatidae (specimens examined in ESM file 1), indicating several independent reversals of that feature. Evaluating multiple topological hypotheses will help identify homoplastic features that confound fossil identification^6^.

There are several biogeographic scenarios given the potential relationships of *Kopidosaurus*. Corytophanids are known from middle latitudes of North America during the Eocene^40^ and crotaphytids occur in middle latitudes of North America currently, so the discovery of a taxon related to either or both of those clades is not unexpected. There is some evidence that hoplocercids were present in North America during the Eocene and Cretaceous^39, 41^, but the identifications of those fossils are tentative and the biogeographic ramifications of such a discovery are unexplored; Hoplocercidae currently occurs in South America and Panama. Extant Opluridae and Leiosauridae are also geographically distant from the Willwood Formation, occurring in Madagascar and South America, respectively, but one putative leiosaurid fossil was previously described from the Eocene of Wyoming^42^. The occurrence of a taxon related to Opluridae and/or Leiosauridae, while surprising given the modern biota, has some precedent. There is a broad range of biogeographic implications given a close relationship between *Kopidosaurus* and any of those taxa, but interpretations of a relationship with Opluridae and/or Leiosauridae relative to Corytophanidae and Crotaphytidae are especially divergent.

*Kopidosaurus* is not known to occur in previously described faunas from earlier Eocene deposits in the Willwood Formation^40, 43^. Regional temperatures rose in the late early Eocene around the time of deposition of the fossil^44^, but it is not clear if the evolution of *Kopidosaurus* is associated with climate change, or whether its appearance represents an immigration event or in situ diversification. Given the phylogenetic volatility of *Kopidosaurus*, I refrain from favoring any biogeographic or divergence hypothesis based on the identification of the fossil and advise similar caution for other systematically enigmatic fossils, lizard or otherwise.

## Methods

### Nomenclature

Anatomical nomenclature follows^15^.

### High-resolution computed tomography (CT) scanning

YPM VP 8287 was CT scanned at the University of Texas at Austin High-resolution Computed Tomography Facility (UTCT) on a NSI scanner with a Fein Focus High Power Source. There are 1909 slices in the XY plane, and the voxel size is 11.7 μm. Digital segmentation was performed in Avizo Lite 2019 on 16 bit TIFFs using the magic wand tool with gray scale values over 30,000. Manual selections were occasionally necessary to separate elements or to differentiate bone from matrix. Some bony materials were too fragmentary to be identified. All CT images are surface renderings in orthographic view.

### Phylogenetic analyses

To assess the relationships of *Kopidosaurus*, I selected and scored YPM VP 8287 in a morphological matrix created to infer iguanian relationships^15^. To validate those analyses, I scored the fossil in a recently published dataset designed to assess lepidosauromorph relationships^14^. See Table S2 for statistics about each matrix. Apomorphy-based diagnosis established that YPM VP 8287 was an iguanian and a pleurodontan (see above). The dataset from^14^ contains non-lepidosaurian outgroups and non-iguanian squamates that were unnecessary for placing YPM VP 8287 and that would have created difficulties establishing consistent molecular scaffolds, so I removed most of those taxa, leaving only rhyncocephalian, scincid, eublepharid, and anguid outgroups similar or identical to those in^15^. That approach allowed the two datasets to be more compatible taxonomically.

I evaluated the position of YPM VP 8287 with three molecular scaffolds (Fig. 3). The scaffolds were derived from analyses of targeted sequence capture datasets^7, 8^ or Sanger-sequenced mitochondrial and nuclear genes^17^. Monophyly of family-level pleurodontan clades and the relationships of those clades to each other were constrained for each scaffold, but intrafamily relationships could vary. Acrodonta was constrained as sister to Pleurodonta, and relationships of other squamate outgroups follow^7^. I also performed essentially unconstrained analyses in which only the monophyly of the ingroup (Iguania) was enforced. All fossils besides the rhyncocephalian *Diphydontosaurus avonis* could attach to anywhere on the tree in all analyses. Sensitivity analyses in which YPM VP 8287 was the only fossil were run for all analysis permutations.

I augmented the matrix from^14^ to include all family-level crown pleurodontan clades by scoring specimens of *Basiliscus vittatus* and *Anolis sagrei* (specimen numbers in ESM file 1). I changed two character scores for *Crotaphytus collaris*; character 97 (palatine teeth) is now coded as polymorphic, and character 170 (contact of the dorsal and ventral margins of the dentary) is now coded as absent (specimens examined in ESM file 1).

Parsimony analyses were conducted in PAUP 4.0^45^ with a heuristic search and 10,000 replicates, random taxon addition, multistate codings treated as polymorphic, and branches collapsed if maximum length equals zero. Bayesian analysis was performed in MrBayes v 3.2.7^46^ for 2,000,000 generations, with two runs and four chains, the gamma parameter, a symmetric dirichlet hyperprior with α fixed at infinity, sampling every 1,000 generations and with a burnin of 25%. Convergence was assessed by checking that ESS (effective sample size) values were above 200 in Tracer 1.7^47^. All characters in all analyses were treated as unordered and equally weighted. Results were summarized as strict consensus trees for parsimony analyses and 50% majority rule consensus trees for Bayesian analyses. MrBayes analyses were performed on the CIPRES computer cluster^48^. Apomorphy lists were generated in PAUP for the parsimony and Bayesian scaffold analyses of the dataset from^15^ with all fossils (in ESM file 1).

### Bayesian hypothesis testing with stepping-stone analysis

Scaffold analyses of the dataset published from^15^ inferred two main hypotheses of the phylogenetic relationships of *Kopidosaurus* (Table 1): *Kopidosaurus* is in a total clade containing a permutation of **H1)** Corytophanidae, Crotaphytidae, and Hoplocercidae or **H2)** Opluridae, Leiosauridae, and Hoplocercidae. Stepping-stone analyses with the dataset from^15^ were used to determine support for the two sister taxon hypotheses. In stepping-stone analyses *Kopidosaurus* was constrained to be within the total clade associated with either H1 or H2. In the topology inferred by^7^, Leiocephalidae is nested within (Crotaphytidae, Corytophanidae), so the alternative hypothesis for that scaffold includes Leiocephalidae. In the topology inferred by^17^, Hoplocercidae is sister to (Crotaphytidae, Corytophanidae), so the alternative hypothesis for that scaffold includes only (Opluridae, Leiosauridae).

Analyses were performed for four runs of 10,00,000 total generations, with 100 steps and 100,000 generations per step, two chains, α of 0.3, and a burnin of − 1. The Bayes Factor (BF) statistic 2log_e_BF was used to compare support for each model. Hypothesis support was interpreted as strongly favored when 2log_e_BF > 10, favored when 2log_e_BF ≥ 6 and < 10, slightly favored when 2log_e_BF ≥ 2 and < 6, and negligible when 2log_e_BF < 2 (adapted from^49^).

## Supplementary information

Supplementary Information 1.

Supplementary Dataset S1.

Supplementary Dataset S2.

Supplementary Dataset S3.

Supplementary Dataset S4.

## Data Availability

Phylogenetic datasets are published with this article. Supplementary figures (Figs. S1–S32) and tables are in the Supplementary Information file. The raw CT slice data are deposited at MorphoSource.org at https://www.morphosource.org/Detail/ProjectDetail/Show/project_id/1066. The dataset from^14^ was downloaded from the electronic version of that publication at Nature.com. The dataset from^15^ is in the in-text appendix of that publication.
