# Supplementary material for: Effects of phylogenetic uncertainty on fossil identification illustrated by a new and enigmatic Eocene iguanian
Source: Sci Rep. 2020 Sep 25;10:15734. doi: 10.1038/s41598-020-72509-2 (PMC7519069; doi:10.1038/s41598-020-72509-2)
Supplement: Supplementary file 1 — Supplementary Information 1. [file 41598_2020_72509_MOESM1_ESM.docx]

**Supplementary Information for: Effects of phylogenetic uncertainty on fossil identification illustrated by a new and enigmatic Eocene iguanian**

Simon G. Scarpetta

Department of Geological Sciences, Jackson School of Geosciences, The University of Texas at Austin

**Supplemental Information**

**Index**

**1. Abbreviations**

**2. Specimen numbers of taxa scored for the matrix from Simões et al. [1]**

**3. Additional specimens examined**

**4. Dataset legends**

**5. Supplemental figures**

**6. Supplemental tables**

**7. Apomorphy lists**

**8. References**

**1. Abbreviations**

**CAS** California Academy of Sciences, San Francisco, California

**mpt** Most parsimonious tree

**MVZ** Museum of Vertebrate Zoology, University of California, Berkeley, California

**TxVP** Texas Vertebrate Paleontology, The University of Texas at Austin, Austin, Texas (formerly TMM)

**UCMP** University of California Museum of Paleontology, University of California, Berkeley, California

**2. Specimen numbers of taxa scored for the matrix from [1]**

*Anolis sagrei*: TxVP M-9043, M-9044

*Basiliscus vittatus:* CAS uncatalogued; TxVP M-9086

**3. Additional specimens examined**

*Callisaurus draconoides* TxVP M-8649, M-14320

*Crotaphytus collaris* TxVP M-9255; UCMP 117152

*Hoplocercus spinosus* CAS 231483

*Morunasaurus groi* CAS 98235

*Oplurus cuvieri* CAS 231434

*Phymaturus palluma* MVZ Herp 9209

**4. Dataset legends**

**Dataset S1.** Dataset from Smith [2] and character scores for YPM VP 8287 in that dataset, formatted for Bayesian analyses.

**Dataset S2.** Dataset from Smith [2] and character scores for YPM VP 8287 in that dataset, formatted for parsimony analyses.

**Dataset S3.** Modified dataset from Simões et al. [1] and character scores for YPM VP 8287 in that dataset, formatted for Bayesian analyses.

**Dataset S4.** Modified dataset from Simões et al. [1] and character scores for YPM VP 8287 in that dataset, formatted for parsimony analyses.

**5. Supplemental figures**

**Figure S1.** Bayesian analysis of dataset from [2] with scaffold based on [3] with all fossils.

**Figure S2.** Bayesian analysis of dataset from [2] with scaffold based on [3] and in which YPM VP 8287 is the only fossil.

**Figure S3.** Parsimony analysis of dataset from [2] with scaffold based on [3] with all fossils.

**Figure S4.** Parsimony analysis of dataset from [2] with scaffold based on [3] and in which YPM VP 8287 is the only fossil.

**Figure S5.** Bayesian analysis of dataset from [2] with ingroup constraint.

**Figure S6.** Bayesian analysis of dataset from [2] with ingroup constraint.

**Figure S7.** Parsimony analysis of dataset from [2] with ingroup constraint.

**Figure S8.** Parsimony analysis of dataset from [2] with ingroup constraint.

**Figure S9.** Bayesian analysis of dataset from [2] with scaffold based on [4] with all fossils.

**Figure S10.** Bayesian analysis of dataset from [2] with scaffold based on [4] and in which YPM VP 8287 is the only fossil.

**Figure S11.** Parsimony analysis of dataset from [2] with scaffold based on [4] with all fossils.

**Figure S12.** Parsimony analysis of dataset from [2] with scaffold based on [4] and in which YPM VP 8287 is the only fossil.

**Figure S13.** Bayesian analysis of dataset from [2] with scaffold based on [5] with all fossils.

**Figure S14.** Bayesian analysis of dataset from [2] with scaffold based on [5] and in which YPM VP 8287 is the only fossil.

**Figure S15.** Parsimony analysis of dataset from [2] with scaffold based on [5] with all fossils.

**Figure S16.** Parsimony analysis of dataset from [2] with scaffold based on [5] and in which YPM VP 8287 is the only fossil.

**Figure S17.** Bayesian analysis of dataset from [1] with scaffold based on [3] with all fossils.

**Figure S18.** Bayesian analysis of dataset from [1] with scaffold based on [3] and in which YPM VP 8287 is the only fossil.

**Figure S19.** Parsimony analysis of dataset from [1] with scaffold based on [3] with all fossils.

**Figure S20.** Parsimony analysis of dataset from [1] with scaffold based on [3] and in which YPM VP 8287 is the only fossil.

**Figure S21.** Bayesian analysis of dataset from [1] with ingroup constraint.

**Figure S22.** Bayesian analysis of dataset from [1] with ingroup constraint.

**Figure S23.** Parsimony analysis of dataset from [1] with ingroup constraint.

**Figure S24.** Parsimony analysis of dataset from [1] with ingroup constraint.

**Figure S25.** Bayesian analysis of dataset from [1] with scaffold based on [4] with all fossils.

**Figure S26.** Bayesian analysis of dataset from [1] with scaffold based on [4] and in which YPM VP 8287 is the only fossil.

**Figure S27.** Parsimony analysis of dataset from [1] with scaffold based on [4] with all fossils.

**Figure S28.** Parsimony analysis of dataset from [1] with scaffold based on [4] and in which YPM VP 8287 is the only fossil.

**Figure S29.** Bayesian analysis of dataset from [1] with scaffold based on [5] with all fossils.

**Figure S30.** Bayesian analysis of dataset from [1] with scaffold based on [5] and in which YPM VP 8287 is the only fossil.

**Figure S31.** Parsimony analysis of dataset from [1] with scaffold based on [5] with all fossils.

**Figure S32.** Parsimony analysis of dataset from [1] with scaffold based on [5] and in which YPM VP 8287 is the only fossil.

**6. Supplemental tables**

| **Dataset** | **Number of characters** | **Number of characters scored for YPM 8287** | **Number of parsimony informative characters (all terminal taxa)** | **Number of parsimony informative characters (YPM 8287 is only fossil)** |
| --- | --- | --- | --- | --- |
| **Smith [2]** | 152 | 62 | 145 | 143 |
| **Simões** ***et al*. [1]** | 347 | 155 (including gaps) | 129 | 113 |

**Table S1.** Summary statistics for each morphological matrix.

| **All terminal taxa** | | | | |
| --- | --- | --- | --- | --- |
|  | **Constraint** | | | |
| **Dataset** | **Ingroup** | **Burbrink *et al.*[3]** | **Streicher *et al*. [4]** | **Zheng and Wiens [5]** |
| **Smith [2]** | TL = 638, CI = 0.299, RI = 0.557, RC = 0.167, 3 MPTs | TL = 710, CI = 0.269, RI = 0.485, RC = 0.131, 2 MPTs | TL = 706, CI = 0.271, RI = 0.489, RC = 0.132, 6 MPTs | TL = 683, CI = 0.280, RI = 0.512, RC = 0.143, 6 MPTs |
| **Simões** ***et al.*[1]** | TL = 480, CI = 0.487, RI = 0.452, RC = 0.220, 12 MPTs | TL = 522, CI = 0.448, RI = 0.359, RC = 0.161, 30 MPTs | TL = 516, CI = 0.453, RI = 0.372, RC = 0.169, 2 MPTs | TL = 527, CI = 0.444, RI = 0.347, RC = 0.154, 2 MPTs |
|  |  |  |  |  |
| **YPM 8287 is only fossil** | | | | |
|  | **Constraint** | | | |
| **Dataset** | **Ingroup** | **Burbrink *et al.*[3]** | **Streicher *et al*. [4]** | **Zheng and Wiens [5]** |
| **Smith [2]** | TL = 608, CI = 0.308, RI = 0.548, RC = 0.168, 7 MPTs | TL = 681, CI = 0.275 RI = 0.469, RC = 0.129, 5 MPTs | TL = 679, CI = 0.275, RI = 0.472, RC = 0.130, 4 MPTs | TL = 654, CI = 0.286, RI = 0.498, RC = 0.143, 1 MPT |
| **Simões** ***et al.*[1]** | TL = 442, CI = 0.509, RI = 0.410, RC = 0.209, 22 MPTs | TL = 487, CI = 0.462, RI = 0.288, RC = 0.133, 8 MPTs | TL = 481, CI = 0.468, RI = 0.304, RC = 0.142, 1 MPT | TL = 493, CI = 0.456, RI = 0.272, RC = 0.124 1 MPT |

**Table S2.** Statistics and number of most parsimonious trees from parsimony analyses

**7. Apomorphy lists for analyses of data set from Smith [2] with all fossils**

Parsimony analysis with scaffold based on Burbrink et al. [3]

/-------- YPM 8287

/---------------75

| \-------- Enyaliodes oshaughnessyi

/------74

| | /---------------- Pristidactylus torquatus

| | |

| \-------73 /-------- Oplurus quadrimaculatus

/------71 \------72

| | \-------- Chalarodon madagascariensis

| |

/------70 \--------------------------------- Polychrus acutirostris

| |

| | /-------- Phymaturus palluma

| \-------------------------------69

| \-------- Liolaemus pictus

|

| /---------------- Basiliscus basiliscus

| |

/------68 /-------62 /-------- Corytophanes cristatus

| | | \------61

| | /------64 \-------- Laemanctus longpipes

| | | |

| | | | /-------- Geiseltaliellus maarius

| | /------65 \---------------63

| | | | \-------- Suzanniwanna patriciana

| | | |

/------60 \------67 \--------------------------------- Leiocephalus personatus

| | |

| | | /-------- Crotaphytus collaris

| | \-------------------------------66

| | \-------- Gambelia wislizenii

| |

| | /---------------- Stenocercus scapularis

| | |

/-------57 | +---------------- Microlophus occipitalis

| | \---------------------------------------59

| | | /-------- Tropidurus torquatus

| | \------58

| | \-------- Plica umbra

| |

/------56 | /-------- Anolis ricordi

| | \-------------------------------------------------------40

| | \-------- Anolis cristatellus

| |

| | /---------------- Dipsosaurus dorsalis

| | |

/------55 \--------------------------------------------------------42 /-------- Brachylophus fasciatus

| | \------41

| | \-------- Iguana iguana

| |

| | /---------------- Phrynosoma platyrhinos

| | |

| \----------------------------------------------------------------44 /-------- Petrosaurus thalassinus

| \------43

/------54 \-------- Sceloporus undulatus

| |

| | /-------- Physignathus cocincinus

| | /------45

| | | \-------- Agama agama

| | /-------46

/------53 | /------47 \---------------- Brookesia superciliaris

| | | | |

| | \-------------------------------------------------------48 \------------------------- Leiolepis belliana

| | |

/-------52 | \--------------------------------- Priscagama gobiensis

| | |

| | \-------------------------------------------------------------------------------------------------- Saichangurvel davidsoni

/------51 |

| | \---------------------------------------------------------------------------------------------------------- Elgaria multicarinata

/------50 |

| | \------------------------------------------------------------------------------------------------------------------- Plestiodon fasciatus

| |

| \--------------------------------------------------------------------------------------------------------------------------- Eublepharis macularius

49

+----------------------------------------------------------------------------------------------------------------------------------- Diphydontosaurus avonis

|

\----------------------------------------------------------------------------------------------------------------------------------- Sphenodon punctatus

Branch Character Steps CI Change

-------------------------------------------------------------------------

node_49 --> node_50 16 1 0.125 0 ==> 1

41 1 0.333 0 --> 1

53 1 0.111 0 ==> 1

54 1 0.500 0 ==> 1

64 1 0.250 1 ==> 0

83 1 0.333 0 --> 2

91 1 0.250 0 --> 1

92 1 0.250 0 --> 1

98 1 0.125 1 --> 0

99 1 0.125 1 ==> 0

106 1 0.111 0 --> 1

119 1 0.143 0 --> 1

122 1 0.111 0 --> 1

135 1 0.125 0 --> 1

node_50 --> node_51 27 1 0.200 0 --> 1

47 1 0.091 0 --> 1

88 1 0.222 0 --> 1

120 1 0.111 0 --> 1

129 1 0.250 0 --> 1

136 1 0.333 1 ==> 0

node_51 --> node_52 39 1 0.167 0 --> 1

59 1 0.167 1 ==> 0

68 1 0.250 0 --> 1

98 1 0.125 0 --> 1

106 1 0.111 1 --> 0

107 1 0.500 0 --> 1

109 1 0.250 1 --> 2

119 1 0.143 1 --> 0

123 1 0.143 0 --> 1

node_52 --> node_53 19 1 0.125 0 ==> 1

22 1 0.143 0 ==> 1

23 1 0.125 1 ==> 0

27 1 0.200 1 --> 0

43 1 0.500 0 ==> 1

44 1 0.500 0 ==> 1

47 1 0.091 1 --> 0

50 1 0.429 0 --> 1

53 1 0.111 1 --> 0

54 1 0.500 1 ==> 0

72 1 0.333 2 --> 0

79 1 0.400 0 --> 2

90 1 0.400 0 ==> 1

91 1 0.250 1 --> 0

92 1 0.250 1 --> 0

97 1 0.250 0 ==> 1

120 1 0.111 1 --> 0

122 1 0.111 1 --> 0

node_53 --> node_54 1 1 0.188 0 --> 2

31 1 0.182 0 --> 1

33 1 1.000 0 ==> 1

35 1 0.500 0 ==> 1

41 1 0.333 1 ==> 0

50 1 0.429 1 --> 2

node_54 --> node_55 10 1 0.250 0 ==> 1

31 1 0.182 1 --> 2

37 1 0.143 0 --> 1

39 1 0.167 1 --> 0

40 1 0.200 0 --> 1

73 1 0.286 1 --> 2

74 1 0.500 0 ==> 1

76 1 0.200 0 ==> 1

80 1 0.500 0 --> 1

106 1 0.111 0 --> 1

133 1 0.143 0 --> 1

134 1 0.143 0 --> 1

node_55 --> node_56 16 1 0.125 1 ==> 0

23 1 0.125 0 --> 1

67 1 0.333 0 ==> 2

94 1 0.200 0 ==> 2

95 1 0.182 0 ==> 2

98 1 0.125 1 --> 0

100 1 0.125 0 --> 1

118 1 0.400 0 --> 1

125 1 0.143 0 --> 1

128 1 0.250 0 ==> 1

135 1 0.125 1 --> 0

node_56 --> node_57 12 1 0.333 0 --> 1

40 1 0.200 1 --> 0

46 1 0.091 0 --> 1

50 1 0.429 2 --> 1

99 1 0.125 0 --> 1

117 1 0.143 0 --> 1

138 1 0.167 0 ==> 1

139 1 0.167 0 ==> 1

145 1 0.200 0 --> 1

147 1 0.400 0 --> 2

152 1 0.500 0 --> 1

node_57 --> node_60 23 1 0.125 1 --> 0

72 1 0.333 0 --> 1

73 1 0.286 2 --> 1

100 1 0.125 1 --> 0

109 1 0.250 2 --> 1

118 1 0.400 1 --> 0

node_60 --> node_68 12 1 0.333 1 --> 0

31 1 0.182 2 --> 1

39 1 0.167 0 --> 1

46 1 0.091 1 --> 0

50 1 0.429 1 --> 2

96 1 0.333 0 --> 1

99 1 0.125 1 --> 0

106 1 0.111 1 --> 0

113 1 0.222 1 ==> 0

147 1 0.400 2 --> 0

node_68 --> node_70 4 1 0.091 0 --> 1

125 1 0.143 1 --> 0

152 1 0.500 1 --> 0

node_70 --> node_71 1 1 0.188 2 --> 0

3 1 0.125 0 ==> 1

6 1 0.333 0 --> 1

19 1 0.125 1 ==> 0

23 1 0.125 0 ==> 1

45 1 0.143 0 --> 1

47 1 0.091 0 --> 1

67 1 0.333 2 ==> 1

78 1 0.333 0 ==> 1

105 1 0.111 0 ==> 1

113 1 0.222 0 --> 2

118 1 0.400 0 ==> 1

133 1 0.143 1 --> 0

134 1 0.143 1 --> 0

139 1 0.167 1 --> 0

node_71 --> node_74 95 1 0.182 2 --> 0

103 1 0.143 0 --> 1

138 1 0.167 1 ==> 0

node_74 --> node_75 42 1 0.250 0 --> 1

63 1 0.500 0 --> 1

94 1 0.200 2 ==> 0

136 1 0.333 0 --> 1

node_75 --> YPM 8287 41 1 0.333 0 ==> 1

47 1 0.091 1 --> 0

105 1 0.111 1 ==> 0

node_75 --> Enyaliodes oshaughnessyi 9 1 0.333 0 ==> 1

26 1 0.500 0 ==> 1

40 1 0.200 0 ==> 1

62 1 0.143 0 ==> 1

86 1 0.167 0 ==> 1

88 1 0.222 1 ==> 2

91 1 0.250 0 ==> 1

100 1 0.125 0 ==> 1

102 1 0.333 0 ==> 1

node_74 --> node_73 1 1 0.188 0 --> 1

6 1 0.333 1 --> 0

31 1 0.182 1 --> 2

45 1 0.143 1 --> 0

64 1 0.250 0 --> 1

95 1 0.182 0 --> 1

98 1 0.125 0 ==> 1

99 1 0.125 0 --> 1

113 1 0.222 2 --> 0

134 1 0.143 0 --> 1

139 1 0.167 0 --> 1

143 1 0.500 0 ==> 1

node_73 --> Pristidactylus torquatus 4 1 0.091 1 ==> 0

22 1 0.143 1 ==> 0

37 1 0.143 1 ==> 0

46 1 0.091 0 ==> 1

51 1 0.222 0 ==> 1

61 1 0.125 0 ==> 1

82 1 0.200 0 ==> 1

117 1 0.143 1 ==> 0

120 1 0.111 0 ==> 1

126 1 0.167 0 ==> 1

141 1 0.333 0 ==> 1

147 1 0.400 0 ==> 2

node_73 --> node_72 1 1 0.188 1 --> 2

2 1 0.250 0 --> 1

16 1 0.125 0 ==> 1

18 1 0.200 0 --> 1

23 1 0.125 1 ==> 0

34 1 0.400 0 --> 2

47 1 0.091 1 --> 0

83 1 0.333 2 ==> 0

103 1 0.143 1 --> 0

104 1 0.200 0 --> 1

106 1 0.111 0 ==> 1

113 1 0.222 0 --> 1

118 1 0.400 1 ==> 2

125 1 0.143 0 ==> 1

131 1 1.000 0 ==> 1

133 1 0.143 0 ==> 1

144 1 0.500 0 ==> 1

node_72 --> Oplurus quadrimaculatus 36 1 0.250 0 ==> 1

39 1 0.167 1 ==> 0

62 1 0.143 0 ==> 1

94 1 0.200 2 ==> 1

109 1 0.250 1 ==> 2

127 1 0.333 0 ==> 1

135 1 0.125 0 ==> 1

137 1 1.000 0 ==> 1

node_72 --> Chalarodon madagascariensis 3 1 0.125 1 ==> 0

19 1 0.125 0 ==> 1

27 1 0.200 0 ==> 1

64 1 0.250 1 --> 0

99 1 0.125 1 --> 0

122 1 0.111 0 ==> 1

128 1 0.250 1 ==> 0

node_71 --> Polychrus acutirostris 7 1 0.200 0 ==> 1

9 1 0.333 0 ==> 1

11 1 0.200 0 ==> 1

28 1 1.000 0 ==> 1

29 1 0.250 0 ==> 1

32 1 0.667 0 ==> 1

37 1 0.143 1 ==> 0

40 1 0.200 0 ==> 1

46 1 0.091 0 ==> 1

51 1 0.222 0 ==> 1

53 1 0.111 0 ==> 1

59 1 0.167 0 ==> 1

60 1 0.333 0 ==> 1

61 1 0.125 0 ==> 1

62 1 0.143 0 ==> 1

66 1 0.143 0 ==> 1

72 1 0.333 1 ==> 0

73 1 0.286 1 ==> 2

76 1 0.200 1 ==> 0

82 1 0.200 0 ==> 1

86 1 0.167 0 ==> 1

87 1 0.250 0 ==> 1

92 1 0.250 0 ==> 1

108 1 0.200 0 ==> 1

109 1 0.250 1 ==> 2

111 1 0.333 0 ==> 2

114 1 0.286 0 ==> 2

115 1 0.125 0 ==> 1

121 1 0.250 0 ==> 1

122 1 0.111 0 ==> 1

124 1 0.200 0 ==> 1

128 1 0.250 1 ==> 0

129 1 0.250 1 ==> 0

132 1 0.500 0 ==> 1

147 1 0.400 0 ==> 2

node_70 --> node_69 31 1 0.182 1 --> 2

39 1 0.167 1 --> 0

51 1 0.222 0 ==> 2

68 1 0.250 1 --> 0

96 1 0.333 1 --> 0

100 1 0.125 0 ==> 1

109 1 0.250 1 --> 0

117 1 0.143 1 --> 0

135 1 0.125 0 --> 1

140 1 1.000 0 ==> 1

node_69 --> Phymaturus palluma 1 1 0.188 2 --> 3

2 1 0.250 0 ==> 1

59 1 0.167 0 ==> 1

86 1 0.167 0 ==> 1

88 1 0.222 1 ==> 2

94 1 0.200 2 ==> 0

95 1 0.182 2 ==> 0

112 1 0.667 1 ==> 0

123 1 0.143 1 ==> 0

124 1 0.200 0 ==> 1

node_69 --> Liolaemus pictus 4 1 0.091 1 --> 0

16 1 0.125 0 ==> 1

36 1 0.250 0 ==> 1

87 1 0.250 0 ==> 1

98 1 0.125 0 ==> 1

99 1 0.125 0 ==> 1

120 1 0.111 0 ==> 1

122 1 0.111 0 ==> 1

126 1 0.167 0 ==> 1

node_68 --> node_67 1 1 0.188 2 --> 1

18 1 0.200 0 ==> 1

37 1 0.143 1 --> 0

95 1 0.182 2 --> 1

138 1 0.167 1 --> 0

151 1 0.500 0 ==> 1

node_67 --> node_65 68 1 0.250 1 ==> 0

88 1 0.222 1 --> 2

node_65 --> node_64 3 1 0.125 0 ==> 1

11 1 0.200 0 --> 1

17 1 0.500 0 --> 1

19 1 0.125 1 ==> 0

23 1 0.125 0 ==> 1

27 1 0.200 0 ==> 1

30 1 0.333 0 ==> 1

45 1 0.143 0 ==> 1

46 1 0.091 0 ==> 1

51 1 0.222 0 ==> 1

52 1 0.250 0 ==> 2

61 1 0.125 0 --> 1

67 1 0.333 2 --> 1

72 1 0.333 1 --> 2

76 1 0.200 1 --> 0

105 1 0.111 0 ==> 1

109 1 0.250 1 ==> 2

111 1 0.333 0 --> 2

115 1 0.125 0 --> 1

119 1 0.143 0 ==> 1

125 1 0.143 1 --> 0

149 1 0.500 0 --> 1

node_64 --> node_62 1 1 0.188 1 --> 0

4 1 0.091 0 ==> 1

36 1 0.250 0 ==> 1

40 1 0.200 0 ==> 1

50 1 0.429 2 ==> 3

55 1 1.000 0 ==> 1

62 1 0.143 0 ==> 1

103 1 0.143 0 --> 1

node_62 --> Basiliscus basiliscus 2 1 0.250 0 ==> 1

65 1 0.500 0 ==> 1

74 1 0.500 1 ==> 0

112 1 0.667 1 ==> 2

115 1 0.125 1 --> 0

142 1 1.000 0 ==> 1

node_62 --> node_61 11 1 0.200 1 --> 0

53 1 0.111 0 ==> 1

56 1 1.000 0 --> 1

59 1 0.167 0 ==> 1

60 1 0.333 0 ==> 1

81 1 0.500 0 ==> 1

114 1 0.286 0 ==> 1

133 1 0.143 1 --> 0

134 1 0.143 1 --> 0

node_61 --> Corytophanes cristatus 21 1 0.500 0 ==> 1

27 1 0.200 1 ==> 0

29 1 0.250 0 ==> 1

34 1 0.400 0 ==> 2

39 1 0.167 1 ==> 2

42 1 0.250 0 ==> 1

57 1 1.000 0 ==> 1

85 1 0.500 0 ==> 1

86 1 0.167 0 ==> 1

95 1 0.182 1 ==> 2

97 1 0.250 1 ==> 0

105 1 0.111 1 ==> 0

108 1 0.200 0 ==> 1

121 1 0.250 0 ==> 1

node_61 --> Laemanctus longpipes 3 1 0.125 1 ==> 0

22 1 0.143 1 ==> 0

47 1 0.091 0 ==> 1

88 1 0.222 2 --> 1

122 1 0.111 0 ==> 1

node_64 --> node_63 18 1 0.200 1 --> 0

88 1 0.222 2 --> 1

94 1 0.200 2 ==> 1

node_63 --> Geiseltaliellus maarius 34 1 0.400 0 ==> 1

100 1 0.125 0 ==> 1

101 1 0.500 0 ==> 1

node_65 --> Leiocephalus personatus 1 1 0.188 1 --> 2

5 1 0.500 0 ==> 1

7 1 0.200 0 ==> 1

31 1 0.182 1 --> 2

37 1 0.143 0 --> 1

39 1 0.167 1 --> 0

66 1 0.143 0 ==> 1

73 1 0.286 1 ==> 0

83 1 0.333 2 ==> 1

95 1 0.182 1 --> 2

96 1 0.333 1 --> 0

100 1 0.125 0 ==> 1

104 1 0.200 0 ==> 1

106 1 0.111 0 --> 1

108 1 0.200 0 ==> 1

113 1 0.222 0 ==> 1

120 1 0.111 0 ==> 1

122 1 0.111 0 ==> 1

127 1 0.333 0 ==> 1

138 1 0.167 0 --> 1

145 1 0.200 1 ==> 0

node_67 --> node_66 16 1 0.125 0 ==> 1

22 1 0.143 1 ==> 0

34 1 0.400 0 ==> 2

38 1 0.250 0 ==> 1

64 1 0.250 0 ==> 1

83 1 0.333 2 --> 0

94 1 0.200 2 --> 0

103 1 0.143 0 ==> 1

111 1 0.333 0 ==> 1

126 1 0.167 0 ==> 1

133 1 0.143 1 --> 0

134 1 0.143 1 --> 0

135 1 0.125 0 --> 1

139 1 0.167 1 ==> 0

node_66 --> Crotaphytus collaris 17 1 0.500 0 ==> 1

47 1 0.091 0 ==> 1

89 1 0.333 0 ==> 1

92 1 0.250 0 ==> 1

95 1 0.182 1 --> 0

105 1 0.111 0 ==> 1

115 1 0.125 0 ==> 1

117 1 0.143 1 --> 0

node_66 --> Gambelia wislizenii 61 1 0.125 0 ==> 1

94 1 0.200 0 --> 1

106 1 0.111 0 --> 1

119 1 0.143 0 ==> 1

node_60 --> node_59 16 1 0.125 0 ==> 1

79 1 0.400 2 --> 1

80 1 0.500 1 --> 0

104 1 0.200 0 ==> 1

117 1 0.143 1 --> 0

120 1 0.111 0 --> 1

126 1 0.167 0 ==> 1

127 1 0.333 0 --> 1

130 1 0.500 0 --> 1

145 1 0.200 1 --> 0

node_59 --> Stenocercus scapularis 1 1 0.188 2 ==> 1

10 1 0.250 1 ==> 0

19 1 0.125 1 ==> 0

37 1 0.143 1 ==> 0

39 1 0.167 0 --> 1

45 1 0.143 0 ==> 1

48 1 0.333 0 ==> 1

115 1 0.125 0 ==> 1

117 1 0.143 0 --> 1

122 1 0.111 0 ==> 1

147 1 0.400 2 ==> 1

node_59 --> Microlophus occipitalis 86 1 0.167 0 ==> 1

node_59 --> node_58 7 1 0.200 0 ==> 1

46 1 0.091 1 --> 0

113 1 0.222 1 --> 2

120 1 0.111 1 --> 0

123 1 0.143 1 ==> 0

node_58 --> Tropidurus torquatus 1 1 0.188 2 ==> 3

88 1 0.222 1 ==> 2

node_58 --> Plica umbra 3 1 0.125 0 ==> 1

4 1 0.091 0 ==> 1

10 1 0.250 1 ==> 0

42 1 0.250 0 ==> 1

66 1 0.143 0 ==> 1

98 1 0.125 0 ==> 1

105 1 0.111 0 ==> 1

119 1 0.143 0 ==> 1

node_57 --> node_40 1 1 0.188 2 --> 0

11 1 0.200 0 ==> 1

13 1 1.000 1 ==> 0

19 1 0.125 1 ==> 0

22 1 0.143 1 ==> 0

29 1 0.250 0 ==> 1

36 1 0.250 0 ==> 1

37 1 0.143 1 ==> 0

45 1 0.143 0 ==> 1

51 1 0.222 0 ==> 1

52 1 0.250 0 ==> 2

65 1 0.500 0 ==> 2

75 1 1.000 0 --> 1

76 1 0.200 1 --> 0

77 1 0.500 0 --> 1

78 1 0.333 0 --> 1

82 1 0.200 0 ==> 1

85 1 0.500 0 ==> 1

87 1 0.250 0 ==> 1

89 1 0.333 0 ==> 1

98 1 0.125 0 --> 1

108 1 0.200 0 ==> 1

114 1 0.286 0 ==> 1

116 1 0.400 0 ==> 2

121 1 0.250 0 ==> 1

125 1 0.143 1 --> 0

132 1 0.500 0 ==> 1

133 1 0.143 1 --> 0

134 1 0.143 1 --> 0

135 1 0.125 0 --> 1

141 1 0.333 0 ==> 1

143 1 0.500 0 ==> 1

node_40 --> Anolis ricordi 3 1 0.125 0 ==> 1

4 1 0.091 0 ==> 1

30 1 0.333 0 ==> 1

31 1 0.182 2 --> 1

47 1 0.091 0 ==> 1

53 1 0.111 0 ==> 1

60 1 0.333 0 ==> 1

61 1 0.125 0 ==> 1

62 1 0.143 0 ==> 1

101 1 0.500 0 ==> 1

111 1 0.333 0 ==> 1

node_40 --> Anolis cristatellus 10 1 0.250 1 ==> 0

59 1 0.167 0 ==> 1

128 1 0.250 1 ==> 0

node_56 --> node_42 1 1 0.188 2 ==> 1

2 1 0.250 0 ==> 1

4 1 0.091 0 --> 1

18 1 0.200 0 ==> 1

24 1 1.000 0 ==> 1

39 1 0.167 0 --> 1

58 1 0.500 0 ==> 1

65 1 0.500 0 ==> 1

73 1 0.286 2 --> 0

83 1 0.333 2 --> 1

88 1 0.222 1 ==> 2

90 1 0.400 1 ==> 2

91 1 0.250 0 ==> 1

106 1 0.111 1 --> 0

111 1 0.333 0 --> 1

113 1 0.222 1 ==> 0

116 1 0.400 0 ==> 1

150 1 1.000 0 ==> 1

151 1 0.500 0 ==> 1

node_42 --> Dipsosaurus dorsalis 38 1 0.250 0 ==> 1

50 1 0.429 2 ==> 3

120 1 0.111 0 ==> 1

123 1 0.143 1 --> 0

node_42 --> node_41 3 1 0.125 0 ==> 1

31 1 0.182 2 --> 0

61 1 0.125 0 ==> 1

79 1 0.400 2 ==> 1

105 1 0.111 0 ==> 1

108 1 0.200 0 ==> 1

111 1 0.333 1 --> 2

115 1 0.125 0 ==> 1

133 1 0.143 1 --> 0

134 1 0.143 1 --> 0

node_41 --> Brachylophus fasciatus 4 1 0.091 1 --> 0

72 1 0.333 0 ==> 1

node_41 --> Iguana iguana 11 1 0.200 0 ==> 1

31 1 0.182 0 --> 1

51 1 0.222 0 ==> 1

52 1 0.250 0 ==> 2

67 1 0.333 2 ==> 1

103 1 0.143 0 ==> 1

126 1 0.167 0 ==> 1

node_55 --> node_44 53 1 0.111 0 --> 1

66 1 0.143 0 ==> 1

71 1 0.500 0 --> 1

80 1 0.500 1 --> 2

86 1 0.167 0 --> 1

109 1 0.250 2 --> 1

123 1 0.143 1 --> 0

127 1 0.333 0 ==> 1

146 1 1.000 0 ==> 1

148 1 0.500 0 ==> 1

node_44 --> Phrynosoma platyrhinos 4 1 0.091 0 ==> 1

15 1 0.333 0 ==> 1

19 1 0.125 1 ==> 0

21 1 0.500 0 ==> 1

25 1 0.333 0 ==> 1

32 1 0.667 0 ==> 2

39 1 0.167 0 --> 2

42 1 0.250 0 ==> 1

46 1 0.091 0 ==> 1

47 1 0.091 0 ==> 1

51 1 0.222 0 ==> 2

59 1 0.167 0 ==> 1

62 1 0.143 0 ==> 1

88 1 0.222 1 ==> 0

90 1 0.400 1 ==> 0

99 1 0.125 0 ==> 1

104 1 0.200 0 ==> 1

114 1 0.286 0 ==> 1

115 1 0.125 0 ==> 1

124 1 0.200 0 ==> 1

136 1 0.333 0 ==> 1

node_44 --> node_43 12 1 0.333 0 ==> 1

83 1 0.333 2 --> 1

94 1 0.200 0 ==> 1

95 1 0.182 0 ==> 1

130 1 0.500 0 ==> 1

node_43 --> Petrosaurus thalassinus 1 1 0.188 2 ==> 1

7 1 0.200 0 ==> 1

67 1 0.333 0 ==> 1

109 1 0.250 1 ==> 0

113 1 0.222 1 ==> 0

node_43 --> Sceloporus undulatus 53 1 0.111 1 --> 0

65 1 0.500 0 ==> 2

76 1 0.200 1 ==> 0

105 1 0.111 0 ==> 1

120 1 0.111 0 ==> 1

123 1 0.143 0 --> 1

125 1 0.143 0 ==> 1

138 1 0.167 0 ==> 1

145 1 0.200 0 ==> 1

node_54 --> node_48 1 1 0.188 2 --> 3

8 1 1.000 0 ==> 1

14 1 1.000 0 ==> 1

18 1 0.200 0 --> 1

32 1 0.667 0 --> 1

38 1 0.250 0 ==> 1

45 1 0.143 0 ==> 1

61 1 0.125 0 ==> 1

69 1 1.000 0 --> 1

70 1 1.000 0 --> 1

89 1 0.333 0 ==> 1

93 1 0.500 0 ==> 1

103 1 0.143 0 --> 1

115 1 0.125 0 --> 1

119 1 0.143 0 --> 1

127 1 0.333 0 --> 2

145 1 0.200 0 --> 1

149 1 0.500 0 --> 1

node_48 --> node_47 15 1 0.333 0 --> 1

34 1 0.400 0 ==> 2

66 1 0.143 0 ==> 1

97 1 0.250 1 ==> 0

99 1 0.125 0 ==> 1

102 1 0.333 0 ==> 1

node_47 --> node_46 19 1 0.125 1 ==> 0

26 1 0.500 0 ==> 1

39 1 0.167 1 --> 0

46 1 0.091 0 --> 1

82 1 0.200 0 --> 1

114 1 0.286 0 --> 1

121 1 0.250 0 --> 1

123 1 0.143 1 --> 0

135 1 0.125 1 --> 0

node_46 --> node_45 15 1 0.333 1 --> 0

20 1 0.500 0 ==> 1

22 1 0.143 1 ==> 0

84 1 1.000 0 ==> 1

90 1 0.400 1 ==> 0

114 1 0.286 1 --> 2

117 1 0.143 0 ==> 1

node_45 --> Physignathus cocincinus 16 1 0.125 1 ==> 0

62 1 0.143 0 ==> 1

68 1 0.250 1 --> 0

72 1 0.333 0 ==> 1

73 1 0.286 1 ==> 0

79 1 0.400 2 --> 0

97 1 0.250 0 ==> 1

98 1 0.125 1 ==> 0

106 1 0.111 0 ==> 1

112 1 0.667 1 ==> 2

node_45 --> Agama agama 4 1 0.091 0 ==> 1

46 1 0.091 1 --> 0

49 1 0.500 0 ==> 1

80 1 0.500 0 ==> 2

119 1 0.143 1 --> 0

124 1 0.200 0 ==> 1

135 1 0.125 0 --> 1

node_46 --> Brookesia superciliaris 5 1 0.500 0 ==> 1

9 1 0.333 0 ==> 1

23 1 0.125 0 ==> 1

25 1 0.333 0 ==> 1

29 1 0.250 0 ==> 1

31 1 0.182 1 --> 2

38 1 0.250 1 ==> 0

39 1 0.167 0 --> 2

47 1 0.091 0 ==> 1

50 1 0.429 2 ==> 3

53 1 0.111 0 --> 1

81 1 0.500 0 ==> 1

107 1 0.500 1 ==> 0

110 1 1.000 0 ==> 1

113 1 0.222 1 ==> 2

118 1 0.400 0 ==> 1

129 1 0.250 1 ==> 0

138 1 0.167 0 ==> 1

139 1 0.167 0 ==> 1

144 1 0.500 0 ==> 1

node_47 --> Leiolepis belliana 4 1 0.091 0 ==> 1

48 1 0.333 0 ==> 1

49 1 0.500 0 ==> 1

58 1 0.500 0 ==> 1

63 1 0.500 0 ==> 1

79 1 0.400 2 ==> 1

87 1 0.250 0 ==> 1

122 1 0.111 0 --> 1

148 1 0.500 0 ==> 1

node_48 --> Priscagama gobiensis 22 1 0.143 1 ==> 0

47 1 0.091 0 ==> 1

53 1 0.111 0 --> 1

90 1 0.400 1 ==> 0

105 1 0.111 0 ==> 1

node_52 --> Elgaria multicarinata 7 1 0.200 0 ==> 1

16 1 0.125 1 ==> 0

46 1 0.091 0 ==> 1

51 1 0.222 0 ==> 1

71 1 0.500 0 --> 1

78 1 0.333 0 ==> 1

100 1 0.125 0 ==> 1

116 1 0.400 0 --> 2

129 1 0.250 1 --> 0

node_51 --> Plestiodon fasciatus 1 1 0.188 0 ==> 1

6 1 0.333 0 ==> 1

20 1 0.500 0 ==> 1

30 1 0.333 0 --> 1

67 1 0.333 0 ==> 1

73 1 0.286 1 ==> 0

116 1 0.400 0 ==> 1

127 1 0.333 0 ==> 1

node_50 --> Eublepharis macularius 31 1 0.182 0 ==> 1

43 1 0.500 0 ==> 1

44 1 0.500 0 ==> 1

46 1 0.091 0 ==> 1

48 1 0.333 0 ==> 1

51 1 0.222 0 --> 2

66 1 0.143 0 --> 1

82 1 0.200 0 ==> 1

94 1 0.200 0 ==> 2

95 1 0.182 0 ==> 2

100 1 0.125 0 ==> 1

104 1 0.200 0 --> 1

114 1 0.286 0 ==> 1

125 1 0.143 0 ==> 1

126 1 0.167 0 ==> 1

node_49 --> Diphydontosaurus avonis 23 1 0.125 1 ==> 0

94 1 0.200 0 ==> 1

95 1 0.182 0 ==> 1

node_49 --> Sphenodon punctatus 1 1 0.188 0 ==> 2

3 1 0.125 0 ==> 1

25 1 0.333 0 --> 1

35 1 0.500 0 ==> 1

45 1 0.143 0 --> 1

52 1 0.250 0 ==> 2

61 1 0.125 0 --> 1

66 1 0.143 0 --> 1

77 1 0.500 0 --> 1

93 1 0.500 0 ==> 1

102 1 0.333 0 --> 1

103 1 0.143 0 --> 1

116 1 0.400 0 --> 2

124 1 0.200 0 --> 1

139 1 0.167 0 --> 1

141 1 0.333 0 --> 1

Parsimony analysis with scaffold based on Streicher et al [4]

/---------------------------- YPM 8287

|

| /------------------- Pristidactylus torquatus

| |

/-------74-------73 /--------- Oplurus quadrimaculatus

| | \--------72

| | \--------- Chalarodon madagascariensis

| |

/--------71 \---------------------------- Enyaliodes oshaughnessyi

| |

| | /------------------- Polychrus acutirostris

| | |

| \----------------41 /--------- Phymaturus palluma

| \--------40

| \--------- Liolaemus pictus

/-------70

| | /--------- Anolis ricordi

| | /--------42

| | | \--------- Anolis cristatellus

| | /-------43

| | | \------------------- Leiocephalus personatus

| | |

| \-----------------47 /--------- Stenocercus scapularis

| | /--------44

| | | \--------- Microlophus occipitalis

| \-------46

/-------69 | /--------- Tropidurus torquatus

| | \--------45

| | \--------- Plica umbra

| |

| | /------------------- Basiliscus basiliscus

| | |

| | /-------49 /--------- Corytophanes cristatus

| | | \--------48

| | | \--------- Laemanctus longpipes

| | /-------50

/--------68 | | +---------------------------- Geiseltaliellus maarius

| | | | |

| | \-----------------52 \---------------------------- Suzanniwanna patriciana

| | |

| | | /--------- Crotaphytus collaris

| | \--------------------------51

| | \--------- Gambelia wislizenii

| |

/-------67 | /------------------- Dipsosaurus dorsalis

| | | |

| | \--------------------------------------------54 /--------- Brachylophus fasciatus

| | \--------53

| | \--------- Iguana iguana

| |

| | /------------------- Phrynosoma platyrhinos

| | |

| \------------------------------------------------------56 /--------- Petrosaurus thalassinus

/--------66 \--------55

| | \--------- Sceloporus undulatus

| |

| | /--------- Physignathus cocincinus

| | /--------57

| | | \--------- Agama agama

/-------65 | /-------58

| | | /-------59 \------------------- Brookesia superciliaris

| | | | |

| | \---------------------------------------------60 \---------------------------- Leiolepis belliana

| | |

/-------64 | \------------------------------------- Priscagama gobiensis

| | |

| | \---------------------------------------------------------------------------------------------- Saichangurvel davidsoni

/--------63 |

| | \------------------------------------------------------------------------------------------------------- Elgaria multicarinata

/-------62 |

| | \---------------------------------------------------------------------------------------------------------------- Plestiodon fasciatus

| |

| \-------------------------------------------------------------------------------------------------------------------------- Eublepharis macularius

61

+----------------------------------------------------------------------------------------------------------------------------------- Diphydontosaurus avonis

|

\----------------------------------------------------------------------------------------------------------------------------------- Sphenodon punctatus

Branch Character Steps CI Change

-------------------------------------------------------------------------

node_61 --> node_62 16 1 0.125 0 ==> 1

41 1 0.333 0 --> 1

53 1 0.111 0 ==> 1

54 1 0.500 0 ==> 1

64 1 0.250 1 ==> 0

83 1 0.333 0 --> 2

91 1 0.250 0 --> 1

92 1 0.250 0 --> 1

98 1 0.125 1 --> 0

99 1 0.125 1 ==> 0

106 1 0.143 0 --> 1

119 1 0.143 0 --> 1

122 1 0.111 0 --> 1

135 1 0.125 0 --> 1

node_62 --> node_63 27 1 0.200 0 --> 1

47 1 0.091 0 --> 1

88 1 0.222 0 --> 1

120 1 0.125 0 --> 1

129 1 0.250 0 --> 1

136 1 0.333 1 ==> 0

node_63 --> node_64 39 1 0.200 0 ==> 1

59 1 0.167 1 ==> 0

68 1 0.200 0 --> 1

98 1 0.125 0 --> 1

106 1 0.143 1 --> 0

107 1 0.500 0 --> 1

119 1 0.143 1 --> 0

123 1 0.143 0 --> 1

node_64 --> node_65 18 1 0.167 0 --> 1

19 1 0.111 0 ==> 1

22 1 0.143 0 ==> 1

23 1 0.111 1 ==> 0

27 1 0.200 1 --> 0

43 1 0.500 0 ==> 1

44 1 0.500 0 ==> 1

47 1 0.091 1 --> 0

50 1 0.429 0 --> 1

53 1 0.111 1 --> 0

54 1 0.500 1 ==> 0

72 1 0.286 2 --> 0

79 1 0.400 0 --> 2

90 1 0.400 0 ==> 1

91 1 0.250 1 --> 0

92 1 0.250 1 --> 0

97 1 0.250 0 ==> 1

120 1 0.125 1 --> 0

122 1 0.111 1 --> 0

node_65 --> node_66 1 1 0.200 0 --> 1

31 1 0.200 0 ==> 1

33 1 1.000 0 ==> 1

35 1 0.500 0 ==> 1

41 1 0.333 1 ==> 0

50 1 0.429 1 --> 2

115 1 0.125 0 --> 1

node_66 --> node_67 10 1 0.250 0 ==> 1

37 1 0.167 0 --> 1

40 1 0.200 0 --> 1

74 1 0.500 0 ==> 1

76 1 0.200 0 ==> 1

80 1 0.500 0 --> 1

134 1 0.143 0 --> 1

node_67 --> node_68 16 1 0.125 1 ==> 0

23 1 0.111 0 --> 1

67 1 0.286 0 ==> 1

72 1 0.286 0 --> 1

94 1 0.182 0 ==> 2

95 1 0.200 0 ==> 2

98 1 0.125 1 ==> 0

105 1 0.100 0 --> 1

111 1 0.333 0 --> 1

113 1 0.250 1 ==> 0

125 1 0.143 0 --> 1

128 1 0.250 0 ==> 1

135 1 0.125 1 ==> 0

151 1 0.500 0 --> 1

node_68 --> node_69 40 1 0.200 1 --> 0

117 1 0.143 0 ==> 1

139 1 0.167 0 --> 1

145 1 0.200 0 --> 1

152 1 0.500 0 --> 1

node_69 --> node_70 1 1 0.200 1 --> 2

18 1 0.167 1 --> 0

78 1 0.250 0 --> 1

111 1 0.333 1 --> 0

115 1 0.125 1 --> 0

138 1 0.250 0 --> 1

151 1 0.500 1 --> 0

node_70 --> node_71 3 1 0.111 0 --> 1

4 1 0.091 0 ==> 1

19 1 0.111 1 --> 0

118 1 0.333 0 --> 1

125 1 0.143 1 --> 0

152 1 0.500 1 --> 0

node_71 --> node_74 94 1 0.182 2 --> 0

95 1 0.200 2 ==> 0

96 1 0.500 0 ==> 1

103 1 0.143 0 ==> 1

138 1 0.250 1 --> 0

node_74 --> YPM 8287 41 1 0.333 0 ==> 1

105 1 0.100 1 --> 0

node_74 --> node_73 31 1 0.200 1 ==> 2

64 1 0.250 0 --> 1

94 1 0.182 0 --> 2

95 1 0.200 0 ==> 1

98 1 0.125 0 ==> 1

99 1 0.125 0 --> 1

143 1 0.500 0 ==> 1

node_73 --> Pristidactylus torquatus 1 1 0.200 2 ==> 1

4 1 0.091 1 ==> 0

22 1 0.143 1 ==> 0

37 1 0.167 1 ==> 0

46 1 0.100 0 ==> 1

47 1 0.091 0 ==> 1

51 1 0.222 0 ==> 1

61 1 0.125 0 ==> 1

82 1 0.200 0 ==> 1

117 1 0.143 1 ==> 0

120 1 0.125 0 ==> 1

126 1 0.167 0 ==> 1

141 1 0.333 0 ==> 1

147 1 0.400 0 ==> 2

node_73 --> node_72 2 1 0.250 0 --> 1

16 1 0.125 0 ==> 1

18 1 0.167 0 --> 1

23 1 0.111 1 ==> 0

34 1 0.400 0 --> 2

83 1 0.333 2 ==> 0

103 1 0.143 1 ==> 0

104 1 0.200 0 --> 1

106 1 0.143 0 ==> 1

113 1 0.250 0 --> 1

118 1 0.333 1 --> 2

125 1 0.143 0 --> 1

131 1 1.000 0 ==> 1

133 1 0.143 0 ==> 1

144 1 0.500 0 ==> 1

node_72 --> Oplurus quadrimaculatus 36 1 0.250 0 ==> 1

39 1 0.200 1 ==> 0

62 1 0.143 0 ==> 1

94 1 0.182 2 ==> 1

109 1 0.250 1 ==> 2

127 1 0.333 0 ==> 1

135 1 0.125 0 ==> 1

137 1 1.000 0 ==> 1

node_72 --> Chalarodon madagascariensis 3 1 0.111 1 ==> 0

19 1 0.111 0 ==> 1

27 1 0.200 0 ==> 1

64 1 0.250 1 --> 0

99 1 0.125 1 --> 0

122 1 0.111 0 ==> 1

128 1 0.250 1 ==> 0

node_74 --> Enyaliodes oshaughnessyi 1 1 0.200 2 ==> 0

6 1 0.333 0 ==> 1

9 1 0.333 0 ==> 1

26 1 0.500 0 ==> 1

40 1 0.200 0 ==> 1

42 1 0.250 0 ==> 1

45 1 0.143 0 ==> 1

47 1 0.091 0 ==> 1

62 1 0.143 0 ==> 1

63 1 0.500 0 ==> 1

86 1 0.167 0 ==> 1

88 1 0.222 1 ==> 2

91 1 0.250 0 ==> 1

100 1 0.143 0 ==> 1

102 1 0.333 0 ==> 1

113 1 0.250 0 ==> 2

134 1 0.143 1 --> 0

136 1 0.333 0 ==> 1

139 1 0.167 1 --> 0

node_71 --> node_41 11 1 0.200 0 --> 1

28 1 1.000 0 --> 1

32 1 0.667 0 --> 1

40 1 0.200 0 --> 1

51 1 0.222 0 --> 1

59 1 0.167 0 --> 1

86 1 0.167 0 --> 1

87 1 0.250 0 --> 1

109 1 0.250 1 --> 0

122 1 0.111 0 --> 1

124 1 0.200 0 --> 1

128 1 0.250 1 --> 0

135 1 0.125 0 --> 1

node_41 --> Polychrus acutirostris 1 1 0.200 2 ==> 0

6 1 0.333 0 ==> 1

7 1 0.200 0 ==> 1

9 1 0.333 0 ==> 1

29 1 0.250 0 ==> 1

37 1 0.167 1 ==> 0

45 1 0.143 0 ==> 1

46 1 0.100 0 ==> 1

47 1 0.091 0 ==> 1

53 1 0.111 0 ==> 1

60 1 0.333 0 ==> 1

61 1 0.125 0 ==> 1

62 1 0.143 0 ==> 1

66 1 0.143 0 ==> 1

72 1 0.286 1 ==> 0

73 1 0.286 1 ==> 2

76 1 0.200 1 ==> 0

82 1 0.200 0 ==> 1

92 1 0.250 0 ==> 1

108 1 0.250 0 ==> 1

109 1 0.250 0 --> 2

111 1 0.333 0 ==> 2

113 1 0.250 0 ==> 2

114 1 0.286 0 ==> 2

115 1 0.125 0 ==> 1

121 1 0.250 0 ==> 1

129 1 0.250 1 ==> 0

132 1 0.500 0 ==> 1

134 1 0.143 1 --> 0

139 1 0.167 1 --> 0

147 1 0.400 0 ==> 2

node_41 --> node_40 3 1 0.111 1 --> 0

19 1 0.111 0 --> 1

23 1 0.111 1 --> 0

31 1 0.200 1 ==> 2

39 1 0.200 1 ==> 0

51 1 0.222 1 --> 2

67 1 0.286 1 --> 2

68 1 0.200 1 --> 0

78 1 0.250 1 --> 0

100 1 0.143 0 ==> 1

105 1 0.100 1 --> 0

117 1 0.143 1 ==> 0

118 1 0.333 1 --> 0

133 1 0.143 0 --> 1

140 1 1.000 0 ==> 1

node_40 --> Phymaturus palluma 1 1 0.200 2 ==> 3

2 1 0.250 0 ==> 1

87 1 0.250 1 --> 0

88 1 0.222 1 ==> 2

94 1 0.182 2 ==> 0

95 1 0.200 2 ==> 0

112 1 0.667 1 ==> 0

122 1 0.111 1 --> 0

123 1 0.143 1 ==> 0

node_40 --> Liolaemus pictus 4 1 0.091 1 ==> 0

16 1 0.125 0 ==> 1

36 1 0.250 0 ==> 1

59 1 0.167 1 --> 0

86 1 0.167 1 --> 0

98 1 0.125 0 ==> 1

99 1 0.125 0 ==> 1

120 1 0.125 0 ==> 1

124 1 0.200 1 --> 0

126 1 0.167 0 ==> 1

node_70 --> node_47 12 1 0.333 0 --> 1

23 1 0.111 1 --> 0

31 1 0.200 1 ==> 2

39 1 0.200 1 ==> 0

50 1 0.429 2 --> 1

67 1 0.286 1 --> 2

73 1 0.286 1 --> 0

99 1 0.125 0 --> 1

104 1 0.200 0 --> 1

105 1 0.100 1 --> 0

106 1 0.143 0 ==> 1

113 1 0.250 0 ==> 1

127 1 0.333 0 --> 1

133 1 0.143 0 --> 1

145 1 0.200 1 --> 0

147 1 0.400 0 --> 2

node_47 --> node_43 13 1 1.000 1 --> 0

100 1 0.143 0 ==> 1

108 1 0.250 0 ==> 1

node_43 --> node_42 1 1 0.200 2 ==> 0

11 1 0.200 0 ==> 1

19 1 0.111 1 ==> 0

22 1 0.143 1 ==> 0

23 1 0.111 0 --> 1

29 1 0.250 0 ==> 1

36 1 0.250 0 ==> 1

37 1 0.167 1 ==> 0

45 1 0.143 0 ==> 1

46 1 0.100 0 ==> 1

51 1 0.222 0 ==> 1

52 1 0.250 0 ==> 2

65 1 0.500 0 ==> 2

72 1 0.286 1 --> 0

73 1 0.286 0 --> 2

75 1 1.000 0 --> 1

76 1 0.200 1 --> 0

77 1 0.500 0 --> 1

82 1 0.200 0 ==> 1

85 1 0.500 0 ==> 1

87 1 0.250 0 ==> 1

89 1 0.333 0 ==> 1

98 1 0.125 0 ==> 1

104 1 0.200 1 --> 0

109 1 0.250 1 ==> 2

114 1 0.286 0 ==> 1

116 1 0.400 0 ==> 2

118 1 0.333 0 ==> 1

121 1 0.250 0 ==> 1

125 1 0.143 1 --> 0

127 1 0.333 1 --> 0

132 1 0.500 0 ==> 1

133 1 0.143 1 --> 0

134 1 0.143 1 --> 0

135 1 0.125 0 --> 1

141 1 0.333 0 ==> 1

143 1 0.500 0 ==> 1

145 1 0.200 0 --> 1

node_42 --> Anolis ricordi 3 1 0.111 0 ==> 1

4 1 0.091 0 ==> 1

30 1 0.333 0 ==> 1

31 1 0.200 2 ==> 1

47 1 0.091 0 ==> 1

53 1 0.111 0 ==> 1

60 1 0.333 0 ==> 1

61 1 0.125 0 ==> 1

62 1 0.143 0 ==> 1

101 1 0.500 0 ==> 1

111 1 0.333 0 ==> 1

node_42 --> Anolis cristatellus 10 1 0.250 1 ==> 0

59 1 0.167 0 ==> 1

128 1 0.250 1 ==> 0

node_43 --> Leiocephalus personatus 5 1 0.500 0 ==> 1

7 1 0.200 0 ==> 1

12 1 0.333 1 --> 0

18 1 0.167 0 ==> 1

50 1 0.429 1 --> {23}

66 1 0.143 0 ==> 1

68 1 0.200 1 ==> 0

78 1 0.250 1 --> 0

83 1 0.333 2 ==> 1

88 1 0.222 1 ==> 2

99 1 0.125 1 --> 0

120 1 0.125 0 ==> 1

122 1 0.111 0 ==> 1

147 1 0.400 2 --> 0

node_47 --> node_46 16 1 0.125 0 ==> 1

79 1 0.400 2 --> 1

80 1 0.500 1 --> 0

117 1 0.143 1 --> 0

126 1 0.167 0 ==> 1

130 1 0.500 0 --> 1

node_46 --> node_44 46 1 0.100 0 ==> 1

120 1 0.125 0 ==> 1

node_44 --> Stenocercus scapularis 1 1 0.200 2 ==> 1

10 1 0.250 1 ==> 0

19 1 0.111 1 ==> 0

37 1 0.167 1 ==> 0

39 1 0.200 0 ==> 1

45 1 0.143 0 ==> 1

48 1 0.333 0 ==> 1

115 1 0.125 0 ==> 1

117 1 0.143 0 --> 1

122 1 0.111 0 ==> 1

147 1 0.400 2 ==> 1

node_44 --> Microlophus occipitalis 86 1 0.167 0 ==> 1

node_46 --> node_45 7 1 0.200 0 ==> 1

113 1 0.250 1 --> 2

123 1 0.143 1 ==> 0

node_45 --> Tropidurus torquatus 1 1 0.200 2 ==> 3

88 1 0.222 1 ==> 2

node_45 --> Plica umbra 3 1 0.111 0 ==> 1

4 1 0.091 0 ==> 1

10 1 0.250 1 ==> 0

42 1 0.250 0 ==> 1

66 1 0.143 0 ==> 1

98 1 0.125 0 ==> 1

105 1 0.100 0 ==> 1

119 1 0.143 0 ==> 1

node_69 --> node_52 17 1 0.500 0 --> 1

37 1 0.167 1 ==> 0

61 1 0.125 0 --> 1

94 1 0.182 2 ==> 1

95 1 0.200 2 ==> 1

96 1 0.500 0 ==> 1

103 1 0.143 0 --> 1

119 1 0.143 0 --> 1

134 1 0.143 1 --> 0

node_52 --> node_50 3 1 0.111 0 ==> 1

11 1 0.200 0 --> 1

19 1 0.111 1 ==> 0

27 1 0.200 0 ==> 1

30 1 0.333 0 ==> 1

45 1 0.143 0 ==> 1

46 1 0.100 0 ==> 1

51 1 0.222 0 ==> 1

52 1 0.250 0 ==> 2

68 1 0.200 1 --> 0

72 1 0.286 1 --> 2

76 1 0.200 1 --> 0

109 1 0.250 1 ==> 2

111 1 0.333 1 --> 2

125 1 0.143 1 --> 0

149 1 0.500 0 --> 1

node_50 --> node_49 1 1 0.200 1 ==> 0

4 1 0.091 0 ==> 1

36 1 0.250 0 ==> 1

40 1 0.200 0 ==> 1

50 1 0.429 2 ==> 3

55 1 1.000 0 ==> 1

62 1 0.143 0 ==> 1

88 1 0.222 1 --> 2

94 1 0.182 1 ==> 2

node_49 --> Basiliscus basiliscus 2 1 0.250 0 ==> 1

65 1 0.500 0 ==> 1

74 1 0.500 1 ==> 0

112 1 0.667 1 ==> 2

115 1 0.125 1 --> 0

133 1 0.143 0 ==> 1

134 1 0.143 0 --> 1

142 1 1.000 0 ==> 1

node_49 --> node_48 11 1 0.200 1 --> 0

53 1 0.111 0 ==> 1

56 1 1.000 0 --> 1

59 1 0.167 0 ==> 1

60 1 0.333 0 ==> 1

81 1 0.500 0 ==> 1

114 1 0.286 0 ==> 1

node_48 --> Corytophanes cristatus 21 1 0.500 0 ==> 1

27 1 0.200 1 ==> 0

29 1 0.250 0 ==> 1

34 1 0.400 0 ==> 2

39 1 0.200 1 ==> 2

42 1 0.250 0 ==> 1

57 1 1.000 0 ==> 1

85 1 0.500 0 ==> 1

86 1 0.167 0 ==> 1

95 1 0.200 1 ==> 2

97 1 0.250 1 ==> 0

105 1 0.100 1 ==> 0

108 1 0.250 0 ==> 1

121 1 0.250 0 ==> 1

node_48 --> Laemanctus longpipes 3 1 0.111 1 ==> 0

22 1 0.143 1 ==> 0

47 1 0.091 0 ==> 1

88 1 0.222 2 --> 1

122 1 0.111 0 ==> 1

node_50 --> Geiseltaliellus maarius 34 1 0.400 0 ==> 1

100 1 0.143 0 ==> 1

101 1 0.500 0 ==> 1

103 1 0.143 1 --> 0

node_50 --> Suzanniwanna patriciana 18 1 0.167 1 --> 0

node_52 --> node_51 16 1 0.125 0 ==> 1

22 1 0.143 1 ==> 0

23 1 0.111 1 --> 0

34 1 0.400 0 ==> 2

38 1 0.250 0 ==> 1

64 1 0.250 0 ==> 1

67 1 0.286 1 --> 2

83 1 0.333 2 --> 0

126 1 0.167 0 ==> 1

135 1 0.125 0 ==> 1

139 1 0.167 1 --> 0

node_51 --> Crotaphytus collaris 47 1 0.091 0 ==> 1

61 1 0.125 1 --> 0

89 1 0.333 0 ==> 1

92 1 0.250 0 ==> 1

94 1 0.182 1 ==> 0

95 1 0.200 1 ==> 0

117 1 0.143 1 ==> 0

119 1 0.143 1 --> 0

node_51 --> Gambelia wislizenii 17 1 0.500 1 --> 0

105 1 0.100 1 --> 0

106 1 0.143 0 ==> 1

115 1 0.125 1 --> 0

node_68 --> node_54 2 1 0.250 0 ==> 1

4 1 0.091 0 --> 1

24 1 1.000 0 ==> 1

58 1 0.500 0 ==> 1

65 1 0.500 0 ==> 1

73 1 0.286 1 --> 0

83 1 0.333 2 --> 1

88 1 0.222 1 ==> 2

90 1 0.400 1 ==> 2

91 1 0.250 0 ==> 1

100 1 0.143 0 ==> 1

109 1 0.250 1 ==> 2

116 1 0.400 0 ==> 1

118 1 0.333 0 --> 1

150 1 1.000 0 ==> 1

node_54 --> Dipsosaurus dorsalis 31 1 0.200 1 ==> 2

38 1 0.250 0 ==> 1

50 1 0.429 2 ==> 3

105 1 0.100 1 --> 0

115 1 0.125 1 --> 0

120 1 0.125 0 ==> 1

123 1 0.143 1 --> 0

133 1 0.143 0 ==> 1

node_54 --> node_53 3 1 0.111 0 ==> 1

61 1 0.125 0 ==> 1

79 1 0.400 2 ==> 1

108 1 0.250 0 ==> 1

111 1 0.333 1 --> 2

134 1 0.143 1 --> 0

node_53 --> Brachylophus fasciatus 4 1 0.091 1 --> 0

31 1 0.200 1 ==> 0

67 1 0.286 1 --> 2

node_53 --> Iguana iguana 11 1 0.200 0 ==> 1

51 1 0.222 0 ==> 1

52 1 0.250 0 ==> 2

72 1 0.286 1 --> 0

103 1 0.143 0 ==> 1

126 1 0.167 0 ==> 1

node_67 --> node_56 1 1 0.200 1 --> 2

18 1 0.167 1 --> 0

31 1 0.200 1 ==> 2

39 1 0.200 1 --> 0

53 1 0.111 0 --> 1

66 1 0.143 0 ==> 1

71 1 0.500 0 --> 1

73 1 0.286 1 ==> 2

80 1 0.500 1 --> 2

86 1 0.167 0 --> 1

106 1 0.143 0 ==> 1

123 1 0.143 1 --> 0

127 1 0.333 0 ==> 1

133 1 0.143 0 ==> 1

146 1 1.000 0 ==> 1

148 1 0.500 0 ==> 1

node_56 --> Phrynosoma platyrhinos 4 1 0.091 0 ==> 1

15 1 0.333 0 ==> 1

19 1 0.111 1 ==> 0

21 1 0.500 0 ==> 1

25 1 0.333 0 ==> 1

32 1 0.667 0 ==> 2

39 1 0.200 0 --> 2

42 1 0.250 0 ==> 1

46 1 0.100 0 ==> 1

47 1 0.091 0 ==> 1

51 1 0.222 0 ==> 2

59 1 0.167 0 ==> 1

62 1 0.143 0 ==> 1

88 1 0.222 1 ==> 0

90 1 0.400 1 ==> 0

99 1 0.125 0 ==> 1

104 1 0.200 0 ==> 1

114 1 0.286 0 ==> 1

124 1 0.200 0 ==> 1

136 1 0.333 0 ==> 1

node_56 --> node_55 12 1 0.333 0 ==> 1

83 1 0.333 2 --> 1

94 1 0.182 0 ==> 1

95 1 0.200 0 ==> 1

115 1 0.125 1 --> 0

130 1 0.500 0 ==> 1

node_55 --> Petrosaurus thalassinus 1 1 0.200 2 --> 1

7 1 0.200 0 ==> 1

67 1 0.286 0 ==> 1

109 1 0.250 1 ==> 0

113 1 0.250 1 ==> 0

node_55 --> Sceloporus undulatus 53 1 0.111 1 --> 0

65 1 0.500 0 ==> 2

76 1 0.200 1 ==> 0

105 1 0.100 0 ==> 1

120 1 0.125 0 ==> 1

123 1 0.143 0 --> 1

125 1 0.143 0 ==> 1

138 1 0.250 0 ==> 1

145 1 0.200 0 ==> 1

node_66 --> node_60 1 1 0.200 1 --> 3

8 1 1.000 0 ==> 1

14 1 1.000 0 ==> 1

32 1 0.667 0 --> 1

38 1 0.250 0 ==> 1

45 1 0.143 0 ==> 1

61 1 0.125 0 ==> 1

69 1 1.000 0 --> 1

70 1 1.000 0 --> 1

89 1 0.333 0 ==> 1

93 1 0.500 0 ==> 1

103 1 0.143 0 --> 1

109 1 0.250 1 --> 2

119 1 0.143 0 --> 1

127 1 0.333 0 --> 2

145 1 0.200 0 --> 1

149 1 0.500 0 --> 1

node_60 --> node_59 15 1 0.333 0 --> 1

34 1 0.400 0 ==> 2

66 1 0.143 0 ==> 1

97 1 0.250 1 ==> 0

99 1 0.125 0 ==> 1

102 1 0.333 0 ==> 1

node_59 --> node_58 19 1 0.111 1 ==> 0

26 1 0.500 0 ==> 1

39 1 0.200 1 --> 0

46 1 0.100 0 --> 1

82 1 0.200 0 --> 1

114 1 0.286 0 --> 1

121 1 0.250 0 --> 1

123 1 0.143 1 --> 0

135 1 0.125 1 --> 0

node_58 --> node_57 15 1 0.333 1 --> 0

20 1 0.500 0 ==> 1

22 1 0.143 1 ==> 0

84 1 1.000 0 ==> 1

90 1 0.400 1 ==> 0

114 1 0.286 1 --> 2

117 1 0.143 0 ==> 1

node_57 --> Physignathus cocincinus 16 1 0.125 1 ==> 0

62 1 0.143 0 ==> 1

68 1 0.200 1 --> 0

72 1 0.286 0 --> 1

73 1 0.286 1 ==> 0

79 1 0.400 2 --> 0

97 1 0.250 0 ==> 1

98 1 0.125 1 ==> 0

106 1 0.143 0 ==> 1

112 1 0.667 1 ==> 2

node_57 --> Agama agama 4 1 0.091 0 ==> 1

46 1 0.100 1 --> 0

49 1 0.500 0 ==> 1

80 1 0.500 0 ==> 2

119 1 0.143 1 --> 0

124 1 0.200 0 ==> 1

135 1 0.125 0 --> 1

node_58 --> Brookesia superciliaris 5 1 0.500 0 ==> 1

9 1 0.333 0 ==> 1

23 1 0.111 0 ==> 1

25 1 0.333 0 ==> 1

29 1 0.250 0 ==> 1

31 1 0.200 1 ==> 2

38 1 0.250 1 ==> 0

39 1 0.200 0 --> 2

47 1 0.091 0 ==> 1

50 1 0.429 2 ==> 3

53 1 0.111 0 --> 1

81 1 0.500 0 ==> 1

107 1 0.500 1 ==> 0

110 1 1.000 0 ==> 1

113 1 0.250 1 ==> 2

118 1 0.333 0 ==> 1

129 1 0.250 1 ==> 0

138 1 0.250 0 ==> 1

139 1 0.167 0 ==> 1

144 1 0.500 0 ==> 1

node_59 --> Leiolepis belliana 4 1 0.091 0 ==> 1

48 1 0.333 0 ==> 1

49 1 0.500 0 ==> 1

58 1 0.500 0 ==> 1

63 1 0.500 0 ==> 1

79 1 0.400 2 ==> 1

87 1 0.250 0 ==> 1

122 1 0.111 0 --> 1

148 1 0.500 0 ==> 1

node_60 --> Priscagama gobiensis 22 1 0.143 1 ==> 0

47 1 0.091 0 ==> 1

53 1 0.111 0 --> 1

90 1 0.400 1 ==> 0

105 1 0.100 0 ==> 1

node_64 --> Elgaria multicarinata 7 1 0.200 0 ==> 1

16 1 0.125 1 ==> 0

46 1 0.100 0 ==> 1

51 1 0.222 0 ==> 1

71 1 0.500 0 --> 1

78 1 0.250 0 ==> 1

100 1 0.143 0 ==> 1

116 1 0.400 0 --> 2

129 1 0.250 1 --> 0

node_63 --> Plestiodon fasciatus 1 1 0.200 0 ==> 1

6 1 0.333 0 ==> 1

20 1 0.500 0 ==> 1

30 1 0.333 0 --> 1

67 1 0.286 0 ==> 1

73 1 0.286 1 ==> 0

116 1 0.400 0 ==> 1

127 1 0.333 0 ==> 1

node_62 --> Eublepharis macularius 31 1 0.200 0 ==> 1

43 1 0.500 0 ==> 1

44 1 0.500 0 ==> 1

46 1 0.100 0 ==> 1

48 1 0.333 0 ==> 1

51 1 0.222 0 --> 2

66 1 0.143 0 --> 1

82 1 0.200 0 ==> 1

94 1 0.182 0 ==> 2

95 1 0.200 0 ==> 2

100 1 0.143 0 ==> 1

104 1 0.200 0 --> 1

114 1 0.286 0 ==> 1

125 1 0.143 0 ==> 1

126 1 0.167 0 ==> 1

node_61 --> Diphydontosaurus avonis 23 1 0.111 1 ==> 0

94 1 0.182 0 ==> 1

95 1 0.200 0 ==> 1

node_61 --> Sphenodon punctatus 1 1 0.200 0 ==> 2

3 1 0.111 0 ==> 1

25 1 0.333 0 --> 1

35 1 0.500 0 ==> 1

45 1 0.143 0 --> 1

52 1 0.250 0 ==> 2

61 1 0.125 0 --> 1

66 1 0.143 0 --> 1

77 1 0.500 0 --> 1

93 1 0.500 0 ==> 1

102 1 0.333 0 --> 1

103 1 0.143 0 --> 1

116 1 0.400 0 --> 2

124 1 0.200 0 --> 1

139 1 0.167 0 --> 1

141 1 0.333 0 --> 1

Parsimony analysis with scaffold based on Zheng and Wiens [5]

/---------------------------------------------------- YPM 8287

|

| /----------------- Basiliscus basiliscus

| |

| /-------69 /--------- Corytophanes cristatus

| | \------68

/-------74 | \--------- Laemanctus longpipes

| | /-------70

| | | +-------------------------- Geiseltaliellus maarius

| | | |

| | /-------72 \-------------------------- Suzanniwanna patriciana

| | | |

| | | | /--------- Crotaphytus collaris

| \------73 \------------------------71

| | \--------- Gambelia wislizenii

| |

| \-------------------------------------------- Enyaliodes oshaughnessyi

|

| /----------------- Polychrus acutirostris

| |

| /-------41 /--------- Anolis ricordi

/-------67 | \------40

| | | \--------- Anolis cristatellus

| | /-------43

| | | | /--------- Phrynosoma platyrhinos

| | | | |

| | | \---------------42--------- Petrosaurus thalassinus

| | | |

| | | \--------- Sceloporus undulatus

| | /-------48

| | | | /----------------- Pristidactylus torquatus

| | | | |

| | | | /-------45 /--------- Oplurus quadrimaculatus

| | | | | \------44

| | | \-------47 \--------- Chalarodon madagascariensis

/-------66 | | |

| | \---------------52 | /--------- Phymaturus palluma

| | | \---------------46

| | | \--------- Liolaemus pictus

| | |

| | | /--------- Stenocercus scapularis

| | | /------49

| | | | \--------- Microlophus occipitalis

| | \-------------------------51

| | | /--------- Tropidurus torquatus

/------65 | \------50

| | | \--------- Plica umbra

| | |

| | | /----------------- Dipsosaurus dorsalis

| | | |

| | \---------------------------------------------------54 /--------- Brachylophus fasciatus

| | \------53

| | \--------- Iguana iguana

/-------64 |

| | \------------------------------------------------------------------------------- Leiocephalus personatus

| |

| | /--------- Physignathus cocincinus

| | /------55

| | | \--------- Agama agama

| | /-------56

/-------63 | /-------57 \----------------- Brookesia superciliaris

| | | | |

| | \--------------------------------------------------58 \-------------------------- Leiolepis belliana

| | |

/-------62 | \----------------------------------- Priscagama gobiensis

| | |

| | \------------------------------------------------------------------------------------------------ Saichangurvel davidsoni

/------61 |

| | \--------------------------------------------------------------------------------------------------------- Elgaria multicarinata

/-------60 |

| | \------------------------------------------------------------------------------------------------------------------ Plestiodon fasciatus

| |

| \-------------------------------------------------------------------------------------------------------------------------- Eublepharis macularius

59

+----------------------------------------------------------------------------------------------------------------------------------- Diphydontosaurus avonis

|

\----------------------------------------------------------------------------------------------------------------------------------- Sphenodon punctatus

Branch Character Steps CI Change

-------------------------------------------------------------------------

node_59 --> node_60 16 1 0.111 0 --> 1

41 1 0.333 0 --> 1

53 1 0.125 0 ==> 1

54 1 0.500 0 ==> 1

64 1 0.250 1 ==> 0

67 1 0.250 0 --> 1

83 1 0.333 0 --> 1

91 1 0.250 0 --> 1

92 1 0.250 0 --> 1

98 1 0.143 1 --> 0

99 1 0.125 1 ==> 0

100 1 0.125 0 --> 1

106 1 0.125 0 --> 1

119 1 0.143 0 --> 1

122 1 0.125 0 --> 1

135 1 0.125 0 --> 1

node_60 --> node_61 27 1 0.200 0 --> 1

47 1 0.091 0 --> 1

88 1 0.222 0 --> 1

120 1 0.125 0 --> 1

129 1 0.250 0 --> 1

136 1 0.333 1 ==> 0

node_61 --> node_62 16 1 0.111 1 --> 0

39 1 0.182 0 ==> 1

59 1 0.167 1 ==> 0

98 1 0.143 0 --> 1

106 1 0.125 1 --> 0

107 1 0.500 0 --> 1

119 1 0.143 1 --> 0

123 1 0.143 0 ==> 1

node_62 --> node_63 18 1 0.200 0 --> 1

19 1 0.111 0 ==> 1

22 1 0.143 0 ==> 1

23 1 0.125 1 ==> 0

27 1 0.200 1 --> 0

43 1 0.500 0 ==> 1

44 1 0.500 0 ==> 1

47 1 0.091 1 --> 0

50 1 0.429 0 --> 1

53 1 0.125 1 ==> 0

54 1 0.500 1 ==> 0

72 1 0.400 2 --> 1

79 1 0.400 0 --> 2

90 1 0.400 0 ==> 1

91 1 0.250 1 --> 0

92 1 0.250 1 --> 0

97 1 0.250 0 ==> 1

120 1 0.125 1 --> 0

135 1 0.125 1 --> 0

node_63 --> node_64 1 1 0.214 0 --> 1

31 1 0.222 0 --> 1

33 1 1.000 0 ==> 1

35 1 0.500 0 ==> 1

41 1 0.333 1 ==> 0

50 1 0.429 1 --> 2

88 1 0.222 1 --> 2

node_64 --> node_65 10 1 0.250 0 ==> 1

31 1 0.222 1 --> 2

37 1 0.200 0 --> 1

74 1 0.500 0 ==> 1

76 1 0.250 0 ==> 1

80 1 0.500 0 ==> 1

94 1 0.182 0 ==> 2

95 1 0.182 0 ==> 2

98 1 0.143 1 --> 0

125 1 0.167 0 ==> 1

128 1 0.250 0 --> 1

133 1 0.167 0 --> 1

134 1 0.200 0 --> 1

node_65 --> node_66 23 1 0.125 0 --> 1

68 1 0.250 0 ==> 1

111 1 0.333 0 --> 1

113 1 0.222 1 --> 0

122 1 0.125 1 ==> 0

node_66 --> node_67 18 1 0.200 1 ==> 0

73 1 0.400 0 --> 1

83 1 0.333 1 --> 2

88 1 0.222 2 --> 1

100 1 0.125 1 --> 0

145 1 0.200 0 --> 1

node_67 --> node_74 3 1 0.100 0 --> 1

19 1 0.111 1 ==> 0

31 1 0.222 2 --> 1

45 1 0.167 0 --> 1

94 1 0.182 2 ==> 0

95 1 0.182 2 ==> 0

96 1 0.500 0 ==> 1

103 1 0.167 0 ==> 1

117 1 0.167 0 --> 1

119 1 0.143 0 --> 1

133 1 0.167 1 --> 0

134 1 0.200 1 --> 0

node_74 --> YPM 8287 41 1 0.333 0 ==> 1

node_74 --> node_73 17 1 0.500 0 --> 1

105 1 0.111 0 ==> 1

node_73 --> node_72 18 1 0.200 0 --> 1

37 1 0.200 1 ==> 0

61 1 0.125 0 --> 1

94 1 0.182 0 --> 1

95 1 0.182 0 --> 1

115 1 0.125 0 --> 1

151 1 0.500 0 ==> 1

152 1 0.500 0 ==> 1

node_72 --> node_70 11 1 0.250 0 --> 1

27 1 0.200 0 ==> 1

30 1 0.333 0 ==> 1

46 1 0.111 0 ==> 1

51 1 0.250 0 ==> 1

52 1 0.250 0 ==> 2

68 1 0.250 1 --> 0

72 1 0.400 1 --> 2

76 1 0.250 1 --> 0

109 1 0.286 1 ==> 2

111 1 0.333 1 --> 2

125 1 0.167 1 ==> 0

139 1 0.143 0 --> 1

149 1 0.500 0 --> 1

node_70 --> node_69 1 1 0.214 1 ==> 0

4 1 0.083 0 ==> 1

36 1 0.250 0 ==> 1

40 1 0.250 0 ==> 1

50 1 0.429 2 ==> 3

55 1 1.000 0 ==> 1

62 1 0.143 0 ==> 1

88 1 0.222 1 --> 2

94 1 0.182 1 ==> 2

node_69 --> Basiliscus basiliscus 2 1 0.250 0 ==> 1

65 1 0.500 0 ==> 1

74 1 0.500 1 ==> 0

112 1 0.667 1 ==> 2

115 1 0.125 1 --> 0

133 1 0.167 0 ==> 1

134 1 0.200 0 ==> 1

142 1 1.000 0 ==> 1

node_69 --> node_68 11 1 0.250 1 --> 0

53 1 0.125 0 ==> 1

56 1 1.000 0 --> 1

59 1 0.167 0 ==> 1

60 1 0.333 0 ==> 1

81 1 0.500 0 ==> 1

114 1 0.286 0 ==> 1

node_68 --> Corytophanes cristatus 21 1 0.500 0 ==> 1

27 1 0.200 1 ==> 0

29 1 0.333 0 ==> 1

34 1 0.400 0 ==> 2

39 1 0.182 1 ==> 2

42 1 0.250 0 ==> 1

57 1 1.000 0 ==> 1

85 1 0.500 0 ==> 1

86 1 0.167 0 ==> 1

95 1 0.182 1 ==> 2

97 1 0.250 1 ==> 0

105 1 0.111 1 ==> 0

108 1 0.250 0 ==> 1

121 1 0.333 0 ==> 1

node_68 --> Laemanctus longpipes 3 1 0.100 1 ==> 0

22 1 0.143 1 ==> 0

47 1 0.091 0 ==> 1

88 1 0.222 2 --> 1

122 1 0.125 0 ==> 1

node_70 --> Geiseltaliellus maarius 34 1 0.400 0 ==> 1

100 1 0.125 0 ==> 1

101 1 0.500 0 ==> 1

103 1 0.167 1 ==> 0

node_70 --> Suzanniwanna patriciana 18 1 0.200 1 --> 0

node_72 --> node_71 3 1 0.100 1 --> 0

16 1 0.111 0 ==> 1

19 1 0.111 0 ==> 1

22 1 0.143 1 ==> 0

23 1 0.125 1 ==> 0

34 1 0.400 0 ==> 2

38 1 0.250 0 ==> 1

45 1 0.167 1 --> 0

64 1 0.250 0 ==> 1

67 1 0.250 1 --> 2

83 1 0.333 2 --> 0

126 1 0.167 0 ==> 1

135 1 0.125 0 ==> 1

node_71 --> Crotaphytus collaris 47 1 0.091 0 ==> 1

61 1 0.125 1 --> 0

89 1 0.333 0 ==> 1

92 1 0.250 0 ==> 1

94 1 0.182 1 --> 0

95 1 0.182 1 --> 0

117 1 0.167 1 ==> 0

119 1 0.143 1 --> 0

node_71 --> Gambelia wislizenii 17 1 0.500 1 --> 0

105 1 0.111 1 ==> 0

106 1 0.125 0 ==> 1

115 1 0.125 1 --> 0

node_73 --> Enyaliodes oshaughnessyi 1 1 0.214 1 --> 0

4 1 0.083 0 ==> 1

6 1 0.333 0 ==> 1

9 1 0.333 0 ==> 1

26 1 0.500 0 ==> 1

40 1 0.250 0 ==> 1

42 1 0.250 0 ==> 1

47 1 0.091 0 ==> 1

62 1 0.143 0 ==> 1

63 1 0.500 0 ==> 1

86 1 0.167 0 ==> 1

88 1 0.222 1 ==> 2

91 1 0.250 0 ==> 1

100 1 0.125 0 ==> 1

102 1 0.333 0 ==> 1

113 1 0.222 0 --> 2

136 1 0.333 0 ==> 1

node_67 --> node_52 1 1 0.214 1 --> 2

12 1 0.250 0 --> 1

16 1 0.111 0 --> 1

23 1 0.125 1 --> 0

39 1 0.182 1 ==> 0

99 1 0.125 0 --> 1

106 1 0.125 0 ==> 1

111 1 0.333 1 --> 0

113 1 0.222 0 --> 1

138 1 0.167 0 --> 1

139 1 0.143 0 --> 1

node_52 --> node_48 98 1 0.143 0 ==> 1

125 1 0.167 1 ==> 0

135 1 0.125 0 --> 1

node_48 --> node_43 40 1 0.250 0 ==> 1

53 1 0.125 0 ==> 1

66 1 0.143 0 --> 1

72 1 0.400 1 ==> 0

73 1 0.400 1 ==> 2

80 1 0.500 1 --> 2

86 1 0.167 0 --> 1

99 1 0.125 1 --> 0

128 1 0.250 1 ==> 0

139 1 0.143 1 --> 0

node_43 --> node_41 1 1 0.214 2 ==> 0

3 1 0.100 0 --> 1

4 1 0.083 0 --> 1

11 1 0.250 0 ==> 1

13 1 1.000 1 --> 0

16 1 0.111 1 --> 0

19 1 0.111 1 ==> 0

23 1 0.125 0 ==> 1

29 1 0.333 0 ==> 1

31 1 0.222 2 --> 1

37 1 0.200 1 ==> 0

45 1 0.167 0 ==> 1

46 1 0.111 0 ==> 1

47 1 0.091 0 --> 1

51 1 0.250 0 ==> 1

59 1 0.167 0 --> 1

60 1 0.333 0 --> 1

61 1 0.125 0 --> 1

62 1 0.143 0 --> 1

76 1 0.250 1 ==> 0

78 1 0.333 0 ==> 1

82 1 0.250 0 ==> 1

87 1 0.333 0 ==> 1

108 1 0.250 0 ==> 1

109 1 0.286 1 ==> 2

114 1 0.286 0 --> 1

116 1 0.400 0 --> 2

117 1 0.167 0 ==> 1

118 1 0.400 0 ==> 1

121 1 0.333 0 ==> 1

132 1 1.000 0 ==> 1

133 1 0.167 1 ==> 0

134 1 0.200 1 ==> 0

147 1 0.500 0 ==> 2

node_41 --> Polychrus acutirostris 6 1 0.333 0 ==> 1

7 1 0.200 0 ==> 1

9 1 0.333 0 ==> 1

12 1 0.250 1 --> 0

28 1 1.000 0 ==> 1

32 1 0.667 0 ==> 1

39 1 0.182 0 ==> 1

92 1 0.250 0 ==> 1

105 1 0.111 0 ==> 1

106 1 0.125 1 ==> 0

111 1 0.333 0 ==> 2

113 1 0.222 1 ==> 2

114 1 0.286 1 --> 2

115 1 0.125 0 ==> 1

122 1 0.125 0 ==> 1

124 1 0.200 0 ==> 1

129 1 0.250 1 ==> 0

node_41 --> node_40 22 1 0.143 1 ==> 0

36 1 0.250 0 ==> 1

50 1 0.429 2 ==> 1

52 1 0.250 0 ==> 2

65 1 0.500 0 ==> 2

66 1 0.143 1 --> 0

75 1 1.000 0 --> 1

77 1 0.500 0 --> 1

85 1 0.500 0 ==> 1

86 1 0.167 1 --> 0

89 1 0.333 0 ==> 1

99 1 0.125 0 --> 1

100 1 0.125 0 ==> 1

139 1 0.143 0 --> 1

141 1 0.333 0 ==> 1

143 1 0.500 0 ==> 1

152 1 0.500 0 --> 1

node_40 --> Anolis ricordi 30 1 0.333 0 ==> 1

59 1 0.167 1 --> 0

101 1 0.500 0 ==> 1

111 1 0.333 0 ==> 1

128 1 0.250 0 ==> 1

node_40 --> Anolis cristatellus 3 1 0.100 1 --> 0

4 1 0.083 1 --> 0

10 1 0.250 1 ==> 0

31 1 0.222 1 --> 2

47 1 0.091 1 --> 0

53 1 0.125 1 ==> 0

60 1 0.333 1 --> 0

61 1 0.125 1 --> 0

62 1 0.143 1 --> 0

node_43 --> node_42 67 1 0.250 1 --> 0

71 1 0.500 0 ==> 1

94 1 0.182 2 ==> 1

95 1 0.182 2 ==> 1

123 1 0.143 1 --> 0

127 1 0.333 0 ==> 1

130 1 0.333 0 --> 1

138 1 0.167 1 --> 0

145 1 0.200 1 --> 0

146 1 1.000 0 ==> 1

148 1 0.500 0 ==> 1

node_42 --> Phrynosoma platyrhinos 4 1 0.083 0 ==> 1

12 1 0.250 1 --> 0

15 1 0.333 0 ==> 1

19 1 0.111 1 ==> 0

21 1 0.500 0 ==> 1

25 1 0.333 0 ==> 1

32 1 0.667 0 ==> 2

39 1 0.182 0 ==> 2

42 1 0.250 0 ==> 1

46 1 0.111 0 ==> 1

47 1 0.091 0 ==> 1

51 1 0.250 0 ==> 2

59 1 0.167 0 ==> 1

62 1 0.143 0 ==> 1

88 1 0.222 1 ==> 0

90 1 0.400 1 ==> 0

94 1 0.182 1 ==> 0

95 1 0.182 1 ==> 0

99 1 0.125 0 --> 1

104 1 0.200 0 ==> 1

114 1 0.286 0 ==> 1

115 1 0.125 0 ==> 1

124 1 0.200 0 ==> 1

130 1 0.333 1 --> 0

136 1 0.333 0 ==> 1

node_42 --> Petrosaurus thalassinus 1 1 0.214 2 ==> 1

7 1 0.200 0 ==> 1

67 1 0.250 0 --> 1

83 1 0.333 2 ==> 1

109 1 0.286 1 ==> 0

113 1 0.222 1 ==> 0

node_42 --> Sceloporus undulatus 53 1 0.125 1 ==> 0

65 1 0.500 0 ==> 2

76 1 0.250 1 ==> 0

105 1 0.111 0 ==> 1

120 1 0.125 0 ==> 1

123 1 0.143 0 --> 1

125 1 0.167 0 ==> 1

138 1 0.167 0 --> 1

145 1 0.200 0 --> 1

node_48 --> node_47 12 1 0.250 1 --> 0

113 1 0.222 1 --> 0

node_47 --> node_45 3 1 0.100 0 --> 1

19 1 0.111 1 --> 0

39 1 0.182 0 --> 1

64 1 0.250 0 --> 1

78 1 0.333 0 --> 1

95 1 0.182 2 ==> 1

96 1 0.500 0 ==> 1

105 1 0.111 0 ==> 1

118 1 0.400 0 --> 1

135 1 0.125 1 --> 0

138 1 0.167 1 --> 0

143 1 0.500 0 ==> 1

node_45 --> Pristidactylus torquatus 1 1 0.214 2 ==> 1

16 1 0.111 1 --> 0

22 1 0.143 1 ==> 0

23 1 0.125 0 ==> 1

37 1 0.200 1 ==> 0

46 1 0.111 0 ==> 1

47 1 0.091 0 ==> 1

51 1 0.250 0 ==> 1

61 1 0.125 0 ==> 1

82 1 0.250 0 ==> 1

103 1 0.167 0 ==> 1

106 1 0.125 1 ==> 0

120 1 0.125 0 ==> 1

126 1 0.167 0 ==> 1

133 1 0.167 1 ==> 0

141 1 0.333 0 ==> 1

147 1 0.500 0 ==> 2

node_45 --> node_44 2 1 0.250 0 --> 1

4 1 0.083 0 --> 1

18 1 0.200 0 --> 1

34 1 0.400 0 --> 2

83 1 0.333 2 ==> 0

104 1 0.200 0 --> 1

113 1 0.222 0 --> 1

117 1 0.167 0 --> 1

118 1 0.400 1 --> 2

125 1 0.167 0 ==> 1

131 1 1.000 0 ==> 1

144 1 0.500 0 ==> 1

node_44 --> Oplurus quadrimaculatus 36 1 0.250 0 ==> 1

39 1 0.182 1 --> 0

62 1 0.143 0 ==> 1

94 1 0.182 2 ==> 1

109 1 0.286 1 ==> 2

127 1 0.333 0 ==> 1

135 1 0.125 0 --> 1

137 1 1.000 0 ==> 1

node_44 --> Chalarodon madagascariensis 3 1 0.100 1 --> 0

19 1 0.111 0 --> 1

27 1 0.200 0 ==> 1

64 1 0.250 1 --> 0

99 1 0.125 1 --> 0

122 1 0.125 0 ==> 1

128 1 0.250 1 ==> 0

node_47 --> node_46 51 1 0.250 0 ==> 2

67 1 0.250 1 --> 2

68 1 0.250 1 --> 0

100 1 0.125 0 ==> 1

109 1 0.286 1 --> 0

140 1 1.000 0 ==> 1

node_46 --> Phymaturus palluma 1 1 0.214 2 ==> 3

2 1 0.250 0 ==> 1

4 1 0.083 0 ==> 1

16 1 0.111 1 --> 0

59 1 0.167 0 ==> 1

86 1 0.167 0 ==> 1

88 1 0.222 1 ==> 2

94 1 0.182 2 ==> 0

95 1 0.182 2 ==> 0

98 1 0.143 1 ==> 0

99 1 0.125 1 --> 0

112 1 0.667 1 ==> 0

123 1 0.143 1 ==> 0

124 1 0.200 0 ==> 1

node_46 --> Liolaemus pictus 36 1 0.250 0 ==> 1

87 1 0.333 0 ==> 1

120 1 0.125 0 ==> 1

122 1 0.125 0 ==> 1

126 1 0.167 0 ==> 1

node_52 --> node_51 50 1 0.429 2 ==> 1

79 1 0.400 2 --> 1

80 1 0.500 1 --> 0

104 1 0.200 0 ==> 1

126 1 0.167 0 ==> 1

127 1 0.333 0 --> 1

130 1 0.333 0 --> 1

145 1 0.200 1 --> 0

147 1 0.500 0 ==> 2

node_51 --> node_49 46 1 0.111 0 ==> 1

120 1 0.125 0 ==> 1

node_49 --> Stenocercus scapularis 1 1 0.214 2 ==> 1

10 1 0.250 1 ==> 0

19 1 0.111 1 ==> 0

37 1 0.200 1 ==> 0

39 1 0.182 0 ==> 1

45 1 0.167 0 ==> 1

48 1 0.333 0 ==> 1

115 1 0.125 0 ==> 1

117 1 0.167 0 ==> 1

122 1 0.125 0 ==> 1

147 1 0.500 2 ==> 1

node_49 --> Microlophus occipitalis 86 1 0.167 0 ==> 1

node_51 --> node_50 7 1 0.200 0 ==> 1

113 1 0.222 1 --> 2

123 1 0.143 1 ==> 0

node_50 --> Tropidurus torquatus 1 1 0.214 2 ==> 3

88 1 0.222 1 ==> 2

node_50 --> Plica umbra 3 1 0.100 0 ==> 1

4 1 0.083 0 ==> 1

10 1 0.250 1 ==> 0

42 1 0.250 0 ==> 1

66 1 0.143 0 ==> 1

98 1 0.143 0 ==> 1

105 1 0.111 0 ==> 1

119 1 0.143 0 ==> 1

node_66 --> node_54 2 1 0.250 0 ==> 1

4 1 0.083 0 --> 1

24 1 1.000 0 ==> 1

40 1 0.250 0 ==> 1

58 1 0.500 0 ==> 1

65 1 0.500 0 ==> 1

90 1 0.400 1 ==> 2

91 1 0.250 0 ==> 1

109 1 0.286 1 ==> 2

116 1 0.400 0 ==> 1

118 1 0.400 0 --> 1

150 1 1.000 0 ==> 1

151 1 0.500 0 ==> 1

node_54 --> Dipsosaurus dorsalis 38 1 0.250 0 ==> 1

50 1 0.429 2 ==> 3

120 1 0.125 0 --> 1

123 1 0.143 1 ==> 0

node_54 --> node_53 3 1 0.100 0 ==> 1

31 1 0.222 2 --> 0

61 1 0.125 0 ==> 1

79 1 0.400 2 ==> 1

105 1 0.111 0 ==> 1

108 1 0.250 0 ==> 1

111 1 0.333 1 --> 2

115 1 0.125 0 ==> 1

133 1 0.167 1 --> 0

134 1 0.200 1 --> 0

node_53 --> Brachylophus fasciatus 4 1 0.083 1 --> 0

67 1 0.250 1 --> 2

node_53 --> Iguana iguana 11 1 0.250 0 ==> 1

31 1 0.222 0 --> 1

51 1 0.250 0 ==> 1

52 1 0.250 0 ==> 2

72 1 0.400 1 ==> 0

103 1 0.167 0 ==> 1

126 1 0.167 0 ==> 1

node_65 --> Leiocephalus personatus 1 1 0.214 1 --> 2

5 1 0.500 0 ==> 1

7 1 0.200 0 ==> 1

39 1 0.182 1 ==> 0

66 1 0.143 0 ==> 1

67 1 0.250 1 --> 2

104 1 0.200 0 ==> 1

106 1 0.125 0 ==> 1

108 1 0.250 0 ==> 1

120 1 0.125 0 --> 1

127 1 0.333 0 ==> 1

138 1 0.167 0 ==> 1

139 1 0.143 0 --> 1

node_64 --> node_58 1 1 0.214 1 --> 3

8 1 1.000 0 ==> 1

14 1 1.000 0 ==> 1

16 1 0.111 0 --> 1

32 1 0.667 0 --> 1

38 1 0.250 0 ==> 1

45 1 0.167 0 ==> 1

61 1 0.125 0 ==> 1

67 1 0.250 1 --> 0

69 1 1.000 0 --> 1

70 1 1.000 0 --> 1

83 1 0.333 1 --> 2

89 1 0.333 0 ==> 1

93 1 0.500 0 ==> 1

100 1 0.125 1 --> 0

103 1 0.167 0 --> 1

109 1 0.286 1 --> 2

115 1 0.125 0 --> 1

119 1 0.143 0 --> 1

127 1 0.333 0 --> 2

145 1 0.200 0 --> 1

149 1 0.500 0 --> 1

node_58 --> node_57 15 1 0.333 0 --> 1

34 1 0.400 0 ==> 2

66 1 0.143 0 ==> 1

97 1 0.250 1 ==> 0

99 1 0.125 0 ==> 1

102 1 0.333 0 ==> 1

node_57 --> node_56 19 1 0.111 1 ==> 0

26 1 0.500 0 ==> 1

39 1 0.182 1 --> 0

46 1 0.111 0 --> 1

82 1 0.250 0 --> 1

114 1 0.286 0 --> 1

121 1 0.333 0 --> 1

122 1 0.125 1 --> 0

123 1 0.143 1 --> 0

node_56 --> node_55 15 1 0.333 1 --> 0

20 1 0.500 0 ==> 1

22 1 0.143 1 ==> 0

84 1 1.000 0 ==> 1

90 1 0.400 1 ==> 0

114 1 0.286 1 --> 2

117 1 0.167 0 ==> 1

node_55 --> Physignathus cocincinus 16 1 0.111 1 ==> 0

62 1 0.143 0 ==> 1

79 1 0.400 2 --> 0

97 1 0.250 0 ==> 1

98 1 0.143 1 --> 0

106 1 0.125 0 ==> 1

112 1 0.667 1 ==> 2

node_55 --> Agama agama 4 1 0.083 0 ==> 1

46 1 0.111 1 --> 0

49 1 0.500 0 ==> 1

68 1 0.250 0 ==> 1

72 1 0.400 1 ==> 0

73 1 0.400 0 --> 1

80 1 0.500 0 ==> 2

119 1 0.143 1 --> 0

124 1 0.200 0 ==> 1

135 1 0.125 0 --> 1

node_56 --> Brookesia superciliaris 5 1 0.500 0 ==> 1

9 1 0.333 0 ==> 1

23 1 0.125 0 ==> 1

25 1 0.333 0 ==> 1

29 1 0.333 0 ==> 1

31 1 0.222 1 --> 2

38 1 0.250 1 ==> 0

39 1 0.182 0 --> 2

47 1 0.091 0 ==> 1

50 1 0.429 2 ==> 3

53 1 0.125 0 ==> 1

81 1 0.500 0 ==> 1

107 1 0.500 1 ==> 0

110 1 1.000 0 ==> 1

113 1 0.222 1 ==> 2

118 1 0.400 0 ==> 1

129 1 0.250 1 ==> 0

138 1 0.167 0 ==> 1

139 1 0.143 0 --> 1

144 1 0.500 0 ==> 1

node_57 --> Leiolepis belliana 4 1 0.083 0 ==> 1

48 1 0.333 0 ==> 1

49 1 0.500 0 ==> 1

58 1 0.500 0 ==> 1

63 1 0.500 0 ==> 1

79 1 0.400 2 ==> 1

87 1 0.333 0 ==> 1

135 1 0.125 0 --> 1

148 1 0.500 0 ==> 1

node_58 --> Priscagama gobiensis 22 1 0.143 1 ==> 0

47 1 0.091 0 ==> 1

53 1 0.125 0 ==> 1

90 1 0.400 1 ==> 0

105 1 0.111 0 ==> 1

node_62 --> Elgaria multicarinata 7 1 0.200 0 ==> 1

46 1 0.111 0 ==> 1

51 1 0.250 0 ==> 1

71 1 0.500 0 --> 1

73 1 0.400 0 --> 1

78 1 0.333 0 ==> 1

116 1 0.400 0 --> 2

129 1 0.250 1 --> 0

node_61 --> Plestiodon fasciatus 1 1 0.214 0 ==> 1

6 1 0.333 0 ==> 1

20 1 0.500 0 ==> 1

30 1 0.333 0 --> 1

100 1 0.125 1 --> 0

116 1 0.400 0 ==> 1

127 1 0.333 0 ==> 1

node_60 --> Eublepharis macularius 31 1 0.222 0 ==> 1

43 1 0.500 0 ==> 1

44 1 0.500 0 ==> 1

46 1 0.111 0 ==> 1

48 1 0.333 0 ==> 1

51 1 0.250 0 --> 2

66 1 0.143 0 --> 1

82 1 0.250 0 ==> 1

94 1 0.182 0 ==> 2

95 1 0.182 0 ==> 2

104 1 0.200 0 --> 1

114 1 0.286 0 ==> 1

125 1 0.167 0 ==> 1

126 1 0.167 0 ==> 1

node_59 --> Diphydontosaurus avonis 23 1 0.125 1 ==> 0

94 1 0.182 0 ==> 1

95 1 0.182 0 ==> 1

node_59 --> Sphenodon punctatus 1 1 0.214 0 ==> 2

3 1 0.100 0 ==> 1

25 1 0.333 0 --> 1

35 1 0.500 0 ==> 1

45 1 0.167 0 --> 1

52 1 0.250 0 ==> 2

61 1 0.125 0 --> 1

66 1 0.143 0 --> 1

73 1 0.400 0 --> 1

77 1 0.500 0 --> 1

93 1 0.500 0 ==> 1

102 1 0.333 0 --> 1

103 1 0.167 0 --> 1

116 1 0.400 0 --> 2

124 1 0.200 0 --> 1

139 1 0.143 0 --> 1

141 1 0.333 0 --> 1

Bayesian analysis with scaffold based on Burbrink et al [3]

/-------- YPM 8287

/---------------76

| \-------- Enyaliodes oshaughnessyi

/------75

| | /---------------- Pristidactylus torquatus

| | |

| \-------74 /-------- Oplurus quadrimaculatus

/------72 \------73

| | \-------- Chalarodon madagascariensis

| |

/------71 \--------------------------------- Polychrus acutirostris

| |

| | /-------- Phymaturus palluma

| \-------------------------------70

| \-------- Liolaemus pictus

|

| /---------------- Basiliscus basiliscus

| |

/------69 /-------63 /-------- Corytophanes cristatus

| | | \------62

| | /------65 \-------- Laemanctus longpipes

| | | |

| | | | /-------- Geiseltaliellus maarius

| | /------66 \---------------64

| | | | \-------- Suzanniwanna patriciana

| | | |

/------61 \------68 \--------------------------------- Leiocephalus personatus

| | |

| | | /-------- Crotaphytus collaris

| | \-------------------------------67

| | \-------- Gambelia wislizenii

| |

| | /-------- Stenocercus scapularis

| | /------58

/-------57 | | \-------- Microlophus occipitalis

| | \---------------------------------------60

| | | /-------- Tropidurus torquatus

| | \------59

| | \-------- Plica umbra

| |

/------56 | /-------- Anolis ricordi

| | \-------------------------------------------------------40

| | \-------- Anolis cristatellus

| |

| | /---------------- Dipsosaurus dorsalis

| | |

/------55 \--------------------------------------------------------42 /-------- Brachylophus fasciatus

| | \------41

| | \-------- Iguana iguana

| |

| | /-------- Phrynosoma platyrhinos

| | /------43

| | | \-------- Petrosaurus thalassinus

| \----------------------------------------------------------------44

/------54 \---------------- Sceloporus undulatus

| |

| | /-------- Physignathus cocincinus

| | /------45

| | | \-------- Agama agama

| | /-------47

/------53 | | | /-------- Leiolepis belliana

| | | | \------46

| | \---------------------------------------------------------------48 \-------- Brookesia superciliaris

| | |

/-------52 | \------------------------- Priscagama gobiensis

| | |

| | \-------------------------------------------------------------------------------------------------- Saichangurvel davidsoni

/------51 |

| | \---------------------------------------------------------------------------------------------------------- Elgaria multicarinata

/------50 |

| | \------------------------------------------------------------------------------------------------------------------- Plestiodon fasciatus

| |

| \--------------------------------------------------------------------------------------------------------------------------- Eublepharis macularius

49

+----------------------------------------------------------------------------------------------------------------------------------- Diphydontosaurus avonis

|

\----------------------------------------------------------------------------------------------------------------------------------- Sphenodon punctatus

Branch Character Steps CI Change

-------------------------------------------------------------------------

node_49 --> node_50 16 1 0.125 0 ==> 1

41 1 0.333 0 --> 1

53 1 0.125 0 ==> 1

54 1 0.500 0 ==> 1

64 1 0.250 1 ==> 0

83 1 0.333 0 --> 2

91 1 0.250 0 --> 1

92 1 0.250 0 --> 1

98 1 0.125 1 --> 0

99 1 0.125 1 ==> 0

106 1 0.111 0 --> 1

119 1 0.143 0 --> 1

122 1 0.111 0 --> 1

135 1 0.125 0 --> 1

node_50 --> node_51 27 1 0.200 0 --> 1

47 1 0.091 0 --> 1

88 1 0.222 0 --> 1

120 1 0.125 0 --> 1

129 1 0.250 0 --> 1

136 1 0.333 1 ==> 0

node_51 --> node_52 39 1 0.167 0 --> 1

59 1 0.167 1 ==> 0

68 1 0.250 0 --> 1

98 1 0.125 0 --> 1

106 1 0.111 1 --> 0

107 1 0.500 0 --> 1

109 1 0.250 1 --> 2

119 1 0.143 1 --> 0

123 1 0.167 0 ==> 1

node_52 --> node_53 19 1 0.111 0 ==> 1

22 1 0.143 0 --> 1

23 1 0.125 1 ==> 0

27 1 0.200 1 --> 0

43 1 0.500 0 ==> 1

44 1 0.500 0 ==> 1

47 1 0.091 1 --> 0

50 1 0.429 0 --> 1

53 1 0.125 1 ==> 0

54 1 0.500 1 ==> 0

72 1 0.333 2 --> 0

79 1 0.400 0 --> 2

90 1 0.400 0 --> 1

91 1 0.250 1 --> 0

92 1 0.250 1 --> 0

97 1 0.250 0 ==> 1

120 1 0.125 1 --> 0

122 1 0.111 1 --> 0

145 1 0.200 0 --> 1

node_53 --> node_54 1 1 0.188 0 --> 2

31 1 0.182 0 --> 1

33 1 1.000 0 ==> 1

35 1 0.500 0 ==> 1

41 1 0.333 1 ==> 0

50 1 0.429 1 --> 2

node_54 --> node_55 10 1 0.250 0 ==> 1

12 1 0.250 0 --> 1

31 1 0.182 1 --> 2

37 1 0.143 0 --> 1

39 1 0.167 1 --> 0

40 1 0.200 0 --> 1

73 1 0.286 1 --> 2

74 1 0.500 0 ==> 1

76 1 0.200 0 --> 1

80 1 0.500 0 --> 1

94 1 0.182 0 --> 1

95 1 0.167 0 --> 1

106 1 0.111 0 --> 1

125 1 0.143 0 --> 1

133 1 0.143 0 --> 1

134 1 0.143 0 --> 1

node_55 --> node_56 16 1 0.125 1 ==> 0

23 1 0.125 0 --> 1

67 1 0.333 0 ==> 2

94 1 0.182 1 --> 2

95 1 0.167 1 --> 2

98 1 0.125 1 --> 0

100 1 0.125 0 --> 1

118 1 0.400 0 --> 1

128 1 0.250 0 ==> 1

135 1 0.125 1 --> 0

node_56 --> node_57 40 1 0.200 1 --> 0

50 1 0.429 2 --> 1

99 1 0.125 0 --> 1

117 1 0.143 0 --> 1

138 1 0.167 0 ==> 1

139 1 0.167 0 ==> 1

147 1 0.400 0 --> 2

152 1 0.500 0 --> 1

node_57 --> node_61 23 1 0.125 1 --> 0

72 1 0.333 0 --> 1

73 1 0.286 2 --> 1

100 1 0.125 1 --> 0

109 1 0.250 2 --> 1

118 1 0.400 1 --> 0

node_61 --> node_69 12 1 0.250 1 --> 0

31 1 0.182 2 --> 1

39 1 0.167 0 --> 1

50 1 0.429 1 --> 2

96 1 0.333 0 --> 1

99 1 0.125 1 --> 0

106 1 0.111 1 --> 0

113 1 0.222 1 ==> 0

147 1 0.400 2 --> 0

node_69 --> node_71 4 1 0.091 0 --> 1

125 1 0.143 1 --> 0

152 1 0.500 1 --> 0

node_71 --> node_72 1 1 0.188 2 --> 0

3 1 0.125 0 ==> 1

6 1 0.333 0 --> 1

19 1 0.111 1 ==> 0

23 1 0.125 0 ==> 1

45 1 0.143 0 --> 1

47 1 0.091 0 --> 1

67 1 0.333 2 ==> 1

78 1 0.333 0 ==> 1

105 1 0.111 0 ==> 1

113 1 0.222 0 --> 2

118 1 0.400 0 ==> 1

133 1 0.143 1 --> 0

134 1 0.143 1 --> 0

139 1 0.167 1 --> 0

node_72 --> node_75 95 1 0.167 2 --> 0

103 1 0.143 0 --> 1

138 1 0.167 1 ==> 0

node_75 --> node_76 42 1 0.250 0 --> 1

63 1 0.500 0 --> 1

94 1 0.182 2 ==> 0

136 1 0.333 0 --> 1

node_76 --> YPM 8287 41 1 0.333 0 ==> 1

47 1 0.091 1 --> 0

105 1 0.111 1 ==> 0

node_76 --> Enyaliodes oshaughnessyi 9 1 0.333 0 ==> 1

26 1 0.333 0 ==> 1

40 1 0.200 0 ==> 1

62 1 0.143 0 ==> 1

86 1 0.167 0 ==> 1

88 1 0.222 1 ==> 2

91 1 0.250 0 ==> 1

100 1 0.125 0 ==> 1

102 1 0.333 0 ==> 1

node_75 --> node_74 1 1 0.188 0 --> 1

6 1 0.333 1 --> 0

31 1 0.182 1 --> 2

45 1 0.143 1 --> 0

64 1 0.250 0 --> 1

95 1 0.167 0 --> 1

98 1 0.125 0 ==> 1

99 1 0.125 0 --> 1

113 1 0.222 2 --> 0

134 1 0.143 0 --> 1

139 1 0.167 0 --> 1

143 1 0.500 0 ==> 1

node_74 --> Pristidactylus torquatus 4 1 0.091 1 ==> 0

22 1 0.143 1 ==> 0

37 1 0.143 1 ==> 0

46 1 0.100 0 ==> 1

51 1 0.222 0 ==> 1

61 1 0.125 0 ==> 1

82 1 0.200 0 ==> 1

117 1 0.143 1 ==> 0

120 1 0.125 0 ==> 1

126 1 0.167 0 ==> 1

141 1 0.333 0 ==> 1

147 1 0.400 0 ==> 2

node_74 --> node_73 1 1 0.188 1 --> 2

2 1 0.250 0 --> 1

16 1 0.125 0 ==> 1

18 1 0.200 0 --> 1

23 1 0.125 1 ==> 0

34 1 0.400 0 --> 2

47 1 0.091 1 --> 0

83 1 0.333 2 ==> 0

103 1 0.143 1 --> 0

104 1 0.200 0 --> 1

106 1 0.111 0 ==> 1

113 1 0.222 0 --> 1

118 1 0.400 1 ==> 2

125 1 0.143 0 ==> 1

131 1 1.000 0 ==> 1

133 1 0.143 0 ==> 1

144 1 0.500 0 ==> 1

node_73 --> Oplurus quadrimaculatus 36 1 0.250 0 ==> 1

39 1 0.167 1 ==> 0

62 1 0.143 0 ==> 1

94 1 0.182 2 ==> 1

109 1 0.250 1 ==> 2

127 1 0.333 0 ==> 1

135 1 0.125 0 ==> 1

137 1 1.000 0 ==> 1

node_73 --> Chalarodon madagascariensis 3 1 0.125 1 ==> 0

19 1 0.111 0 ==> 1

27 1 0.200 0 ==> 1

64 1 0.250 1 --> 0

99 1 0.125 1 --> 0

122 1 0.111 0 ==> 1

128 1 0.250 1 ==> 0

node_72 --> Polychrus acutirostris 7 1 0.200 0 ==> 1

9 1 0.333 0 ==> 1

11 1 0.200 0 ==> 1

28 1 1.000 0 ==> 1

29 1 0.250 0 ==> 1

32 1 0.667 0 ==> 1

37 1 0.143 1 ==> 0

40 1 0.200 0 ==> 1

46 1 0.100 0 ==> 1

51 1 0.222 0 ==> 1

53 1 0.125 0 ==> 1

59 1 0.167 0 ==> 1

60 1 0.333 0 ==> 1

61 1 0.125 0 ==> 1

62 1 0.143 0 ==> 1

66 1 0.143 0 ==> 1

72 1 0.333 1 ==> 0

73 1 0.286 1 ==> 2

76 1 0.200 1 ==> 0

82 1 0.200 0 ==> 1

86 1 0.167 0 ==> 1

87 1 0.250 0 ==> 1

92 1 0.250 0 ==> 1

108 1 0.200 0 ==> 1

109 1 0.250 1 ==> 2

111 1 0.333 0 ==> 2

114 1 0.286 0 ==> 2

115 1 0.125 0 ==> 1

121 1 0.250 0 ==> 1

122 1 0.111 0 ==> 1

124 1 0.200 0 ==> 1

128 1 0.250 1 ==> 0

129 1 0.250 1 ==> 0

132 1 0.500 0 ==> 1

147 1 0.400 0 ==> 2

node_71 --> node_70 31 1 0.182 1 --> 2

39 1 0.167 1 --> 0

51 1 0.222 0 ==> 2

68 1 0.250 1 --> 0

96 1 0.333 1 --> 0

100 1 0.125 0 ==> 1

109 1 0.250 1 --> 0

117 1 0.143 1 --> 0

135 1 0.125 0 --> 1

140 1 1.000 0 ==> 1

node_70 --> Phymaturus palluma 1 1 0.188 2 --> 3

2 1 0.250 0 ==> 1

59 1 0.167 0 ==> 1

86 1 0.167 0 ==> 1

88 1 0.222 1 ==> 2

94 1 0.182 2 ==> 0

95 1 0.167 2 ==> 0

112 1 0.667 1 ==> 0

123 1 0.167 1 ==> 0

124 1 0.200 0 ==> 1

node_70 --> Liolaemus pictus 4 1 0.091 1 --> 0

16 1 0.125 0 ==> 1

36 1 0.250 0 ==> 1

87 1 0.250 0 ==> 1

98 1 0.125 0 ==> 1

99 1 0.125 0 ==> 1

120 1 0.125 0 ==> 1

122 1 0.111 0 ==> 1

126 1 0.167 0 ==> 1

node_69 --> node_68 1 1 0.188 2 --> 1

18 1 0.200 0 ==> 1

37 1 0.143 1 --> 0

95 1 0.167 2 --> 1

138 1 0.167 1 --> 0

151 1 0.500 0 ==> 1

node_68 --> node_66 68 1 0.250 1 ==> 0

88 1 0.222 1 --> 2

node_66 --> node_65 3 1 0.125 0 ==> 1

11 1 0.200 0 --> 1

17 1 0.500 0 --> 1

19 1 0.111 1 ==> 0

23 1 0.125 0 ==> 1

27 1 0.200 0 ==> 1

30 1 0.333 0 ==> 1

45 1 0.143 0 ==> 1

46 1 0.100 0 ==> 1

51 1 0.222 0 ==> 1

52 1 0.250 0 ==> 2

61 1 0.125 0 --> 1

67 1 0.333 2 --> 1

72 1 0.333 1 --> 2

76 1 0.200 1 --> 0

105 1 0.111 0 ==> 1

109 1 0.250 1 ==> 2

111 1 0.333 0 --> 2

115 1 0.125 0 --> 1

119 1 0.143 0 ==> 1

125 1 0.143 1 --> 0

149 1 0.500 0 --> 1

node_65 --> node_63 1 1 0.188 1 --> 0

4 1 0.091 0 ==> 1

36 1 0.250 0 ==> 1

40 1 0.200 0 ==> 1

50 1 0.429 2 ==> 3

55 1 1.000 0 ==> 1

62 1 0.143 0 ==> 1

103 1 0.143 0 --> 1

node_63 --> Basiliscus basiliscus 2 1 0.250 0 ==> 1

65 1 0.500 0 ==> 1

74 1 0.500 1 ==> 0

112 1 0.667 1 ==> 2

115 1 0.125 1 --> 0

142 1 1.000 0 ==> 1

node_63 --> node_62 11 1 0.200 1 --> 0

53 1 0.125 0 ==> 1

56 1 1.000 0 --> 1

59 1 0.167 0 ==> 1

60 1 0.333 0 ==> 1

81 1 0.500 0 ==> 1

114 1 0.286 0 ==> 1

133 1 0.143 1 --> 0

134 1 0.143 1 --> 0

node_62 --> Corytophanes cristatus 21 1 0.500 0 ==> 1

27 1 0.200 1 ==> 0

29 1 0.250 0 ==> 1

34 1 0.400 0 ==> 2

39 1 0.167 1 ==> 2

42 1 0.250 0 ==> 1

57 1 1.000 0 ==> 1

85 1 0.500 0 ==> 1

86 1 0.167 0 ==> 1

95 1 0.167 1 ==> 2

97 1 0.250 1 ==> 0

105 1 0.111 1 ==> 0

108 1 0.200 0 ==> 1

121 1 0.250 0 ==> 1

node_62 --> Laemanctus longpipes 3 1 0.125 1 ==> 0

22 1 0.143 1 ==> 0

47 1 0.091 0 ==> 1

88 1 0.222 2 --> 1

122 1 0.111 0 ==> 1

node_65 --> node_64 18 1 0.200 1 --> 0

88 1 0.222 2 --> 1

94 1 0.182 2 ==> 1

node_64 --> Geiseltaliellus maarius 34 1 0.400 0 ==> 1

100 1 0.125 0 ==> 1

101 1 0.500 0 ==> 1

node_66 --> Leiocephalus personatus 1 1 0.188 1 --> 2

5 1 0.500 0 ==> 1

7 1 0.200 0 ==> 1

31 1 0.182 1 --> 2

37 1 0.143 0 --> 1

39 1 0.167 1 --> 0

66 1 0.143 0 ==> 1

73 1 0.286 1 ==> 0

83 1 0.333 2 ==> 1

95 1 0.167 1 --> 2

96 1 0.333 1 --> 0

100 1 0.125 0 ==> 1

104 1 0.200 0 ==> 1

106 1 0.111 0 --> 1

108 1 0.200 0 ==> 1

113 1 0.222 0 ==> 1

120 1 0.125 0 ==> 1

122 1 0.111 0 ==> 1

127 1 0.333 0 ==> 1

138 1 0.167 0 --> 1

145 1 0.200 1 ==> 0

node_68 --> node_67 16 1 0.125 0 ==> 1

22 1 0.143 1 ==> 0

34 1 0.400 0 ==> 2

38 1 0.250 0 ==> 1

64 1 0.250 0 ==> 1

83 1 0.333 2 --> 0

94 1 0.182 2 --> 0

103 1 0.143 0 ==> 1

111 1 0.333 0 ==> 1

126 1 0.167 0 ==> 1

133 1 0.143 1 --> 0

134 1 0.143 1 --> 0

135 1 0.125 0 --> 1

139 1 0.167 1 ==> 0

node_67 --> Crotaphytus collaris 17 1 0.500 0 ==> 1

47 1 0.091 0 ==> 1

89 1 0.333 0 ==> 1

92 1 0.250 0 ==> 1

95 1 0.167 1 --> 0

105 1 0.111 0 ==> 1

115 1 0.125 0 ==> 1

117 1 0.143 1 --> 0

node_67 --> Gambelia wislizenii 61 1 0.125 0 ==> 1

94 1 0.182 0 --> 1

106 1 0.111 0 --> 1

119 1 0.143 0 ==> 1

node_61 --> node_60 16 1 0.125 0 ==> 1

79 1 0.400 2 --> 1

80 1 0.500 1 --> 0

104 1 0.200 0 ==> 1

117 1 0.143 1 --> 0

126 1 0.167 0 ==> 1

127 1 0.333 0 --> 1

130 1 0.333 0 --> 1

145 1 0.200 1 --> 0

node_60 --> node_58 46 1 0.100 0 ==> 1

120 1 0.125 0 ==> 1

node_58 --> Stenocercus scapularis 1 1 0.188 2 ==> 1

10 1 0.250 1 ==> 0

19 1 0.111 1 ==> 0

37 1 0.143 1 ==> 0

39 1 0.167 0 --> 1

45 1 0.143 0 ==> 1

48 1 0.333 0 ==> 1

115 1 0.125 0 ==> 1

117 1 0.143 0 --> 1

122 1 0.111 0 ==> 1

147 1 0.400 2 ==> 1

node_58 --> Microlophus occipitalis 86 1 0.167 0 ==> 1

node_60 --> node_59 7 1 0.200 0 ==> 1

113 1 0.222 1 --> 2

123 1 0.167 1 ==> 0

node_59 --> Tropidurus torquatus 1 1 0.188 2 ==> 3

88 1 0.222 1 ==> 2

node_59 --> Plica umbra 3 1 0.125 0 ==> 1

4 1 0.091 0 ==> 1

10 1 0.250 1 ==> 0

42 1 0.250 0 ==> 1

66 1 0.143 0 ==> 1

98 1 0.125 0 ==> 1

105 1 0.111 0 ==> 1

119 1 0.143 0 ==> 1

node_57 --> node_40 1 1 0.188 2 --> 0

11 1 0.200 0 ==> 1

13 1 1.000 1 ==> 0

19 1 0.111 1 ==> 0

22 1 0.143 1 ==> 0

29 1 0.250 0 ==> 1

36 1 0.250 0 ==> 1

37 1 0.143 1 ==> 0

45 1 0.143 0 ==> 1

46 1 0.100 0 ==> 1

51 1 0.222 0 ==> 1

52 1 0.250 0 ==> 2

65 1 0.500 0 ==> 2

75 1 1.000 0 --> 1

76 1 0.200 1 --> 0

77 1 0.500 0 --> 1

78 1 0.333 0 --> 1

82 1 0.200 0 ==> 1

85 1 0.500 0 ==> 1

87 1 0.250 0 ==> 1

89 1 0.333 0 ==> 1

98 1 0.125 0 --> 1

108 1 0.200 0 ==> 1

114 1 0.286 0 ==> 1

116 1 0.400 0 ==> 2

121 1 0.250 0 ==> 1

125 1 0.143 1 --> 0

132 1 0.500 0 ==> 1

133 1 0.143 1 --> 0

134 1 0.143 1 --> 0

135 1 0.125 0 --> 1

141 1 0.333 0 ==> 1

143 1 0.500 0 ==> 1

node_40 --> Anolis ricordi 3 1 0.125 0 ==> 1

4 1 0.091 0 ==> 1

30 1 0.333 0 ==> 1

31 1 0.182 2 --> 1

47 1 0.091 0 ==> 1

53 1 0.125 0 ==> 1

60 1 0.333 0 ==> 1

61 1 0.125 0 ==> 1

62 1 0.143 0 ==> 1

101 1 0.500 0 ==> 1

111 1 0.333 0 ==> 1

node_40 --> Anolis cristatellus 10 1 0.250 1 ==> 0

59 1 0.167 0 ==> 1

128 1 0.250 1 ==> 0

node_56 --> node_42 1 1 0.188 2 ==> 1

2 1 0.250 0 ==> 1

4 1 0.091 0 --> 1

12 1 0.250 1 --> 0

18 1 0.200 0 ==> 1

24 1 1.000 0 ==> 1

39 1 0.167 0 --> 1

58 1 0.500 0 ==> 1

65 1 0.500 0 ==> 1

73 1 0.286 2 --> 0

83 1 0.333 2 --> 1

88 1 0.222 1 ==> 2

90 1 0.400 1 ==> 2

91 1 0.250 0 ==> 1

106 1 0.111 1 --> 0

111 1 0.333 0 --> 1

113 1 0.222 1 ==> 0

116 1 0.400 0 ==> 1

145 1 0.200 1 --> 0

150 1 1.000 0 ==> 1

151 1 0.500 0 ==> 1

node_42 --> Dipsosaurus dorsalis 38 1 0.250 0 ==> 1

50 1 0.429 2 ==> 3

120 1 0.125 0 ==> 1

123 1 0.167 1 ==> 0

node_42 --> node_41 3 1 0.125 0 ==> 1

31 1 0.182 2 --> 0

61 1 0.125 0 ==> 1

79 1 0.400 2 ==> 1

105 1 0.111 0 ==> 1

108 1 0.200 0 ==> 1

111 1 0.333 1 --> 2

115 1 0.125 0 ==> 1

133 1 0.143 1 --> 0

134 1 0.143 1 --> 0

node_41 --> Brachylophus fasciatus 4 1 0.091 1 --> 0

72 1 0.333 0 ==> 1

node_41 --> Iguana iguana 11 1 0.200 0 ==> 1

31 1 0.182 0 --> 1

51 1 0.222 0 ==> 1

52 1 0.250 0 ==> 2

67 1 0.333 2 ==> 1

103 1 0.143 0 ==> 1

126 1 0.167 0 ==> 1

node_55 --> node_44 66 1 0.143 0 ==> 1

71 1 0.500 0 --> 1

80 1 0.500 1 --> 2

86 1 0.167 0 --> 1

109 1 0.250 2 --> 1

127 1 0.333 0 ==> 1

130 1 0.333 0 --> 1

146 1 1.000 0 ==> 1

148 1 0.500 0 ==> 1

node_44 --> node_43 7 1 0.200 0 --> 1

53 1 0.125 0 ==> 1

123 1 0.167 1 ==> 0

125 1 0.143 1 --> 0

145 1 0.200 1 --> 0

node_43 --> Phrynosoma platyrhinos 4 1 0.091 0 ==> 1

12 1 0.250 1 --> 0

15 1 0.500 0 ==> 1

19 1 0.111 1 ==> 0

21 1 0.500 0 ==> 1

25 1 0.333 0 ==> 1

32 1 0.667 0 ==> 2

39 1 0.167 0 ==> 2

42 1 0.250 0 ==> 1

46 1 0.100 0 ==> 1

47 1 0.091 0 ==> 1

51 1 0.222 0 ==> 2

59 1 0.167 0 ==> 1

62 1 0.143 0 ==> 1

88 1 0.222 1 ==> 0

90 1 0.400 1 ==> 0

94 1 0.182 1 --> 0

95 1 0.167 1 --> 0

99 1 0.125 0 ==> 1

104 1 0.200 0 ==> 1

114 1 0.286 0 ==> 1

115 1 0.125 0 ==> 1

124 1 0.200 0 ==> 1

130 1 0.333 1 --> 0

136 1 0.333 0 ==> 1

node_43 --> Petrosaurus thalassinus 1 1 0.188 2 ==> 1

67 1 0.333 0 ==> 1

83 1 0.333 2 ==> 1

109 1 0.250 1 ==> 0

113 1 0.222 1 ==> 0

node_44 --> Sceloporus undulatus 65 1 0.500 0 ==> 2

76 1 0.200 1 --> 0

105 1 0.111 0 ==> 1

120 1 0.125 0 ==> 1

138 1 0.167 0 ==> 1

node_54 --> node_48 1 1 0.188 2 --> 3

8 1 1.000 0 ==> 1

14 1 1.000 0 ==> 1

18 1 0.200 0 --> 1

22 1 0.143 1 --> 0

32 1 0.667 0 --> 1

38 1 0.250 0 ==> 1

45 1 0.143 0 ==> 1

61 1 0.125 0 ==> 1

69 1 1.000 0 --> 1

70 1 1.000 0 --> 1

89 1 0.333 0 ==> 1

90 1 0.400 1 --> 0

93 1 0.500 0 ==> 1

103 1 0.143 0 --> 1

115 1 0.125 0 --> 1

119 1 0.143 0 --> 1

127 1 0.333 0 --> 2

149 1 0.500 0 --> 1

node_48 --> node_47 19 1 0.111 1 --> 0

26 1 0.333 0 --> 1

34 1 0.400 0 ==> 2

66 1 0.143 0 ==> 1

97 1 0.250 1 --> 0

99 1 0.125 0 ==> 1

102 1 0.333 0 ==> 1

node_47 --> node_45 20 1 0.500 0 ==> 1

39 1 0.167 1 --> 0

82 1 0.200 0 ==> 1

84 1 1.000 0 ==> 1

114 1 0.286 0 ==> 2

117 1 0.143 0 ==> 1

121 1 0.250 0 ==> 1

123 1 0.167 1 ==> 0

node_45 --> Physignathus cocincinus 16 1 0.125 1 ==> 0

46 1 0.100 0 ==> 1

62 1 0.143 0 ==> 1

68 1 0.250 1 --> 0

72 1 0.333 0 ==> 1

73 1 0.286 1 ==> 0

79 1 0.400 2 --> 0

97 1 0.250 0 --> 1

98 1 0.125 1 ==> 0

106 1 0.111 0 --> 1

112 1 0.667 1 ==> 2

135 1 0.125 1 ==> 0

node_45 --> Agama agama 4 1 0.091 0 ==> 1

49 1 0.500 0 ==> 1

80 1 0.500 0 ==> 2

119 1 0.143 1 --> 0

124 1 0.200 0 ==> 1

node_47 --> node_46 9 1 0.333 0 --> 1

15 1 0.500 0 ==> 1

22 1 0.143 0 --> 1

31 1 0.182 1 --> 2

50 1 0.429 2 --> 3

58 1 0.500 0 --> 1

63 1 0.500 0 --> 1

79 1 0.400 2 --> 1

90 1 0.400 0 --> 1

122 1 0.111 0 --> 1

node_46 --> Leiolepis belliana 4 1 0.091 0 ==> 1

19 1 0.111 0 --> 1

26 1 0.333 1 --> 0

48 1 0.333 0 ==> 1

49 1 0.500 0 ==> 1

87 1 0.250 0 ==> 1

148 1 0.500 0 ==> 1

node_46 --> Brookesia superciliaris 5 1 0.500 0 ==> 1

23 1 0.125 0 ==> 1

25 1 0.333 0 ==> 1

29 1 0.250 0 ==> 1

38 1 0.250 1 ==> 0

39 1 0.167 1 --> 2

46 1 0.100 0 ==> 1

47 1 0.091 0 ==> 1

53 1 0.125 0 ==> 1

81 1 0.500 0 ==> 1

107 1 0.500 1 ==> 0

110 1 1.000 0 ==> 1

113 1 0.222 1 ==> 2

114 1 0.286 0 ==> 1

118 1 0.400 0 ==> 1

129 1 0.250 1 ==> 0

135 1 0.125 1 ==> 0

138 1 0.167 0 ==> 1

139 1 0.167 0 ==> 1

144 1 0.500 0 ==> 1

node_48 --> Priscagama gobiensis 47 1 0.091 0 ==> 1

53 1 0.125 0 ==> 1

105 1 0.111 0 ==> 1

node_52 --> Elgaria multicarinata 7 1 0.200 0 ==> 1

16 1 0.125 1 ==> 0

46 1 0.100 0 ==> 1

51 1 0.222 0 ==> 1

71 1 0.500 0 --> 1

78 1 0.333 0 ==> 1

100 1 0.125 0 ==> 1

116 1 0.400 0 --> 2

129 1 0.250 1 --> 0

node_51 --> Plestiodon fasciatus 1 1 0.188 0 ==> 1

6 1 0.333 0 ==> 1

20 1 0.500 0 ==> 1

30 1 0.333 0 --> 1

67 1 0.333 0 ==> 1

73 1 0.286 1 ==> 0

116 1 0.400 0 ==> 1

127 1 0.333 0 ==> 1

node_50 --> Eublepharis macularius 31 1 0.182 0 ==> 1

43 1 0.500 0 ==> 1

44 1 0.500 0 ==> 1

46 1 0.100 0 ==> 1

48 1 0.333 0 ==> 1

51 1 0.222 0 --> 2

66 1 0.143 0 --> 1

82 1 0.200 0 ==> 1

94 1 0.182 0 ==> 2

95 1 0.167 0 ==> 2

100 1 0.125 0 ==> 1

104 1 0.200 0 --> 1

114 1 0.286 0 ==> 1

125 1 0.143 0 ==> 1

126 1 0.167 0 ==> 1

node_49 --> Diphydontosaurus avonis 23 1 0.125 1 ==> 0

94 1 0.182 0 ==> 1

95 1 0.167 0 ==> 1

node_49 --> Sphenodon punctatus 1 1 0.188 0 ==> 2

3 1 0.125 0 ==> 1

25 1 0.333 0 --> 1

35 1 0.500 0 ==> 1

45 1 0.143 0 --> 1

52 1 0.250 0 ==> 2

61 1 0.125 0 --> 1

66 1 0.143 0 --> 1

77 1 0.500 0 --> 1

93 1 0.500 0 ==> 1

102 1 0.333 0 --> 1

103 1 0.143 0 --> 1

116 1 0.400 0 --> 2

124 1 0.200 0 --> 1

139 1 0.167 0 --> 1

141 1 0.333 0 --> 1

Bayesian analysis with scaffold based on Streicher et al [4]

/-------------------------------------------- YPM 8287

|

| /----------------- Basiliscus basiliscus

| |

/------76 /-------73 /--------- Corytophanes cristatus

| | | \------72

| | /-------74 \--------- Laemanctus longpipes

| | | |

/-------71 \-------75 \-------------------------- Geiseltaliellus maarius

| | |

| | \----------------------------------- Suzanniwanna patriciana

| |

| | /--------- Crotaphytus collaris

| \-----------------------------------------70

| \--------- Gambelia wislizenii

|

| /----------------- Polychrus acutirostris

| |

| /----------------41 /--------- Phymaturus palluma

/-------69 | \------40

| | | \--------- Liolaemus pictus

| | |

| | /-------45 /----------------- Pristidactylus torquatus

| | | | |

| | | | /-------43 /--------- Oplurus quadrimaculatus

| | | | | \------42

| | | \-------44 \--------- Chalarodon madagascariensis

| | | |

| | | \-------------------------- Enyaliodes oshaughnessyi

| \---------------52

| | /--------- Anolis ricordi

| | /------46

/-------68 | | \--------- Anolis cristatellus

| | | /-------47

| | | | \----------------- Leiocephalus personatus

| | | |

| | \----------------51 /--------- Stenocercus scapularis

| | | /------48

| | | | \--------- Microlophus occipitalis

| | \-------50

| | | /--------- Tropidurus torquatus

| | \------49

/------67 | \--------- Plica umbra

| | |

| | | /----------------- Dipsosaurus dorsalis

| | | |

| | \---------------------------------------------------54 /--------- Brachylophus fasciatus

| | \------53

| | \--------- Iguana iguana

| |

| | /--------- Phrynosoma platyrhinos

| | /------55

/-------66 | | \--------- Petrosaurus thalassinus

| | \------------------------------------------------------------56

| | \----------------- Sceloporus undulatus

| |

| | /--------- Physignathus cocincinus

| | /------57

| | | \--------- Agama agama

/-------65 | /-------59

| | | | | /--------- Leiolepis belliana

| | | | \------58

| | \-----------------------------------------------------------60 \--------- Brookesia superciliaris

/-------64 | |

| | | \-------------------------- Priscagama gobiensis

| | |

| | \------------------------------------------------------------------------------------------------ Saichangurvel davidsoni

/------63 |

| | \--------------------------------------------------------------------------------------------------------- Elgaria multicarinata

/-------62 |

| | \------------------------------------------------------------------------------------------------------------------ Plestiodon fasciatus

| |

| \-------------------------------------------------------------------------------------------------------------------------- Eublepharis macularius

61

+----------------------------------------------------------------------------------------------------------------------------------- Diphydontosaurus avonis

|

\----------------------------------------------------------------------------------------------------------------------------------- Sphenodon punctatus

Branch Character Steps CI Change

-------------------------------------------------------------------------

node_61 --> node_62 16 1 0.125 0 ==> 1

41 1 0.333 0 --> 1

53 1 0.125 0 ==> 1

54 1 0.500 0 ==> 1

64 1 0.250 1 ==> 0

83 1 0.333 0 --> 2

91 1 0.250 0 --> 1

92 1 0.250 0 --> 1

98 1 0.125 1 --> 0

99 1 0.125 1 ==> 0

106 1 0.143 0 --> 1

119 1 0.143 0 --> 1

122 1 0.111 0 --> 1

135 1 0.125 0 --> 1

node_62 --> node_63 27 1 0.200 0 --> 1

47 1 0.091 0 --> 1

88 1 0.222 0 --> 1

120 1 0.125 0 --> 1

129 1 0.250 0 --> 1

136 1 0.333 1 ==> 0

node_63 --> node_64 39 1 0.200 0 ==> 1

59 1 0.167 1 ==> 0

68 1 0.200 0 --> 1

98 1 0.125 0 --> 1

106 1 0.143 1 --> 0

107 1 0.500 0 --> 1

119 1 0.143 1 --> 0

123 1 0.167 0 ==> 1

node_64 --> node_65 19 1 0.100 0 ==> 1

22 1 0.143 0 --> 1

23 1 0.111 1 ==> 0

27 1 0.200 1 --> 0

43 1 0.500 0 ==> 1

44 1 0.500 0 ==> 1

47 1 0.091 1 --> 0

50 1 0.429 0 --> 1

53 1 0.125 1 ==> 0

54 1 0.500 1 ==> 0

72 1 0.286 2 --> 0

79 1 0.400 0 --> 2

90 1 0.400 0 --> 1

91 1 0.250 1 --> 0

92 1 0.250 1 --> 0

97 1 0.250 0 ==> 1

120 1 0.125 1 --> 0

122 1 0.111 1 --> 0

145 1 0.200 0 --> 1

node_65 --> node_66 1 1 0.200 0 --> 1

31 1 0.200 0 --> 1

33 1 1.000 0 ==> 1

35 1 0.500 0 ==> 1

41 1 0.333 1 ==> 0

50 1 0.429 1 --> 2

node_66 --> node_67 10 1 0.250 0 ==> 1

31 1 0.200 1 --> 2

37 1 0.167 0 --> 1

40 1 0.200 0 --> 1

74 1 0.500 0 ==> 1

76 1 0.200 0 --> 1

80 1 0.500 0 --> 1

94 1 0.167 0 --> 1

95 1 0.167 0 --> 1

125 1 0.143 0 --> 1

134 1 0.143 0 --> 1

node_67 --> node_68 16 1 0.125 1 ==> 0

23 1 0.111 0 --> 1

67 1 0.286 0 ==> 1

72 1 0.286 0 --> 1

94 1 0.167 1 --> 2

95 1 0.167 1 --> 2

98 1 0.125 1 ==> 0

111 1 0.333 0 --> 1

113 1 0.250 1 ==> 0

128 1 0.250 0 ==> 1

135 1 0.125 1 ==> 0

151 1 0.500 0 --> 1

node_68 --> node_69 40 1 0.200 1 --> 0

117 1 0.143 0 ==> 1

139 1 0.167 0 --> 1

152 1 0.500 0 --> 1

node_69 --> node_71 31 1 0.200 2 --> 1

37 1 0.167 1 ==> 0

94 1 0.167 2 --> 0

95 1 0.167 2 --> 0

96 1 0.500 0 ==> 1

103 1 0.143 0 ==> 1

115 1 0.125 0 --> 1

119 1 0.143 0 --> 1

134 1 0.143 1 --> 0

node_71 --> node_76 3 1 0.111 0 --> 1

11 1 0.200 0 --> 1

19 1 0.100 1 ==> 0

45 1 0.143 0 --> 1

46 1 0.100 0 --> 1

51 1 0.222 0 --> 1

52 1 0.250 0 --> 2

68 1 0.200 1 --> 0

72 1 0.286 1 --> 2

76 1 0.200 1 --> 0

109 1 0.250 1 --> 2

111 1 0.333 1 --> 2

125 1 0.143 1 --> 0

149 1 0.500 0 --> 1

node_76 --> YPM 8287 41 1 0.333 0 ==> 1

node_76 --> node_75 17 1 0.500 0 --> 1

27 1 0.200 0 ==> 1

30 1 0.333 0 ==> 1

61 1 0.125 0 --> 1

94 1 0.167 0 --> 1

95 1 0.167 0 --> 1

105 1 0.111 0 ==> 1

node_75 --> node_74 18 1 0.167 0 --> 1

36 1 0.250 0 --> 1

node_74 --> node_73 1 1 0.200 1 ==> 0

4 1 0.091 0 ==> 1

40 1 0.200 0 ==> 1

50 1 0.429 2 ==> 3

55 1 1.000 0 ==> 1

62 1 0.143 0 ==> 1

88 1 0.222 1 --> 2

94 1 0.167 1 ==> 2

node_73 --> Basiliscus basiliscus 2 1 0.250 0 ==> 1

65 1 0.500 0 ==> 1

74 1 0.500 1 ==> 0

112 1 0.667 1 ==> 2

115 1 0.125 1 --> 0

133 1 0.143 0 ==> 1

134 1 0.143 0 --> 1

142 1 1.000 0 ==> 1

node_73 --> node_72 11 1 0.200 1 --> 0

53 1 0.125 0 ==> 1

56 1 1.000 0 --> 1

59 1 0.167 0 ==> 1

60 1 0.333 0 ==> 1

81 1 0.500 0 ==> 1

114 1 0.286 0 ==> 1

node_72 --> Corytophanes cristatus 21 1 0.500 0 ==> 1

27 1 0.200 1 ==> 0

29 1 0.250 0 ==> 1

34 1 0.400 0 ==> 2

39 1 0.200 1 ==> 2

42 1 0.250 0 ==> 1

57 1 1.000 0 ==> 1

85 1 0.500 0 ==> 1

86 1 0.167 0 ==> 1

95 1 0.167 1 ==> 2

97 1 0.250 1 ==> 0

105 1 0.111 1 ==> 0

108 1 0.250 0 ==> 1

121 1 0.250 0 ==> 1

node_72 --> Laemanctus longpipes 3 1 0.111 1 ==> 0

22 1 0.143 1 ==> 0

47 1 0.091 0 ==> 1

88 1 0.222 2 --> 1

122 1 0.111 0 ==> 1

node_74 --> Geiseltaliellus maarius 34 1 0.400 0 ==> 1

100 1 0.143 0 ==> 1

101 1 0.500 0 ==> 1

103 1 0.143 1 ==> 0

node_71 --> node_70 16 1 0.125 0 ==> 1

18 1 0.167 0 ==> 1

22 1 0.143 1 ==> 0

23 1 0.111 1 --> 0

34 1 0.400 0 ==> 2

38 1 0.250 0 ==> 1

64 1 0.250 0 ==> 1

67 1 0.286 1 --> 2

83 1 0.333 2 --> 0

126 1 0.167 0 ==> 1

135 1 0.125 0 ==> 1

139 1 0.167 1 --> 0

node_70 --> Crotaphytus collaris 17 1 0.500 0 ==> 1

47 1 0.091 0 ==> 1

89 1 0.333 0 ==> 1

92 1 0.250 0 ==> 1

105 1 0.111 0 ==> 1

117 1 0.143 1 ==> 0

119 1 0.143 1 --> 0

node_70 --> Gambelia wislizenii 61 1 0.125 0 ==> 1

94 1 0.167 0 --> 1

95 1 0.167 0 --> 1

106 1 0.143 0 ==> 1

115 1 0.125 1 --> 0

node_69 --> node_52 1 1 0.200 1 --> 2

78 1 0.250 0 --> 1

111 1 0.333 1 --> 0

138 1 0.250 0 --> 1

151 1 0.500 1 --> 0

node_52 --> node_45 3 1 0.111 0 --> 1

4 1 0.091 0 ==> 1

19 1 0.100 1 --> 0

40 1 0.200 0 --> 1

47 1 0.091 0 --> 1

86 1 0.167 0 --> 1

105 1 0.111 0 --> 1

118 1 0.333 0 --> 1

125 1 0.143 1 --> 0

152 1 0.500 1 --> 0

node_45 --> node_41 11 1 0.200 0 --> 1

28 1 1.000 0 --> 1

32 1 0.667 0 --> 1

51 1 0.222 0 --> 1

59 1 0.167 0 --> 1

87 1 0.250 0 --> 1

109 1 0.250 1 --> 0

122 1 0.111 0 --> 1

124 1 0.200 0 --> 1

128 1 0.250 1 --> 0

135 1 0.125 0 --> 1

node_41 --> Polychrus acutirostris 1 1 0.200 2 ==> 0

6 1 0.333 0 ==> 1

7 1 0.200 0 ==> 1

9 1 0.333 0 ==> 1

29 1 0.250 0 ==> 1

31 1 0.200 2 --> 1

37 1 0.167 1 ==> 0

45 1 0.143 0 ==> 1

46 1 0.100 0 ==> 1

53 1 0.125 0 ==> 1

60 1 0.333 0 ==> 1

61 1 0.125 0 ==> 1

62 1 0.143 0 ==> 1

66 1 0.143 0 ==> 1

72 1 0.286 1 ==> 0

73 1 0.286 1 ==> 2

76 1 0.200 1 ==> 0

82 1 0.200 0 ==> 1

92 1 0.250 0 ==> 1

108 1 0.250 0 ==> 1

109 1 0.250 0 --> 2

111 1 0.333 0 ==> 2

113 1 0.250 0 ==> 2

114 1 0.286 0 ==> 2

115 1 0.125 0 ==> 1

121 1 0.250 0 ==> 1

129 1 0.250 1 ==> 0

132 1 0.500 0 ==> 1

134 1 0.143 1 --> 0

139 1 0.167 1 --> 0

147 1 0.400 0 ==> 2

node_41 --> node_40 3 1 0.111 1 --> 0

19 1 0.100 0 --> 1

23 1 0.111 1 --> 0

39 1 0.200 1 ==> 0

47 1 0.091 1 --> 0

51 1 0.222 1 --> 2

67 1 0.286 1 --> 2

68 1 0.200 1 --> 0

78 1 0.250 1 --> 0

100 1 0.143 0 ==> 1

105 1 0.111 1 --> 0

117 1 0.143 1 ==> 0

118 1 0.333 1 --> 0

133 1 0.143 0 --> 1

140 1 1.000 0 ==> 1

node_40 --> Phymaturus palluma 1 1 0.200 2 ==> 3

2 1 0.250 0 ==> 1

87 1 0.250 1 --> 0

88 1 0.222 1 ==> 2

94 1 0.167 2 ==> 0

95 1 0.167 2 --> 0

112 1 0.667 1 ==> 0

122 1 0.111 1 --> 0

123 1 0.167 1 ==> 0

node_40 --> Liolaemus pictus 4 1 0.091 1 ==> 0

16 1 0.125 0 ==> 1

36 1 0.250 0 ==> 1

59 1 0.167 1 --> 0

86 1 0.167 1 --> 0

98 1 0.125 0 ==> 1

99 1 0.125 0 ==> 1

120 1 0.125 0 ==> 1

124 1 0.200 1 --> 0

126 1 0.167 0 ==> 1

node_45 --> node_44 95 1 0.167 2 --> 0

96 1 0.500 0 ==> 1

103 1 0.143 0 --> 1

138 1 0.250 1 --> 0

node_44 --> node_43 40 1 0.200 1 --> 0

64 1 0.250 0 --> 1

86 1 0.167 1 --> 0

95 1 0.167 0 --> 1

98 1 0.125 0 ==> 1

99 1 0.125 0 --> 1

143 1 0.500 0 ==> 1

node_43 --> Pristidactylus torquatus 1 1 0.200 2 ==> 1

4 1 0.091 1 ==> 0

22 1 0.143 1 ==> 0

37 1 0.167 1 ==> 0

46 1 0.100 0 ==> 1

51 1 0.222 0 ==> 1

61 1 0.125 0 ==> 1

82 1 0.200 0 ==> 1

117 1 0.143 1 ==> 0

120 1 0.125 0 ==> 1

126 1 0.167 0 ==> 1

141 1 0.333 0 ==> 1

147 1 0.400 0 ==> 2

node_43 --> node_42 2 1 0.250 0 --> 1

16 1 0.125 0 ==> 1

18 1 0.167 0 --> 1

23 1 0.111 1 --> 0

34 1 0.400 0 --> 2

47 1 0.091 1 --> 0

83 1 0.333 2 ==> 0

103 1 0.143 1 --> 0

104 1 0.200 0 --> 1

106 1 0.143 0 ==> 1

113 1 0.250 0 --> 1

118 1 0.333 1 --> 2

125 1 0.143 0 --> 1

131 1 1.000 0 ==> 1

133 1 0.143 0 ==> 1

144 1 0.500 0 ==> 1

node_42 --> Oplurus quadrimaculatus 36 1 0.250 0 ==> 1

39 1 0.200 1 ==> 0

62 1 0.143 0 ==> 1

94 1 0.167 2 ==> 1

109 1 0.250 1 ==> 2

127 1 0.333 0 ==> 1

135 1 0.125 0 ==> 1

137 1 1.000 0 ==> 1

node_42 --> Chalarodon madagascariensis 3 1 0.111 1 ==> 0

19 1 0.100 0 ==> 1

27 1 0.200 0 ==> 1

64 1 0.250 1 --> 0

99 1 0.125 1 --> 0

122 1 0.111 0 ==> 1

128 1 0.250 1 ==> 0

node_44 --> Enyaliodes oshaughnessyi 1 1 0.200 2 ==> 0

6 1 0.333 0 ==> 1

9 1 0.333 0 ==> 1

26 1 0.333 0 ==> 1

31 1 0.200 2 --> 1

42 1 0.250 0 ==> 1

45 1 0.143 0 ==> 1

62 1 0.143 0 ==> 1

63 1 0.500 0 ==> 1

88 1 0.222 1 ==> 2

91 1 0.250 0 ==> 1

94 1 0.167 2 ==> 0

100 1 0.143 0 ==> 1

102 1 0.333 0 ==> 1

113 1 0.250 0 ==> 2

134 1 0.143 1 --> 0

136 1 0.333 0 ==> 1

139 1 0.167 1 --> 0

node_52 --> node_51 12 1 0.250 0 --> 1

23 1 0.111 1 --> 0

39 1 0.200 1 ==> 0

50 1 0.429 2 --> 1

67 1 0.286 1 --> 2

73 1 0.286 1 --> 0

99 1 0.125 0 --> 1

104 1 0.200 0 --> 1

106 1 0.143 0 ==> 1

113 1 0.250 0 ==> 1

127 1 0.333 0 --> 1

133 1 0.143 0 --> 1

145 1 0.200 1 --> 0

147 1 0.400 0 --> 2

node_51 --> node_47 13 1 1.000 1 --> 0

100 1 0.143 0 ==> 1

108 1 0.250 0 ==> 1

node_47 --> node_46 1 1 0.200 2 ==> 0

11 1 0.200 0 ==> 1

19 1 0.100 1 ==> 0

22 1 0.143 1 ==> 0

23 1 0.111 0 --> 1

29 1 0.250 0 ==> 1

36 1 0.250 0 ==> 1

37 1 0.167 1 ==> 0

45 1 0.143 0 ==> 1

46 1 0.100 0 ==> 1

51 1 0.222 0 ==> 1

52 1 0.250 0 ==> 2

65 1 0.500 0 ==> 2

72 1 0.286 1 --> 0

73 1 0.286 0 --> 2

75 1 1.000 0 --> 1

76 1 0.200 1 --> 0

77 1 0.500 0 --> 1

82 1 0.200 0 ==> 1

85 1 0.500 0 ==> 1

87 1 0.250 0 ==> 1

89 1 0.333 0 ==> 1

98 1 0.125 0 ==> 1

104 1 0.200 1 --> 0

109 1 0.250 1 ==> 2

114 1 0.286 0 ==> 1

116 1 0.400 0 ==> 2

118 1 0.333 0 ==> 1

121 1 0.250 0 ==> 1

125 1 0.143 1 --> 0

127 1 0.333 1 --> 0

132 1 0.500 0 ==> 1

133 1 0.143 1 --> 0

134 1 0.143 1 --> 0

135 1 0.125 0 --> 1

141 1 0.333 0 ==> 1

143 1 0.500 0 ==> 1

145 1 0.200 0 --> 1

node_46 --> Anolis ricordi 3 1 0.111 0 ==> 1

4 1 0.091 0 ==> 1

30 1 0.333 0 ==> 1

31 1 0.200 2 ==> 1

47 1 0.091 0 ==> 1

53 1 0.125 0 ==> 1

60 1 0.333 0 ==> 1

61 1 0.125 0 ==> 1

62 1 0.143 0 ==> 1

101 1 0.500 0 ==> 1

111 1 0.333 0 ==> 1

node_46 --> Anolis cristatellus 10 1 0.250 1 ==> 0

59 1 0.167 0 ==> 1

128 1 0.250 1 ==> 0

node_47 --> Leiocephalus personatus 5 1 0.500 0 ==> 1

7 1 0.200 0 ==> 1

12 1 0.250 1 --> 0

18 1 0.167 0 ==> 1

50 1 0.429 1 --> {23}

66 1 0.143 0 ==> 1

68 1 0.200 1 ==> 0

78 1 0.250 1 --> 0

83 1 0.333 2 ==> 1

88 1 0.222 1 ==> 2

99 1 0.125 1 --> 0

120 1 0.125 0 ==> 1

122 1 0.111 0 ==> 1

147 1 0.400 2 --> 0

node_51 --> node_50 16 1 0.125 0 ==> 1

79 1 0.400 2 --> 1

80 1 0.500 1 --> 0

117 1 0.143 1 --> 0

126 1 0.167 0 ==> 1

130 1 0.333 0 --> 1

node_50 --> node_48 46 1 0.100 0 ==> 1

120 1 0.125 0 ==> 1

node_48 --> Stenocercus scapularis 1 1 0.200 2 ==> 1

10 1 0.250 1 ==> 0

19 1 0.100 1 ==> 0

37 1 0.167 1 ==> 0

39 1 0.200 0 ==> 1

45 1 0.143 0 ==> 1

48 1 0.333 0 ==> 1

115 1 0.125 0 ==> 1

117 1 0.143 0 --> 1

122 1 0.111 0 ==> 1

147 1 0.400 2 ==> 1

node_48 --> Microlophus occipitalis 86 1 0.167 0 ==> 1

node_50 --> node_49 7 1 0.200 0 ==> 1

113 1 0.250 1 --> 2

123 1 0.167 1 ==> 0

node_49 --> Tropidurus torquatus 1 1 0.200 2 ==> 3

88 1 0.222 1 ==> 2

node_49 --> Plica umbra 3 1 0.111 0 ==> 1

4 1 0.091 0 ==> 1

10 1 0.250 1 ==> 0

42 1 0.250 0 ==> 1

66 1 0.143 0 ==> 1

98 1 0.125 0 ==> 1

105 1 0.111 0 ==> 1

119 1 0.143 0 ==> 1

node_68 --> node_54 2 1 0.250 0 ==> 1

4 1 0.091 0 --> 1

18 1 0.167 0 ==> 1

24 1 1.000 0 ==> 1

58 1 0.500 0 ==> 1

65 1 0.500 0 ==> 1

73 1 0.286 1 --> 0

83 1 0.333 2 --> 1

88 1 0.222 1 ==> 2

90 1 0.400 1 ==> 2

91 1 0.250 0 ==> 1

100 1 0.143 0 ==> 1

109 1 0.250 1 ==> 2

116 1 0.400 0 ==> 1

118 1 0.333 0 --> 1

145 1 0.200 1 --> 0

150 1 1.000 0 ==> 1

node_54 --> Dipsosaurus dorsalis 38 1 0.250 0 ==> 1

50 1 0.429 2 ==> 3

120 1 0.125 0 ==> 1

123 1 0.167 1 ==> 0

133 1 0.143 0 ==> 1

node_54 --> node_53 3 1 0.111 0 ==> 1

31 1 0.200 2 --> 0

61 1 0.125 0 ==> 1

79 1 0.400 2 ==> 1

105 1 0.111 0 ==> 1

108 1 0.250 0 ==> 1

111 1 0.333 1 --> 2

115 1 0.125 0 ==> 1

134 1 0.143 1 --> 0

node_53 --> Brachylophus fasciatus 4 1 0.091 1 --> 0

67 1 0.286 1 --> 2

node_53 --> Iguana iguana 11 1 0.200 0 ==> 1

31 1 0.200 0 --> 1

51 1 0.222 0 ==> 1

52 1 0.250 0 ==> 2

72 1 0.286 1 --> 0

103 1 0.143 0 ==> 1

126 1 0.167 0 ==> 1

node_67 --> node_56 1 1 0.200 1 --> 2

12 1 0.250 0 --> 1

39 1 0.200 1 ==> 0

66 1 0.143 0 ==> 1

71 1 0.500 0 --> 1

73 1 0.286 1 ==> 2

80 1 0.500 1 --> 2

86 1 0.167 0 --> 1

106 1 0.143 0 --> 1

127 1 0.333 0 ==> 1

130 1 0.333 0 --> 1

133 1 0.143 0 ==> 1

146 1 1.000 0 ==> 1

148 1 0.500 0 ==> 1

node_56 --> node_55 7 1 0.200 0 --> 1

53 1 0.125 0 ==> 1

123 1 0.167 1 ==> 0

125 1 0.143 1 --> 0

145 1 0.200 1 --> 0

node_55 --> Phrynosoma platyrhinos 4 1 0.091 0 ==> 1

12 1 0.250 1 --> 0

15 1 0.500 0 ==> 1

19 1 0.100 1 ==> 0

21 1 0.500 0 ==> 1

25 1 0.333 0 ==> 1

32 1 0.667 0 ==> 2

39 1 0.200 0 ==> 2

42 1 0.250 0 ==> 1

46 1 0.100 0 ==> 1

47 1 0.091 0 ==> 1

51 1 0.222 0 ==> 2

59 1 0.167 0 ==> 1

62 1 0.143 0 ==> 1

88 1 0.222 1 ==> 0

90 1 0.400 1 ==> 0

94 1 0.167 1 --> 0

95 1 0.167 1 --> 0

99 1 0.125 0 ==> 1

104 1 0.200 0 ==> 1

114 1 0.286 0 ==> 1

115 1 0.125 0 ==> 1

124 1 0.200 0 ==> 1

130 1 0.333 1 --> 0

136 1 0.333 0 ==> 1

node_55 --> Petrosaurus thalassinus 1 1 0.200 2 --> 1

67 1 0.286 0 ==> 1

83 1 0.333 2 ==> 1

109 1 0.250 1 ==> 0

113 1 0.250 1 ==> 0

node_56 --> Sceloporus undulatus 65 1 0.500 0 ==> 2

76 1 0.200 1 --> 0

105 1 0.111 0 ==> 1

120 1 0.125 0 ==> 1

138 1 0.250 0 ==> 1

node_66 --> node_60 1 1 0.200 1 --> 3

8 1 1.000 0 ==> 1

14 1 1.000 0 ==> 1

18 1 0.167 0 --> 1

22 1 0.143 1 --> 0

32 1 0.667 0 --> 1

38 1 0.250 0 ==> 1

45 1 0.143 0 ==> 1

61 1 0.125 0 ==> 1

69 1 1.000 0 --> 1

70 1 1.000 0 --> 1

89 1 0.333 0 ==> 1

90 1 0.400 1 --> 0

93 1 0.500 0 ==> 1

103 1 0.143 0 --> 1

109 1 0.250 1 --> 2

115 1 0.125 0 --> 1

119 1 0.143 0 --> 1

127 1 0.333 0 --> 2

149 1 0.500 0 --> 1

node_60 --> node_59 19 1 0.100 1 --> 0

26 1 0.333 0 --> 1

34 1 0.400 0 ==> 2

66 1 0.143 0 ==> 1

97 1 0.250 1 --> 0

99 1 0.125 0 ==> 1

102 1 0.333 0 ==> 1

node_59 --> node_57 20 1 0.500 0 ==> 1

39 1 0.200 1 ==> 0

82 1 0.200 0 ==> 1

84 1 1.000 0 ==> 1

114 1 0.286 0 ==> 2

117 1 0.143 0 ==> 1

121 1 0.250 0 ==> 1

123 1 0.167 1 ==> 0

node_57 --> Physignathus cocincinus 16 1 0.125 1 ==> 0

46 1 0.100 0 ==> 1

62 1 0.143 0 ==> 1

68 1 0.200 1 --> 0

72 1 0.286 0 --> 1

73 1 0.286 1 ==> 0

79 1 0.400 2 --> 0

97 1 0.250 0 --> 1

98 1 0.125 1 ==> 0

106 1 0.143 0 ==> 1

112 1 0.667 1 ==> 2

135 1 0.125 1 ==> 0

node_57 --> Agama agama 4 1 0.091 0 ==> 1

49 1 0.500 0 ==> 1

80 1 0.500 0 ==> 2

119 1 0.143 1 --> 0

124 1 0.200 0 ==> 1

node_59 --> node_58 9 1 0.333 0 --> 1

15 1 0.500 0 ==> 1

22 1 0.143 0 --> 1

31 1 0.200 1 --> 2

50 1 0.429 2 --> 3

58 1 0.500 0 --> 1

63 1 0.500 0 --> 1

79 1 0.400 2 --> 1

90 1 0.400 0 --> 1

122 1 0.111 0 --> 1

node_58 --> Leiolepis belliana 4 1 0.091 0 ==> 1

19 1 0.100 0 --> 1

26 1 0.333 1 --> 0

48 1 0.333 0 ==> 1

49 1 0.500 0 ==> 1

87 1 0.250 0 ==> 1

148 1 0.500 0 ==> 1

node_58 --> Brookesia superciliaris 5 1 0.500 0 ==> 1

23 1 0.111 0 ==> 1

25 1 0.333 0 ==> 1

29 1 0.250 0 ==> 1

38 1 0.250 1 ==> 0

39 1 0.200 1 ==> 2

46 1 0.100 0 ==> 1

47 1 0.091 0 ==> 1

53 1 0.125 0 ==> 1

81 1 0.500 0 ==> 1

107 1 0.500 1 ==> 0

110 1 1.000 0 ==> 1

113 1 0.250 1 ==> 2

114 1 0.286 0 ==> 1

118 1 0.333 0 ==> 1

129 1 0.250 1 ==> 0

135 1 0.125 1 ==> 0

138 1 0.250 0 ==> 1

139 1 0.167 0 ==> 1

144 1 0.500 0 ==> 1

node_60 --> Priscagama gobiensis 47 1 0.091 0 ==> 1

53 1 0.125 0 ==> 1

105 1 0.111 0 ==> 1

node_64 --> Elgaria multicarinata 7 1 0.200 0 ==> 1

16 1 0.125 1 ==> 0

46 1 0.100 0 ==> 1

51 1 0.222 0 ==> 1

71 1 0.500 0 --> 1

78 1 0.250 0 ==> 1

100 1 0.143 0 ==> 1

116 1 0.400 0 --> 2

129 1 0.250 1 --> 0

node_63 --> Plestiodon fasciatus 1 1 0.200 0 ==> 1

6 1 0.333 0 ==> 1

20 1 0.500 0 ==> 1

30 1 0.333 0 --> 1

67 1 0.286 0 ==> 1

73 1 0.286 1 ==> 0

116 1 0.400 0 ==> 1

127 1 0.333 0 ==> 1

node_62 --> Eublepharis macularius 31 1 0.200 0 ==> 1

43 1 0.500 0 ==> 1

44 1 0.500 0 ==> 1

46 1 0.100 0 ==> 1

48 1 0.333 0 ==> 1

51 1 0.222 0 --> 2

66 1 0.143 0 --> 1

82 1 0.200 0 ==> 1

94 1 0.167 0 ==> 2

95 1 0.167 0 ==> 2

100 1 0.143 0 ==> 1

104 1 0.200 0 --> 1

114 1 0.286 0 ==> 1

125 1 0.143 0 ==> 1

126 1 0.167 0 ==> 1

node_61 --> Diphydontosaurus avonis 23 1 0.111 1 ==> 0

94 1 0.167 0 ==> 1

95 1 0.167 0 ==> 1

node_61 --> Sphenodon punctatus 1 1 0.200 0 ==> 2

3 1 0.111 0 ==> 1

25 1 0.333 0 --> 1

35 1 0.500 0 ==> 1

45 1 0.143 0 --> 1

52 1 0.250 0 ==> 2

61 1 0.125 0 --> 1

66 1 0.143 0 --> 1

77 1 0.500 0 --> 1

93 1 0.500 0 ==> 1

102 1 0.333 0 --> 1

103 1 0.143 0 --> 1

116 1 0.400 0 --> 2

124 1 0.200 0 --> 1

139 1 0.167 0 --> 1

141 1 0.333 0 --> 1

Bayesian analysis with scaffold based on Zheng and Wiens [5]

/------------------------------------------------- YPM 8287

|

| /---------------- Basiliscus basiliscus

| |

| /-------71 /-------- Corytophanes cristatus

/------76 | \------70

| | /------72 \-------- Laemanctus longpipes

| | | |

| | /------73 \------------------------- Geiseltaliellus maarius

| | | |

| \------75 \--------------------------------- Suzanniwanna patriciana

/------69 |

| | | /-------- Crotaphytus collaris

| | \-------------------------------74

| | \-------- Gambelia wislizenii

| |

| \--------------------------------------------------------- Enyaliodes oshaughnessyi

|

| /---------------- Polychrus acutirostris

| |

| /-------41 /-------- Anolis ricordi

| | \------40

| | \-------- Anolis cristatellus

/-------68 /------44

| | | | /-------- Phrynosoma platyrhinos

| | | | /------42

| | | | | \-------- Petrosaurus thalassinus

| | | \-------43

| | | \---------------- Sceloporus undulatus

| | /------49

| | | | /---------------- Pristidactylus torquatus

| | | | |

| | | | /-------46 /-------- Oplurus quadrimaculatus

| | | | | \------45

| | | \------48 \-------- Chalarodon madagascariensis

| | | |

/------67 \----------------------53 | /-------- Phymaturus palluma

| | | \---------------47

| | | \-------- Liolaemus pictus

| | |

| | | /-------- Stenocercus scapularis

| | | /------50

| | | | \-------- Microlophus occipitalis

| | \-----------------------52

| | | /-------- Tropidurus torquatus

/------66 | \------51

| | | \-------- Plica umbra

| | |

| | | /---------------- Dipsosaurus dorsalis

| | | |

| | \--------------------------------------------------------55 /-------- Brachylophus fasciatus

| | \------54

| | \-------- Iguana iguana

/------65 |

| | \---------------------------------------------------------------------------------- Leiocephalus personatus

| |

| | /-------- Physignathus cocincinus

| | /------56

| | | \-------- Agama agama

| | /-------58

/------64 | | | /-------- Leiolepis belliana

| | | | \------57

| | \---------------------------------------------------------------59 \-------- Brookesia superciliaris

| | |

/-------63 | \------------------------- Priscagama gobiensis

| | |

| | \-------------------------------------------------------------------------------------------------- Saichangurvel davidsoni

/------62 |

| | \---------------------------------------------------------------------------------------------------------- Elgaria multicarinata

/------61 |

| | \------------------------------------------------------------------------------------------------------------------- Plestiodon fasciatus

| |

| \--------------------------------------------------------------------------------------------------------------------------- Eublepharis macularius

60

+----------------------------------------------------------------------------------------------------------------------------------- Diphydontosaurus avonis

|

\----------------------------------------------------------------------------------------------------------------------------------- Sphenodon punctatus

Branch Character Steps CI Change

-------------------------------------------------------------------------

node_60 --> node_61 16 1 0.111 0 --> 1

41 1 0.333 0 --> 1

53 1 0.125 0 ==> 1

54 1 0.500 0 ==> 1

64 1 0.250 1 ==> 0

67 1 0.250 0 --> 1

83 1 0.333 0 --> 1

91 1 0.250 0 --> 1

92 1 0.250 0 --> 1

98 1 0.143 1 --> 0

99 1 0.125 1 ==> 0

100 1 0.125 0 --> 1

106 1 0.125 0 --> 1

119 1 0.143 0 --> 1

122 1 0.125 0 --> 1

135 1 0.125 0 --> 1

node_61 --> node_62 27 1 0.200 0 --> 1

47 1 0.091 0 --> 1

88 1 0.222 0 --> 1

120 1 0.125 0 --> 1

129 1 0.250 0 --> 1

136 1 0.333 1 ==> 0

node_62 --> node_63 16 1 0.111 1 --> 0

39 1 0.182 0 ==> 1

59 1 0.167 1 ==> 0

98 1 0.143 0 --> 1

106 1 0.125 1 --> 0

107 1 0.500 0 --> 1

119 1 0.143 1 --> 0

123 1 0.167 0 ==> 1

node_63 --> node_64 18 1 0.200 0 --> 1

19 1 0.100 0 ==> 1

22 1 0.143 0 --> 1

23 1 0.125 1 ==> 0

27 1 0.200 1 --> 0

43 1 0.500 0 ==> 1

44 1 0.500 0 ==> 1

47 1 0.091 1 --> 0

50 1 0.429 0 --> 1

53 1 0.125 1 ==> 0

54 1 0.500 1 ==> 0

72 1 0.400 2 --> 1

79 1 0.400 0 --> 2

90 1 0.400 0 --> 1

91 1 0.250 1 --> 0

92 1 0.250 1 --> 0

97 1 0.250 0 ==> 1

120 1 0.125 1 --> 0

135 1 0.125 1 --> 0

node_64 --> node_65 1 1 0.214 0 --> 1

31 1 0.222 0 --> 1

33 1 1.000 0 ==> 1

35 1 0.500 0 ==> 1

41 1 0.333 1 ==> 0

50 1 0.429 1 --> 2

88 1 0.222 1 --> 2

node_65 --> node_66 10 1 0.250 0 ==> 1

31 1 0.222 1 --> 2

37 1 0.200 0 --> 1

74 1 0.500 0 ==> 1

76 1 0.250 0 ==> 1

80 1 0.500 0 ==> 1

94 1 0.182 0 ==> 2

95 1 0.182 0 ==> 2

98 1 0.143 1 --> 0

125 1 0.167 0 ==> 1

128 1 0.250 0 --> 1

133 1 0.167 0 --> 1

134 1 0.200 0 --> 1

node_66 --> node_67 23 1 0.125 0 --> 1

68 1 0.250 0 ==> 1

111 1 0.333 0 --> 1

113 1 0.222 1 --> 0

122 1 0.125 1 ==> 0

node_67 --> node_68 18 1 0.200 1 ==> 0

73 1 0.400 0 --> 1

83 1 0.333 1 --> 2

88 1 0.222 2 --> 1

100 1 0.125 1 --> 0

145 1 0.250 0 --> 1

node_68 --> node_69 3 1 0.100 0 --> 1

19 1 0.100 1 ==> 0

31 1 0.222 2 --> 1

45 1 0.167 0 --> 1

94 1 0.182 2 ==> 0

95 1 0.182 2 ==> 0

96 1 0.500 0 ==> 1

103 1 0.167 0 ==> 1

105 1 0.100 0 --> 1

117 1 0.167 0 ==> 1

119 1 0.143 0 --> 1

133 1 0.167 1 --> 0

134 1 0.200 1 --> 0

node_69 --> node_76 37 1 0.200 1 --> 0

115 1 0.125 0 --> 1

151 1 0.500 0 --> 1

152 1 0.500 0 --> 1

node_76 --> YPM 8287 41 1 0.333 0 ==> 1

105 1 0.100 1 --> 0

node_76 --> node_75 17 1 0.500 0 --> 1

18 1 0.200 0 --> 1

61 1 0.125 0 --> 1

94 1 0.182 0 --> 1

95 1 0.182 0 --> 1

node_75 --> node_73 11 1 0.250 0 --> 1

27 1 0.200 0 ==> 1

30 1 0.333 0 ==> 1

46 1 0.111 0 --> 1

51 1 0.250 0 ==> 1

52 1 0.250 0 ==> 2

68 1 0.250 1 --> 0

72 1 0.400 1 --> 2

76 1 0.250 1 --> 0

109 1 0.286 1 --> 2

111 1 0.333 1 --> 2

125 1 0.167 1 --> 0

139 1 0.143 0 --> 1

149 1 0.500 0 --> 1

node_73 --> node_72 36 1 0.250 0 --> 1

node_72 --> node_71 1 1 0.214 1 ==> 0

4 1 0.083 0 ==> 1

40 1 0.250 0 ==> 1

50 1 0.429 2 ==> 3

55 1 1.000 0 ==> 1

62 1 0.143 0 ==> 1

88 1 0.222 1 --> 2

94 1 0.182 1 ==> 2

node_71 --> Basiliscus basiliscus 2 1 0.250 0 ==> 1

65 1 0.500 0 ==> 1

74 1 0.500 1 ==> 0

112 1 0.667 1 ==> 2

115 1 0.125 1 --> 0

133 1 0.167 0 ==> 1

134 1 0.200 0 ==> 1

142 1 1.000 0 ==> 1

node_71 --> node_70 11 1 0.250 1 --> 0

53 1 0.125 0 ==> 1

56 1 1.000 0 --> 1

59 1 0.167 0 ==> 1

60 1 0.333 0 ==> 1

81 1 0.500 0 ==> 1

114 1 0.286 0 ==> 1

node_70 --> Corytophanes cristatus 21 1 0.500 0 ==> 1

27 1 0.200 1 ==> 0

29 1 0.333 0 ==> 1

34 1 0.400 0 ==> 2

39 1 0.182 1 ==> 2

42 1 0.250 0 ==> 1

57 1 1.000 0 ==> 1

85 1 0.500 0 ==> 1

86 1 0.167 0 ==> 1

95 1 0.182 1 ==> 2

97 1 0.250 1 ==> 0

105 1 0.100 1 ==> 0

108 1 0.250 0 ==> 1

121 1 0.333 0 ==> 1

node_70 --> Laemanctus longpipes 3 1 0.100 1 ==> 0

22 1 0.143 1 ==> 0

47 1 0.091 0 ==> 1

88 1 0.222 2 --> 1

122 1 0.125 0 ==> 1

node_72 --> Geiseltaliellus maarius 34 1 0.400 0 ==> 1

100 1 0.125 0 ==> 1

101 1 0.500 0 ==> 1

103 1 0.167 1 ==> 0

node_73 --> Suzanniwanna patriciana 18 1 0.200 1 --> 0

node_75 --> node_74 3 1 0.100 1 --> 0

16 1 0.111 0 ==> 1

19 1 0.100 0 ==> 1

22 1 0.143 1 ==> 0

23 1 0.125 1 ==> 0

34 1 0.400 0 ==> 2

38 1 0.250 0 ==> 1

45 1 0.167 1 --> 0

64 1 0.250 0 ==> 1

67 1 0.250 1 --> 2

83 1 0.333 2 --> 0

126 1 0.167 0 ==> 1

135 1 0.125 0 ==> 1

node_74 --> Crotaphytus collaris 47 1 0.091 0 ==> 1

61 1 0.125 1 --> 0

89 1 0.333 0 ==> 1

92 1 0.250 0 ==> 1

94 1 0.182 1 --> 0

95 1 0.182 1 --> 0

117 1 0.167 1 ==> 0

119 1 0.143 1 --> 0

node_74 --> Gambelia wislizenii 17 1 0.500 1 --> 0

105 1 0.100 1 --> 0

106 1 0.125 0 ==> 1

115 1 0.125 1 --> 0

node_69 --> Enyaliodes oshaughnessyi 1 1 0.214 1 --> 0

4 1 0.083 0 ==> 1

6 1 0.333 0 ==> 1

9 1 0.333 0 ==> 1

26 1 0.333 0 ==> 1

40 1 0.250 0 ==> 1

42 1 0.250 0 ==> 1

47 1 0.091 0 ==> 1

62 1 0.143 0 ==> 1

63 1 0.500 0 ==> 1

86 1 0.167 0 ==> 1

88 1 0.222 1 --> 2

91 1 0.250 0 ==> 1

100 1 0.125 0 --> 1

102 1 0.333 0 ==> 1

113 1 0.222 0 --> 2

136 1 0.333 0 ==> 1

node_68 --> node_53 1 1 0.214 1 --> 2

12 1 0.250 0 --> 1

16 1 0.111 0 --> 1

23 1 0.125 1 --> 0

39 1 0.182 1 ==> 0

99 1 0.125 0 --> 1

106 1 0.125 0 ==> 1

111 1 0.333 1 --> 0

113 1 0.222 0 --> 1

138 1 0.200 0 ==> 1

139 1 0.143 0 --> 1

node_53 --> node_49 98 1 0.143 0 ==> 1

125 1 0.167 1 ==> 0

135 1 0.125 0 --> 1

node_49 --> node_44 40 1 0.250 0 ==> 1

53 1 0.125 0 --> 1

66 1 0.143 0 --> 1

72 1 0.400 1 ==> 0

73 1 0.400 1 ==> 2

76 1 0.250 1 --> 0

80 1 0.500 1 --> 2

86 1 0.167 0 --> 1

99 1 0.125 1 --> 0

128 1 0.250 1 ==> 0

139 1 0.143 1 --> 0

node_44 --> node_41 1 1 0.214 2 ==> 0

3 1 0.100 0 --> 1

4 1 0.083 0 --> 1

11 1 0.250 0 ==> 1

13 1 1.000 1 --> 0

16 1 0.111 1 --> 0

19 1 0.100 1 ==> 0

23 1 0.125 0 ==> 1

29 1 0.333 0 ==> 1

31 1 0.222 2 --> 1

37 1 0.200 1 ==> 0

45 1 0.167 0 ==> 1

46 1 0.111 0 ==> 1

47 1 0.091 0 --> 1

51 1 0.250 0 ==> 1

59 1 0.167 0 --> 1

60 1 0.333 0 --> 1

61 1 0.125 0 --> 1

62 1 0.143 0 --> 1

78 1 0.333 0 ==> 1

82 1 0.250 0 ==> 1

87 1 0.333 0 ==> 1

108 1 0.250 0 ==> 1

109 1 0.286 1 ==> 2

114 1 0.286 0 --> 1

116 1 0.400 0 --> 2

117 1 0.167 0 ==> 1

118 1 0.400 0 ==> 1

121 1 0.333 0 ==> 1

132 1 1.000 0 ==> 1

133 1 0.167 1 ==> 0

134 1 0.200 1 ==> 0

147 1 0.500 0 ==> 2

node_41 --> Polychrus acutirostris 6 1 0.333 0 ==> 1

7 1 0.200 0 ==> 1

9 1 0.333 0 ==> 1

12 1 0.250 1 --> 0

28 1 1.000 0 ==> 1

32 1 0.667 0 ==> 1

39 1 0.182 0 ==> 1

92 1 0.250 0 ==> 1

105 1 0.100 0 ==> 1

106 1 0.125 1 ==> 0

111 1 0.333 0 ==> 2

113 1 0.222 1 ==> 2

114 1 0.286 1 --> 2

115 1 0.125 0 ==> 1

122 1 0.125 0 ==> 1

124 1 0.200 0 ==> 1

129 1 0.250 1 ==> 0

node_41 --> node_40 22 1 0.143 1 ==> 0

36 1 0.250 0 ==> 1

50 1 0.429 2 ==> 1

52 1 0.250 0 ==> 2

65 1 0.500 0 ==> 2

66 1 0.143 1 --> 0

75 1 1.000 0 --> 1

77 1 0.500 0 --> 1

85 1 0.500 0 ==> 1

86 1 0.167 1 --> 0

89 1 0.333 0 ==> 1

99 1 0.125 0 --> 1

100 1 0.125 0 ==> 1

139 1 0.143 0 --> 1

141 1 0.333 0 ==> 1

143 1 0.500 0 ==> 1

152 1 0.500 0 --> 1

node_40 --> Anolis ricordi 30 1 0.333 0 ==> 1

59 1 0.167 1 --> 0

101 1 0.500 0 ==> 1

111 1 0.333 0 ==> 1

128 1 0.250 0 ==> 1

node_40 --> Anolis cristatellus 3 1 0.100 1 --> 0

4 1 0.083 1 --> 0

10 1 0.250 1 ==> 0

31 1 0.222 1 --> 2

47 1 0.091 1 --> 0

53 1 0.125 1 --> 0

60 1 0.333 1 --> 0

61 1 0.125 1 --> 0

62 1 0.143 1 --> 0

node_44 --> node_43 67 1 0.250 1 --> 0

71 1 0.500 0 --> 1

94 1 0.182 2 ==> 1

95 1 0.182 2 ==> 1

127 1 0.333 0 ==> 1

130 1 0.333 0 --> 1

146 1 1.000 0 ==> 1

148 1 0.500 0 ==> 1

node_43 --> node_42 7 1 0.200 0 --> 1

76 1 0.250 0 --> 1

123 1 0.167 1 ==> 0

138 1 0.200 1 ==> 0

145 1 0.250 1 ==> 0

node_42 --> Phrynosoma platyrhinos 4 1 0.083 0 ==> 1

12 1 0.250 1 --> 0

15 1 0.500 0 ==> 1

19 1 0.100 1 ==> 0

21 1 0.500 0 ==> 1

25 1 0.333 0 ==> 1

32 1 0.667 0 ==> 2

39 1 0.182 0 ==> 2

42 1 0.250 0 ==> 1

46 1 0.111 0 ==> 1

47 1 0.091 0 ==> 1

51 1 0.250 0 ==> 2

59 1 0.167 0 ==> 1

62 1 0.143 0 ==> 1

88 1 0.222 1 ==> 0

90 1 0.400 1 ==> 0

94 1 0.182 1 ==> 0

95 1 0.182 1 ==> 0

99 1 0.125 0 --> 1

104 1 0.200 0 ==> 1

114 1 0.286 0 ==> 1

115 1 0.125 0 ==> 1

124 1 0.200 0 ==> 1

130 1 0.333 1 --> 0

136 1 0.333 0 ==> 1

node_42 --> Petrosaurus thalassinus 1 1 0.214 2 ==> 1

67 1 0.250 0 --> 1

83 1 0.333 2 ==> 1

109 1 0.286 1 ==> 0

113 1 0.222 1 ==> 0

node_43 --> Sceloporus undulatus 53 1 0.125 1 --> 0

65 1 0.500 0 ==> 2

105 1 0.100 0 ==> 1

120 1 0.125 0 ==> 1

125 1 0.167 0 ==> 1

node_49 --> node_48 12 1 0.250 1 --> 0

113 1 0.222 1 --> 0

node_48 --> node_46 3 1 0.100 0 --> 1

19 1 0.100 1 --> 0

39 1 0.182 0 --> 1

64 1 0.250 0 --> 1

78 1 0.333 0 --> 1

95 1 0.182 2 ==> 1

96 1 0.500 0 ==> 1

105 1 0.100 0 ==> 1

118 1 0.400 0 --> 1

135 1 0.125 1 --> 0

138 1 0.200 1 ==> 0

143 1 0.500 0 ==> 1

node_46 --> Pristidactylus torquatus 1 1 0.214 2 ==> 1

16 1 0.111 1 --> 0

22 1 0.143 1 ==> 0

23 1 0.125 0 ==> 1

37 1 0.200 1 ==> 0

46 1 0.111 0 ==> 1

47 1 0.091 0 ==> 1

51 1 0.250 0 ==> 1

61 1 0.125 0 ==> 1

82 1 0.250 0 ==> 1

103 1 0.167 0 ==> 1

106 1 0.125 1 ==> 0

120 1 0.125 0 ==> 1

126 1 0.167 0 ==> 1

133 1 0.167 1 ==> 0

141 1 0.333 0 ==> 1

147 1 0.500 0 ==> 2

node_46 --> node_45 2 1 0.250 0 --> 1

4 1 0.083 0 --> 1

18 1 0.200 0 --> 1

34 1 0.400 0 --> 2

83 1 0.333 2 ==> 0

104 1 0.200 0 --> 1

113 1 0.222 0 --> 1

117 1 0.167 0 --> 1

118 1 0.400 1 --> 2

125 1 0.167 0 ==> 1

131 1 1.000 0 ==> 1

144 1 0.500 0 ==> 1

node_45 --> Oplurus quadrimaculatus 36 1 0.250 0 ==> 1

39 1 0.182 1 --> 0

62 1 0.143 0 ==> 1

94 1 0.182 2 ==> 1

109 1 0.286 1 ==> 2

127 1 0.333 0 ==> 1

135 1 0.125 0 --> 1

137 1 1.000 0 ==> 1

node_45 --> Chalarodon madagascariensis 3 1 0.100 1 --> 0

19 1 0.100 0 --> 1

27 1 0.200 0 ==> 1

64 1 0.250 1 --> 0

99 1 0.125 1 --> 0

122 1 0.125 0 ==> 1

128 1 0.250 1 ==> 0

node_48 --> node_47 51 1 0.250 0 ==> 2

67 1 0.250 1 --> 2

68 1 0.250 1 --> 0

100 1 0.125 0 ==> 1

109 1 0.286 1 --> 0

140 1 1.000 0 ==> 1

node_47 --> Phymaturus palluma 1 1 0.214 2 ==> 3

2 1 0.250 0 ==> 1

4 1 0.083 0 ==> 1

16 1 0.111 1 --> 0

59 1 0.167 0 ==> 1

86 1 0.167 0 ==> 1

88 1 0.222 1 ==> 2

94 1 0.182 2 ==> 0

95 1 0.182 2 ==> 0

98 1 0.143 1 ==> 0

99 1 0.125 1 --> 0

112 1 0.667 1 ==> 0

123 1 0.167 1 ==> 0

124 1 0.200 0 ==> 1

node_47 --> Liolaemus pictus 36 1 0.250 0 ==> 1

87 1 0.333 0 ==> 1

120 1 0.125 0 ==> 1

122 1 0.125 0 ==> 1

126 1 0.167 0 ==> 1

node_53 --> node_52 50 1 0.429 2 ==> 1

79 1 0.400 2 --> 1

80 1 0.500 1 --> 0

104 1 0.200 0 ==> 1

126 1 0.167 0 ==> 1

127 1 0.333 0 --> 1

130 1 0.333 0 --> 1

145 1 0.250 1 --> 0

147 1 0.500 0 ==> 2

node_52 --> node_50 46 1 0.111 0 ==> 1

120 1 0.125 0 ==> 1

node_50 --> Stenocercus scapularis 1 1 0.214 2 ==> 1

10 1 0.250 1 ==> 0

19 1 0.100 1 ==> 0

37 1 0.200 1 ==> 0

39 1 0.182 0 ==> 1

45 1 0.167 0 ==> 1

48 1 0.333 0 ==> 1

115 1 0.125 0 ==> 1

117 1 0.167 0 ==> 1

122 1 0.125 0 ==> 1

147 1 0.500 2 ==> 1

node_50 --> Microlophus occipitalis 86 1 0.167 0 ==> 1

node_52 --> node_51 7 1 0.200 0 ==> 1

113 1 0.222 1 --> 2

123 1 0.167 1 ==> 0

node_51 --> Tropidurus torquatus 1 1 0.214 2 ==> 3

88 1 0.222 1 ==> 2

node_51 --> Plica umbra 3 1 0.100 0 ==> 1

4 1 0.083 0 ==> 1

10 1 0.250 1 ==> 0

42 1 0.250 0 ==> 1

66 1 0.143 0 ==> 1

98 1 0.143 0 ==> 1

105 1 0.100 0 ==> 1

119 1 0.143 0 ==> 1

node_67 --> node_55 2 1 0.250 0 ==> 1

4 1 0.083 0 --> 1

24 1 1.000 0 ==> 1

40 1 0.250 0 ==> 1

58 1 0.500 0 ==> 1

65 1 0.500 0 ==> 1

90 1 0.400 1 ==> 2

91 1 0.250 0 ==> 1

109 1 0.286 1 ==> 2

116 1 0.400 0 ==> 1

118 1 0.400 0 --> 1

150 1 1.000 0 ==> 1

151 1 0.500 0 ==> 1

node_55 --> Dipsosaurus dorsalis 38 1 0.250 0 ==> 1

50 1 0.429 2 ==> 3

120 1 0.125 0 --> 1

123 1 0.167 1 ==> 0

node_55 --> node_54 3 1 0.100 0 ==> 1

31 1 0.222 2 --> 0

61 1 0.125 0 ==> 1

79 1 0.400 2 ==> 1

105 1 0.100 0 ==> 1

108 1 0.250 0 ==> 1

111 1 0.333 1 --> 2

115 1 0.125 0 ==> 1

133 1 0.167 1 --> 0

134 1 0.200 1 --> 0

node_54 --> Brachylophus fasciatus 4 1 0.083 1 --> 0

67 1 0.250 1 --> 2

node_54 --> Iguana iguana 11 1 0.250 0 ==> 1

31 1 0.222 0 --> 1

51 1 0.250 0 ==> 1

52 1 0.250 0 ==> 2

72 1 0.400 1 ==> 0

103 1 0.167 0 ==> 1

126 1 0.167 0 ==> 1

node_66 --> Leiocephalus personatus 1 1 0.214 1 --> 2

5 1 0.500 0 ==> 1

7 1 0.200 0 ==> 1

39 1 0.182 1 ==> 0

66 1 0.143 0 ==> 1

67 1 0.250 1 --> 2

104 1 0.200 0 ==> 1

106 1 0.125 0 ==> 1

108 1 0.250 0 ==> 1

120 1 0.125 0 --> 1

127 1 0.333 0 ==> 1

138 1 0.200 0 ==> 1

139 1 0.143 0 --> 1

node_65 --> node_59 1 1 0.214 1 --> 3

8 1 1.000 0 ==> 1

14 1 1.000 0 ==> 1

16 1 0.111 0 --> 1

22 1 0.143 1 --> 0

32 1 0.667 0 --> 1

38 1 0.250 0 ==> 1

45 1 0.167 0 ==> 1

61 1 0.125 0 ==> 1

67 1 0.250 1 --> 0

69 1 1.000 0 --> 1

70 1 1.000 0 --> 1

83 1 0.333 1 --> 2

89 1 0.333 0 ==> 1

90 1 0.400 1 --> 0

93 1 0.500 0 ==> 1

100 1 0.125 1 --> 0

103 1 0.167 0 --> 1

109 1 0.286 1 --> 2

115 1 0.125 0 --> 1

119 1 0.143 0 --> 1

127 1 0.333 0 --> 2

145 1 0.250 0 --> 1

149 1 0.500 0 --> 1

node_59 --> node_58 19 1 0.100 1 --> 0

26 1 0.333 0 --> 1

34 1 0.400 0 ==> 2

66 1 0.143 0 ==> 1

97 1 0.250 1 --> 0

99 1 0.125 0 ==> 1

102 1 0.333 0 ==> 1

node_58 --> node_56 20 1 0.500 0 ==> 1

39 1 0.182 1 ==> 0

82 1 0.250 0 ==> 1

84 1 1.000 0 ==> 1

114 1 0.286 0 ==> 2

117 1 0.167 0 ==> 1

121 1 0.333 0 ==> 1

122 1 0.125 1 ==> 0

123 1 0.167 1 ==> 0

node_56 --> Physignathus cocincinus 16 1 0.111 1 ==> 0

46 1 0.111 0 ==> 1

62 1 0.143 0 ==> 1

79 1 0.400 2 --> 0

97 1 0.250 0 --> 1

98 1 0.143 1 --> 0

106 1 0.125 0 ==> 1

112 1 0.667 1 ==> 2

node_56 --> Agama agama 4 1 0.083 0 ==> 1

49 1 0.500 0 ==> 1

68 1 0.250 0 ==> 1

72 1 0.400 1 ==> 0

73 1 0.400 0 --> 1

80 1 0.500 0 ==> 2

119 1 0.143 1 --> 0

124 1 0.200 0 ==> 1

135 1 0.125 0 --> 1

node_58 --> node_57 9 1 0.333 0 --> 1

15 1 0.500 0 ==> 1

22 1 0.143 0 --> 1

31 1 0.222 1 --> 2

50 1 0.429 2 --> 3

58 1 0.500 0 --> 1

63 1 0.500 0 --> 1

79 1 0.400 2 --> 1

90 1 0.400 0 --> 1

node_57 --> Leiolepis belliana 4 1 0.083 0 ==> 1

19 1 0.100 0 --> 1

26 1 0.333 1 --> 0

48 1 0.333 0 ==> 1

49 1 0.500 0 ==> 1

87 1 0.333 0 ==> 1

135 1 0.125 0 --> 1

148 1 0.500 0 ==> 1

node_57 --> Brookesia superciliaris 5 1 0.500 0 ==> 1

23 1 0.125 0 ==> 1

25 1 0.333 0 ==> 1

29 1 0.333 0 ==> 1

38 1 0.250 1 ==> 0

39 1 0.182 1 ==> 2

46 1 0.111 0 ==> 1

47 1 0.091 0 ==> 1

53 1 0.125 0 ==> 1

81 1 0.500 0 ==> 1

107 1 0.500 1 ==> 0

110 1 1.000 0 ==> 1

113 1 0.222 1 ==> 2

114 1 0.286 0 ==> 1

118 1 0.400 0 ==> 1

129 1 0.250 1 ==> 0

138 1 0.200 0 ==> 1

139 1 0.143 0 --> 1

144 1 0.500 0 ==> 1

node_59 --> Priscagama gobiensis 47 1 0.091 0 ==> 1

53 1 0.125 0 ==> 1

105 1 0.100 0 ==> 1

node_63 --> Elgaria multicarinata 7 1 0.200 0 ==> 1

46 1 0.111 0 ==> 1

51 1 0.250 0 ==> 1

71 1 0.500 0 --> 1

73 1 0.400 0 --> 1

78 1 0.333 0 ==> 1

116 1 0.400 0 --> 2

129 1 0.250 1 --> 0

node_62 --> Plestiodon fasciatus 1 1 0.214 0 ==> 1

6 1 0.333 0 ==> 1

20 1 0.500 0 ==> 1

30 1 0.333 0 --> 1

100 1 0.125 1 --> 0

116 1 0.400 0 ==> 1

127 1 0.333 0 ==> 1

node_61 --> Eublepharis macularius 31 1 0.222 0 ==> 1

43 1 0.500 0 ==> 1

44 1 0.500 0 ==> 1

46 1 0.111 0 ==> 1

48 1 0.333 0 ==> 1

51 1 0.250 0 --> 2

66 1 0.143 0 --> 1

82 1 0.250 0 ==> 1

94 1 0.182 0 ==> 2

95 1 0.182 0 ==> 2

104 1 0.200 0 --> 1

114 1 0.286 0 ==> 1

125 1 0.167 0 ==> 1

126 1 0.167 0 ==> 1

node_60 --> Diphydontosaurus avonis 23 1 0.125 1 ==> 0

94 1 0.182 0 ==> 1

95 1 0.182 0 ==> 1

node_60 --> Sphenodon punctatus 1 1 0.214 0 ==> 2

3 1 0.100 0 ==> 1

25 1 0.333 0 --> 1

35 1 0.500 0 ==> 1

45 1 0.167 0 --> 1

52 1 0.250 0 ==> 2

61 1 0.125 0 --> 1

66 1 0.143 0 --> 1

73 1 0.400 0 --> 1

77 1 0.500 0 --> 1

93 1 0.500 0 ==> 1

102 1 0.333 0 --> 1

103 1 0.167 0 --> 1

116 1 0.400 0 --> 2

124 1 0.200 0 --> 1

139 1 0.143 0 --> 1

141 1 0.333 0 --> 1

**8. References:**

1. Simões T.R, *et al*. The origin of squamates revealed by a Middle Triassic lizard from the Italian Alps. *Nature* **557**, 706–709 (2018). <https://doi.org/10.1038/s41586-018-0093-3>

2. Smith K.T. Eocene lizards of the clade *Geiseltaliellus* from Messel and Geiseltal, Germany, and the early radiation of Iguanidae (Reptilia: Squamata). *Bull. Mus. Comp. Zool.* **50**, 219–306 (2009). <https://doi.org/10.3374/014.050.0201>

3. Burbrink F.T *et al*. Interrogating genomic-scale data for Squamata (lizards, snakes, and amphisbaenians) shows no support for key traditional morphological relationships. *Syst. Biol.* **69**, 502-520 (2020). <https://doi.org/10.1093/sysbio/syz062>

4. Streicher J.W, Schulte II J.A & Wiens J.J. How should genes and taxa be sampled for phylogenomic analyses with missing data? An empirical study in iguanian lizards. *Syst. Biol.* **65**, 128–145 (2016). <https://doi.org/10.1093/sysbio/syv058>

5. Zheng Y. & Wiens J.J. Combining phylogenomic and supermatrix approaches, and a time-calibrated phylogeny for squamate reptiles (lizards and snakes) based on 52 genes and 4162 species. *Mol. Phylogenet. Evol*. **94**, 537–547 (2016). <https://doi.org/10.1016/j.ympev.2015.10.009>
